# A Class of Organic Units Featuring Matrix‐Controlled Color‐Tunable Ultralong Organic Room Temperature Phosphorescence

**DOI:** 10.1002/advs.202206482

**Published:** 2022-12-25

**Authors:** Xue Zhang, Chen Qian, Zhimin Ma, Xiaohua Fu, Zewei Li, Huiwen Jin, Mingxing Chen, Hong Jiang, Zhiyong Ma

**Affiliations:** ^1^ Beijing Advanced Innovation Center for Soft Matter Science and Engineering State Key Laboratory of Organic‐Inorganic Composites College of Chemical Engineering Beijing University of Chemical Technology Beijing 100029 China; ^2^ Beijing National Laboratory for Molecular Sciences Key Laboratory of Polymer Chemistry and Physics of the Ministry of Education College of Chemistry and Molecular Engineering Peking University Beijing 100871 China

**Keywords:** guest‐matrix doped system, matrix‐controlled color‐tunability, naphthylamine‐structured organic units, photo‐activated ultralong organic phosphorescence, radical cation

## Abstract

A novel class of organic units (N‐1 and N‐2) and their derivatives (PNNA‐1 and PNNA‐2) with excellent property of ultralong organic room temperature phosphorescence (UORTP) is reported. In this work, N‐1, N‐2, and their derivatives function as the guests, while organic powders (PNCz, BBP, DBT) and polymethyl methacrylate (PMMA) serve as the host matrixes. Amazingly, the color of phosphorescence can be tuned in different states or by varying the host matrixes. At 77 K, all molecules show green afterglow in the monomer state but yellow afterglow in the aggregated state because strong intermolecular interactions exist in the self‐aggregate and induce a redshift of the afterglow. In particular, PNNA‐1 and PNNA‐2 demonstrate distinctive photoactivated green UORTP in the PMMA film owing to the generation of their cation radicals. Whereas the PNNA‐1@PNCz and PNNA‐2@PNCz doping powders give out yellow UORTP, showing matrix‐controlled color‐tunable UORTP. In PNCz, the cation radicals of PNNA‐1 and PNNA‐2 can stay stably and form strong intermolecular interactions with PNCz, leading to a redshift of ultralong phosphorescence.

## Graphical abstract

Two organic units and their derivatives with tunable UORTP in defferent states or by varying doping matrixes are reported. PNNA‐1 and PNNA‐2 demonstrate distinctive photoactived green UORTP in PMMA films. The PNNA‐1@PNCz and PNNA‐2@PNCz doping systems emit yellow UORTP, showing matrix‐controlled color‐tunable UORTP.

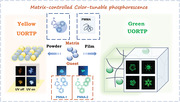

## 1 Introduction

The room temperature phosphorescence (RTP) materials are a category of promising luminescent materials with a large Stokes shift, long‐lived emission, and other peculiar photophysical properties.^[^ ^1^, ^2^, ^3^, ^4^ ^]^ Compared with inorganic phosphorescence materials, pure organic RTP materials are superior in organic light‐emitting diodes (OLEDs),^[^ ^5^, ^6^ ^]^ high‐sensitive chemical sensing,^[^ ^7^ ^]^ information encryption,^[^ ^8^, ^9^ ^]^ high‐resolution biological imaging,^[^ ^10^, ^11^, ^12^ ^]^ data storage^[^ ^13^, ^14^, ^15^ ^]^ with advantages of low cost, simple preparation process, and easy color tunability.^[^ ^16^ ^]^ The new design ideas for constructing UORTP materials have been attracting much attention in recent years. Generally, the phosphorescence performance is closely related to the surrounding environment of phosphors. Rigid environment such as intermolecular interactions and H‐aggregation will greatly suppress the nonradiative decay of triplet excitons, resulting in high quantum yield and long‐lived RTP. Thereby, carbazole,^[^ ^17^, ^18^, ^19^, ^20^, ^21^, ^22^, ^23^ ^]^ triphenylamine (TPA),^[^ ^24^, ^25^ ^]^ phenothiazine,^[^ ^26^ ^]^ and other units^[^ ^27^, ^28^, ^29^ ^]^ with high planar configurations are the mainstream options of building blocks, whereas they conversely impose strict restrictions on the alternatives of UORTP materials meantime.^[^ ^30^, ^31^, ^32^ ^]^ The nature of UORTP materials such as the lifetime and the quantum yield can also be improved by building doping systems, whereas it requires a high energy/charge transfer efficiency between the phosphorescence units and the host materials,^[^ ^33^, ^34^, ^35^, ^36^ ^]^ which strictly limits the selection of the host materials. In our recent work,^[^ ^27^ ^]^ it was found that 1H‐benzo[f]indole (Bd) can be regarded as an excellent phosphorescence unit to fabricate UORTP materials. Through simple doping method, strong UORTP can be achieved from its derivatives, showing a novel approach toward the fabrication of UORTP materials. However, the synthesis of Bd is complicated. To date, few reports about Bd can be found since its first discovery.^[^ ^37^ ^]^ Therefore, it is necessary to explore a simple method for effectively constructing UORTP materials.

**Figure 1:**
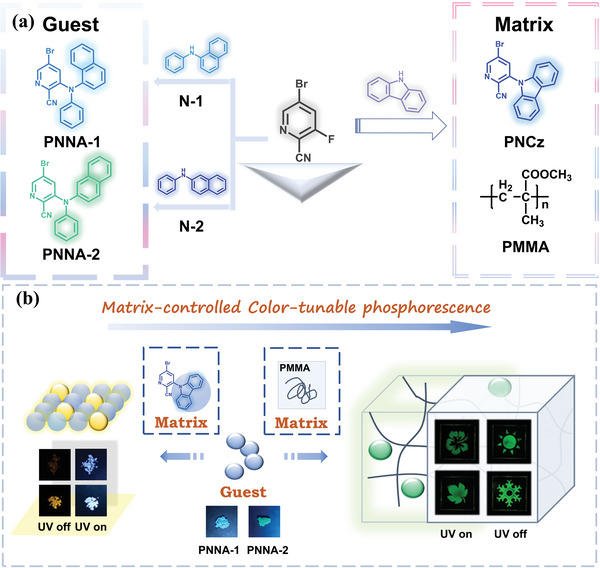
a) Molecular structures of the guests N‐1, N‐2, PNNA‐1, PNNA‐2 and the matrixes PNCz, PMMA. b) Schematic diagram of matrix‐controlled polychromatic phosphorescence emission.

The flexible and adjustable color of luminescence is a significant advantage of UORTP materials. At present, tuning the energy gap between the lowest singlet state and the triplet states (Δ*E*_ST_) by molecular engineering, or changing the crystal packing mode to adjust the phosphorescence color are the common methods of single‐component phosphorescence regulation, which may be tedious regarding preparation process.^[^ ^38^, ^39^, ^40^, ^41^ ^]^ Compared with single‐component phosphorescence materials, the color regulation of doping systems may be realized by matrixes replacement instead of complicated molecular design and single crystal cultivation.^[^ ^42^ ^]^ For the doping systems, ultralong phosphorescence is controlled by the energy/charge transfer efficiency between matrixes and guests,^[^ ^43^, ^44^, ^45^, ^46^ ^]^ which is promising but challenging for the ingenious selection of the matrix and the guest. The design strategy by simply varying the matrix to control the phosphorescence color is charming. It is a promising research direction, and related report is quite rare (**Figure** **1**).

Herein, we report a class of naphthylamine‐structured organic units (N‐1 and N‐2) and their derivatives featuring matrix‐controlled color‐tunable ultralong organic room temperature phosphorescence (UORTP). N‐1 and N‐2 are commercially available and we randomly selected their derivatives PNNA‐1 and PNNA‐2 to support that N‐1 and N‐2 are excellent UORTP units. Excitingly, N‐1, N‐2 and their derivatives show color‐tunable UORTP in different states or in different matrixes. None of the four molecules show ultralong phosphorescence at room temperature whether in the dilute solution (the monomer state) or in the pure powder state (the aggregated state). However, at 77 K, their toluene solutions exhibit bright green ultralong phosphorescence (at 500, 540, 580 nm), while their pure powders display bright yellow ultralong phosphorescence (at 530, 580, 630 nm), showing the redshift of ultralong phosphorescence, which can be attributed to strong intermolecular interactions in the pure powders. More interestingly, all the four molecules demonstrate photoactivated green UORTP when they are dispersed in the PMMA film, and photogenerated cation radicals of N‐1, N‐2, and their derivatives play a vital role in the activation of UORTP. Unexpectedly, when PNNA‐1 and PNNA‐2 are doped into the powder matrix (PNCz), yellow more than green UORTP can be observed because their cation radicals can be stabilized by the matrix and the redshift of ultralong phosphorescence is probably due to that strong intermolecular interactions are formed between the cation radicals and PNCz because of their high molecular structure similarity.^[^ ^47^ ^]^ As PNNA‐1 and PNNA‐2 are dispersed into powder matrixes with low molecular structure similarity (BBP and DBT), green UORTP without redshift appears from the doped systems. To our surprise, N‐1, N‐2, and their derivatives perform much better than Bd (H‐benzo[f]indole) and its derivatives in UORTP. To our best knowledge, N‐1 and N‐2 are a new class of organic units showing matrix‐controlled color‐tunable UORTP. We believe this study will greatly boost the development of organic phosphorescence.

## 2 Results and Discussion

### 2.1 Synthesis and Characterization

The organic units N‐phenyl‐1‐naphthylamine and N‐phenyl‐2‐naphthylamine (named as N‐1 and N‐2) are commercially available. To avoid the influence of trace impurities,^[^ ^37^ ^]^ the purchased N‐1 and N‐2 were purified by column chromatography and recrystallization until white powders were obtained. Then, two target molecules PNNA‐1 and PNNA‐2 with the same donor–acceptor (D–A) structure were designed and synthesized (Schemes S1 and S2, Supporting Information). PNNA‐1 (PNNA‐2) was facilely obtained via the typical substitution reaction between N‐1 (N‐2) and 5‐bromo‐3‐fluoropicolinonitrile. Moreover, we synthesized PNCz through the same substitution reaction with lab‐synthesized Cz and used it as a matrix. The structure and purity of all the molecules including the purified N‐1 and N‐2 were verified by ^1^H NMR, ^13^C NMR, high resolution mass spectrometry (HRMS) and high performance liquid chromatography (HPLC); and the detailed syntheses and molecular characterization are provided in the supporting information (Schemes S1–S3 and Figures S1–S20, Supporting Information).

### 2.2 Photophysical Properties in the Solution and in the Solid State

**Figure 2:**
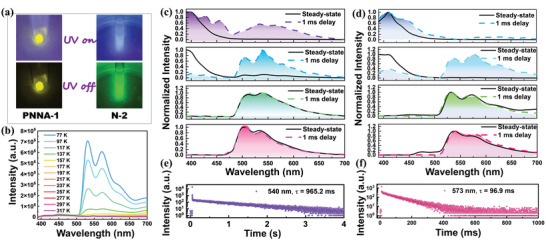
a) Luminescent and phosphorescent images of the PNNA‐1 powder and the N‐2 toluene solution (20 µm) at 77 K. b) Variable‐temperature delayed PL spectra of the PNNA‐1 powder; steady‐state and delayed PL spectra at 77 K of N‐1, N‐2, PNNA‐1, and PNNA‐2 in dilute toluene solution c) and in powder d); decayed spectra at 540 nm of the N‐2 toluene solution e) and at 573 nm of the PNNA‐1 powder f) at 77 K (*λ*_ex_ = 365 nm).

First, the photophysical properties of N‐1, N‐2, PNNA‐1, and PNNA‐2 were studied in the solution (20 µm, **Figure** **2**c). In THF, absorption spectra (Figure S21a, Supporting Information) showed that the *π*–π* transition and the n–π* transition of N‐1, N‐2, PNNA‐1, and PNNA‐2 were located at 256 and 341 nm, 264 and 354 nm, 251 and 313 nm, and 259 and 333 nm, respectively. In the photoluminescence (PL) spectra (Figures S21b,c, Supporting Information), the locally excited (LE) emission of N‐1 and N‐2 appeared at ≈405 nm. PNNA‐1 and PNNA‐2 displayed characteristics of dual emission bands including LE emission and intramolecular charge transfer (ICT) emission in different solvents (e.g., 411 and 435 nm in dichloromethane for PNNA‐1, Figure S21d (Supporting Information); 410 and 429 nm in ethanol for PNNA‐2, Figure S21e, Supporting Information), verifying their D–A structure.

None of N‐1, N‐2, PNNA‐1, and PNNA‐2 exhibited ultralong phosphorescence in the solution at room temperature. However, when the toluene solutions of the four molecules were immersed in the liquid nitrogen, bright green ultralong phosphorescence could be observed by naked eyes (Figure 2a). The delayed PL spectra (Figures S22 and S23, Supporting Information) measured at 77 K showed three ultralong phosphorescence bands at 500 nm (621.7 ms), 533 nm (492.3 ms), and 566 nm (481.2 ms) for N‐1, at 500 nm (960.7 ms), 540 nm (965.2 ms, Figure 2e), and 584 nm (882.8 ms) for N‐2, suggesting N‐1 and N‐2 are phosphorescence active in the monomer state. Similarly, PNNA‐1 and PNNA‐2 showed monomer phosphorescence with green color in the toluene solution at 77 K. In detail, PNNA‐1 displayed three ultralong phosphorescence bands at 510 nm (390.3 ms), 539 nm (380.9 ms), and 583 nm (367.3 ms); PNNA‐2 exhibited three ultralong phosphorescence bands at 507 nm (373.3 ms), 534 nm (319.7 ms), and 585 nm (262.3 ms) (Figures S24 and S25, Supporting Information).

Second, PL properties of pure powders of the four molecules were investigated (Figure 2d). At room temperature, all the four pure powders did not show any afterglow. Interestingly, when they were cooled to 77 K, bright yellow afterglow could be observed clearly (Figure 2a), which was different from the green afterglow of their toluene solutions. At room temperature (Figures S26, S29, S32, and S35, Supporting Information), no long‐lived emission components (over 10 ms) were detected for all the four pure powders, in good agreement with the observed absence of afterglow. At 77 K (Figures S27, S30, S33, and S36, Supporting Information), intense ultralong phosphorescence signal could be detected especially for N‐2, PNNA‐1, and PNNA‐2. Compared with their toluene solutions, the ultralong phosphorescence bands of each pure powder were significantly redshifted, agreeing well with the afterglow color change from green to yellow. For example, the ultralong phosphorescence bands of the pure N‐2 powder appeared at 535 nm (834.4 ms), 577 nm (801.7 ms), 627 nm (693.4 ms) and the redshift was ≈35 nm (Figure S30, Supporting Information); the ultralong phosphorescence bands of the pure PNNA‐1 powder were located at 532 nm (98.6 ms), 573 nm (96.9 ms, Figure 2f), 625 nm, and the redshift was ≈22 nm (Figure S33, Supporting Information).

To assign the phosphorescence of the pure powders, variable‐temperature (delayed) PL spectra (Figure 2b; and Figures S28, S31, and S37, Supporting Information) of the four pure powders were measured. To simplify the description, N‐2 and PNNA‐1 were taken as examples. For N‐2 (Figure S31, Supporting Information), its phosphorescence bands enhanced gradually when the temperature decreased from 317 to 77 K, verifying the nature of ultralong phosphorescence. For PNNA‐1 (Figure 2b), cooling from 317 to 77 K strengthened its ultralong phosphorescence bands remarkably as expected. It is rational that the redshifted phosphorescence of the pure powders is assigned to aggregate phosphorescence and the redshift arises from the enhanced intermolecular interactions in the aggregated state, which is supported by the single‐crystal analysis of PNNA‐1 (Table S3 and Figure S57, Supporting Information).

We tried to cultivate the single crystals of N‐1, N‐2, PNNA‐1, and PNNA‐2 but just successfully obtained the single crystal of PNNA‐1. For the PNNA‐1 single crystal, there are two C—H…*π* (2.703, 2.763 Å) and one C‐Br…*π* (3.560 Å) intermolecular interactions existing in its unit cell. And each PNNA‐1 molecule is locked tightly by four intermolecular interactions from two neighboring molecules. The triplet excitons are well stabilized by strong intermolecular interactions and their energy will decrease to a lower level, leading to the redshift of ultralong phosphorescence in their solid state.

### 2.3 Photoactivated Green UORTP in the PMMA Film

**Figure 3:**
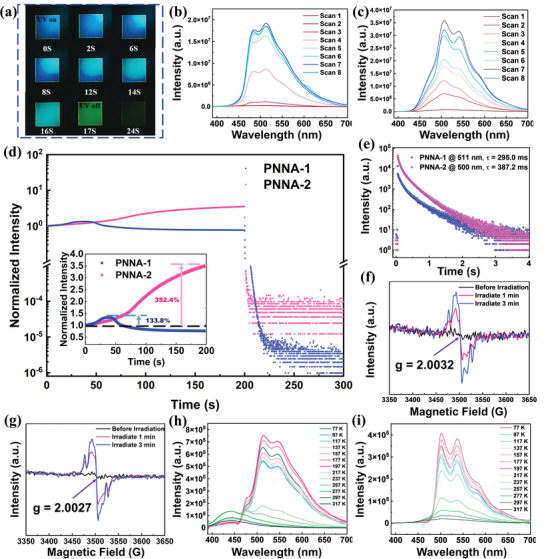
a) Luminescent images of the PNNA‐2 PMMA film taken at different time points for the whole photo‐activated process; photoactivated phosphorescence enhancement of PNNA‐1 b) and PNNA‐2 c) in the PMMA film monitored by steady‐state PL spectra; d) kinetic scannings of the PNNA‐1 and PNNA‐2 films; e) decay spectra at 511 nm of PNNA‐1@PMMA and at 500 nm of PNNA‐2@PMMA after kinetic scanning; ESR spectra of f) N‐1@PMMA and g) N‐2@PMMA before irradiation and after irradiation; variable‐temperature steady‐state PL spectra of h) PNNA‐1@PMMA and i) PNNA‐2@PMMA (*λ*_ex_ = 365 nm).

PMMA is an effective matrix to activate monomer phosphorescence at room temperature.^[^ ^24^, ^26^, ^27^ ^]^ When dispersed into PMMA, the guest molecules are in their monomer state, which significantly avoids self‐aggregation induced quenching. More importantly, the PMMA matrix will encapsulate the guest molecules inside the film. Thus, triplet excitons will be protected from the quenching effect of triplet oxygen. The PNNA‐1@PMMA and PNNA‐2@PMMA films were fabricated through a simple mixing method. The optimized doping ratios were determined as 1 wt% for both PNNA‐1 and PNNA‐2. The as‐prepared film was transparent, and its size could be flexibly adjusted according to various practical needs. The activation process of the PNNA‐2@PMMA film was shown in **Figure** **3**a; and Movie S1 (Supporting Information). The doped film could be activated by a UV portable lamp (16 W). At the beginning of the activation process (0 s), only blue fluorescence could be observed, and ultralong phosphorescence did not appear as the lamp was turned off immediately. With the prolonging of irradiation, the green UORTP began to emerge in 2 s, and the activated area gradually extended when the doped film was further irradiated. The whole activation process lasted for ≈16 s. As the UV lamp was turned off at this moment, strong green UORTP could be observed, and it lasted for more than 7 s.

The delayed PL spectra of PNNA‐1@PMMA and PNNA‐2@PMMA were first measured to systematically study their photoactivation processes at room temperature. As shown in Figure 3b,c, when the doped films were continuously scanned by the spectrometer (450 W, Xenon lamp), a broad phosphorescence band ranging from ≈450 to 650 nm emerged and gradually enhanced from both films of PNNA‐1@PMMA and PNNA‐2@PMMA. After scanning for 8 times, the intensities of phosphorescence for both films reached their maximum. Under these circumstances, the lifetimes of their main phosphorescence peaks (at 511 nm for PNNA‐1@PMMA, and at 500 nm for PNNA‐2@PMMA) were measured as 295.0 and 387.2 ms (Figure 3e), indicating their characteristics of ultralong phosphorescence at room temperature. Furthermore, kinetic scanning was conducted to investigate the whole photoactivated UORTP process. As shown in Figure 3d, intensity of the ultralong phosphorescence peaked at 511 and 500 nm for the PNNA‐1@PMMA and PNNA‐2@PMMA films dramatically grew as the films were irradiated by Xenon lamp (450 W). The intensity rising period of the PNNA‐1@PMMA and PNNA‐2@PMMA films lasted for 30 and 200 s, respectively, and the corresponding rising percentages were calculated as 133.8% and 352.4%. For the PNNA‐1@PMMA film, an additional quenching process could be observed when its intensity reached maximum, which may be attributed to the excessive molecular configuration flip of the PNNA‐1 molecules when dispersed in the PMMA matrix.

To gain a deeper insight into the phosphorescence mechanism, electron spin resonance (ESR) experiments were performed during the photoactivation process of the PNNA‐1@PMMA and PNNA‐2@PMMA films (Figure S52, Supporting Information) at room temperature. Before irradiation, no ESR signals could be observed from both doped films. When the doped films were irradiated for 1 min, strong ESR signals arose, and the g values were calculated as 2.0060 and 2.0044 for the PNNA‐1@PMMA and PNNA‐2@PMMA films, respectively, which were very close to a free electron (2.0065). And further irradiation could enhance their ESR signals, indicating that radical cations were generated and accumulated during the photoactivation process. Variable‐temperature steady‐state PL spectra were also recorded (Figure 3h,i; and Figures S43 and S46, Supporting Information). The intensity of emerging green ultralong phosphorescence dramatically rose with the decreasing temperature, verifying their phosphorescence nature.

The mechanism of photoactivated ultralong phosphorescence can be reasonably deduced as follows: as the doped film is irradiated by UV light, electrons of the dispersed guest molecules will rapidly transfer to the PMMA matrix, generating radical cations. Meanwhile, oxygen present in the doped film is consumed by the triplet excitons. Owing to encapsulation effect and rigid environment provided by PMMA, nonradiative decay processes are greatly suppressed, and external oxygen is hard to permeate into the doped film. Thus, the generated triplet excitons can accumulate during the irradiation process. More importantly, Due to the charge‐separated states formed by guest radical cations and matrix radical anions, radiative decay process of the triplet excitons is further prolonged, resulting in strong and long‐lived phosphorescence.

### 2.4 Color‐Tunable UORTP in Different Powder Matrixes

**Figure 4:**
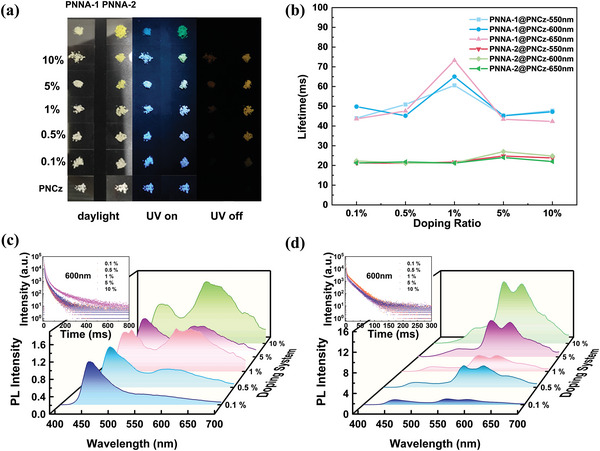
a) Daylight, luminescent, and phosphorescent images of the PNNA‐1, PNNA‐2, PNCz, PNNA‐1@PNCz, and PNNA‐2@PNCz powders. b) Lifetimes at 550, 600, and 650 nm changed along with the dopant ratio for PNNA‐1@PNCz and PNNA‐2@PNCz; delayed PL spectra at different dopant ratio for the doped system c) PNNA‐1@PNCz and d) PNNA‐2@PNCz; decay spectra of (c, upper left) PNNA‐1@PNCz and (d, upper left) PNNA‐2@PNCz with different ratio (*λ*_ex_ = 365 nm).

As we discussed above, the N‐1, N‐2, PNNA‐1, and PNNA‐2 powders did not emit ultralong phosphorescence until the temperature reached 77 K. However, as PNNA‐1 and PNNA‐2 were used as the guest molecules to construct doped systems, remarkable UORTP could be observed (Figures S53 and S54, Tables S1 and S2, Supporting Information). PNCz was chosen as the matrix powder for it has a resemblant chemical structure with the guest molecule, which is beneficial to charge transfer among molecules of the matrix and the guest.^[^ ^47^ ^]^ At room temperature, the PNCz powder showed blue emission peaked at 450 nm with a lifetime of 6.5 ms, and a weak shoulder band ranging from ≈550 to 650 nm with a lifetime of 11.1 ms (Figure S38, Supporting Information), indicating that it does not achieve visible yellow ultralong phosphorescence at room temperature (**Figure** **4**a). However, when PNNA‐1 and PNNA‐2 were dispersed into the PNCz powder at low concentrations (0.1–10 wt%) separately, yellow UORTP could be observed from the PNNA‐1@PNCz and PNNA‐2@PNCz doped powders. With the mixing of PNNA‐1 or PNNA‐2, three new phosphorescence bands peaked at 550, 600, and 650 nm emerged, and increased dramatically when the concentration of the guest molecules increased (Figure 4c,d), which were very similar to the intrinsic phosphorescence of PNNA‐1 and PNNA‐2 in their aggregated states at low temperature. Meanwhile, the lifetimes of the doped systems altered with the doping ratio of guest molecules (Figure 4b). The optimized doping ratios of the PNNA‐1@PNCz and PNNA‐2@PNCz doped systems were determined as 1 and 5 wt%, and the corresponding lifetimes (peaked at 600 nm) were measured as 65.0 and 27.0 ms, respectively (Tables S1 and S2, Supporting Information).

Likewise, ESR spectra were measured to search the evidence of radical cations. At ambient condition, all pure N‐1, N‐2, PNNA‐1, and PNNA‐2 powders showed ESR signals (Figure S39, Supporting Information), and their g values were calculated to be around 2.0065, indicating that the generated radical cations originated from the guest molecules. It is also noteworthy that the ESR signals did not change either before or after irradiation, suggesting that radical cations could stay stably within powder matrixes without photoactivation. However, due to the strong self‐quenching effect, no yellow ultralong phosphorescence could be observed by naked eyes at room temperature. As the environment temperature decreased to 100 K, nonradiative decay of triplet excitons of the radicals was prohibited (Figure S40, Supporting Information), so the yellow ultralong phosphorescence was visible from their pure powders (Figure 2a). Meanwhile, intensity of ESR signals also enhanced under this circumstance, and their g values remained unchanged. As PNNA‐1 and PNNA‐2 were dispersed into the PNCz powder to construct doped systems, ESR signals were also detected from the PNNA‐1@PNCz and PNNA‐2@PNCz doped powders (Figure S55, Supporting Information), indicating that the PNCz powder can also serve as an effective matrix to stabilize generated radical cations of the guests and their triplet excitons, so that the yellow ultralong phosphorescence can be observed from the PNNA‐1@PNCz and PNNA‐2@PNCz doped powders (Figure 4a). Moreover, the redshift of ultralong phosphorescence color change from green to yellow might result from enhanced intermolecular interactions between cation radicals of the guest and the matrix molecules because of their high molecular structure similarity according to the single‐crystal analysis of PNCz (Tables S3 and S58, Supporting Information).

**Figure 5:**
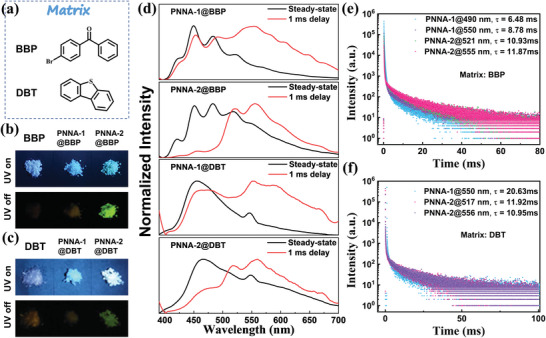
a) Molecular structures of BBP and DBT. b) Photoluminescent and phosphorescent images of BBP, PNNA‐1@BBP and PNNA‐2@BBP. c) Photoluminescent and phosphorescent images of DBT, PNNA‐1@DBT and PNNA‐2@DBT. d) Steady‐state and delayed (1 ms) PL spectra of the four doped powders (PNNA‐1@BBP and PNNA‐2@BBP, PNNA‐1@DBT, and PNNA‐2@DBT); decay spectra of PNNA‐1 and PNNA‐2 in (e) BBP and (f) DBT (*λ*_ex_ = 365 nm, all spectra were measured at ambient condition).

In addition, we studied the UORTP properties of PNNA‐1 and PNNA‐2 in other powder matrixes such as BBP and DBT (**Figure** **5**). It is found that BBP and DBT can also function as effective matrixes to activate the ultralong phosphorescence of PNNA‐1 and PNNA‐2 at room temperature and their cation radicals can also be well stabilized by BBP and DBT (Figure S56, Supporting Information). Different from PNCz, PNNA‐1, and PNNA‐2 both showed green UORTP in BBP and DBT, probably because they have a low molecular structure similarity with BBP and DBT, further showing the unique property of matrix‐controlled color‐tunable UORTP. Moreover, the lifetimes of the phosphorescence bands in the doped systems (PNNA‐1@BBP, PNNA‐1@DBT, PNNA‐2@BBP, and PNNA‐2@DBT) were shorter than those in PNNA‐1@PNCz and PNNA‐2@PNCz correspondingly, suggesting that the guest molecules form weaker intermolecular interactions with BBP and DBT.

### 2.5 TD‐DFT Calculations and Proposed Mechanism of the UORTP

Furthermore, time‐dependent density functional theory (TD‐DFT) calculations were done to elucidate the cation radical‐involved phosphorescence mechanism. According to the spin–orbital coupling results, the values of PNNA‐1 and PNNA‐2 are much higher than those of N‐1 and N‐2 (Table S8, Supporting Information), agreeing well with the fact that D–A structure and heavy atom effect greatly promote intersystem crossing (ISC) efficiency. It is shown that the energy levels of the excited states of their corresponding cation radicals are significantly lower than T*_n_* of PNNA‐1 and PNNA‐2 (Tables S4–S7, and S9, Supporting Information). Thus, it is rational that the green ultralong phosphorescence over 500 nm originates from the cation radicals. Since N‐1 and N‐2 do not have definite D‐A structure, their emission bands are generally assigned to locally excited (LE) emission because both the highest occupied molecular orbitals (HOMO) and the lowest unoccupied molecular orbitals (LUMO) are immobile on the skeleton of N‐1 and N‐2 (Figures S63 and S64, Supporting Information). Similarly, the green ultralong phosphorescence bands of N‐1^•+^ and N‐2^•+^ are also ascribed to their LE emissions as HOMO and SUMO are localized on the structure of N‐1^•+^ and N‐2^•+^ (Figures S63 and S64, Supporting Information). Furthermore, PNNA‐1 and PNNA‐2 show definite D–A structures because their HOMO and LUMO are distributed on the donor and the acceptor (Figures S65 and S66, Supporting Information), respectively. Surprisingly, the SUMO and HOMO of PNNA‐1^•+^ (PNNA‐2^•+^) are both dispersed on the structure of N‐1 (N‐2) owing to its highly distorted geometry, suggesting that the green ultralong phosphorescence of PNNA‐1^•+^ (PNNA‐2^•+^) arise from the LE triplet excitons of N‐1 (N‐2). Therefore, PNNA‐1^•+^ (PNNA‐2^•+^) and N‐1^•+^ (N‐2^•+^) almost have the same ultralong phosphorescence bands, agreeing well with the experimental data.

**Figure 6:**
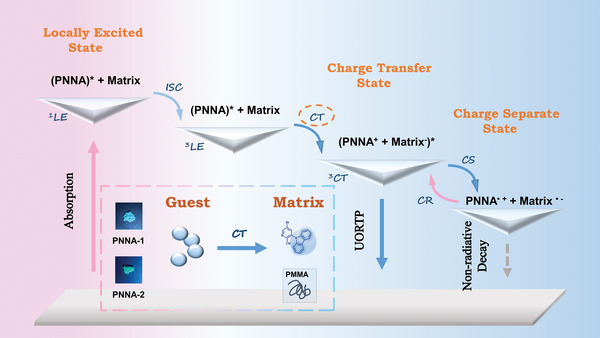
Proposed ultralong phosphorescence mechanism for N‐1, N‐2 and their derivatives.

On the basis of all the data above and previous work,^[^ ^27^, ^44^ ^]^ we proposed a cation radical‐involved mechanism featuring charge separation and charge recombination for the ultralong phosphorescence of N‐1, N‐2, and their derivatives (**Figure** **6**). Six steps are involved in the proposed ultralong phosphorescence mechanism. To simplify the description, PNNA is used to represent N‐1, N‐2, PNNA‐1, and PNNA‐2. At step 1, the guest PNNA is excited to the locally excited singlet state (^1^LE). At step 2, the singlet exciton transfers to the locally excited triplet state (^3^LE) through ISC. At step 3, the charge transfer triplet state (^3^CT) forms when charge transfer occurs from PNNA to the matrix. At step 4, the triplet exciton is trapped by charge separate state due to its lower energy level. At step 5, when charge recombination happens, the triplet exciton returns to the ^3^CT state. Finally, the triplet exciton jumps to the ground state from the ^3^CT state, emitting ultralong phosphorescence.

### 2.6 Comparison among N‐1, N‐2, and Bd

According to our previous research^[^ ^27^ ^]^ and Prof. Bin Liu's work,^[^ ^37^ ^]^ Bd (1H‐benzo[f]indole) should be recognized as an important (or the first) organic phosphorescence unit as Bd and its derivatives show the characteristics of ultralong phosphorescence as below. 1) Bd derivatives showed photo‐activated UORTP in the PMMA film though it is difficult to activate the ultralong phosphorescence of Bd at the same condition. 2) The UORTP property of Bd and its derivatives can also be activated by matrixes including carbazole‐based molecules. 3) The ultralong phosphorescence of Bd and its derivatives arises from their corresponding cation radicals.

**Figure 7:**
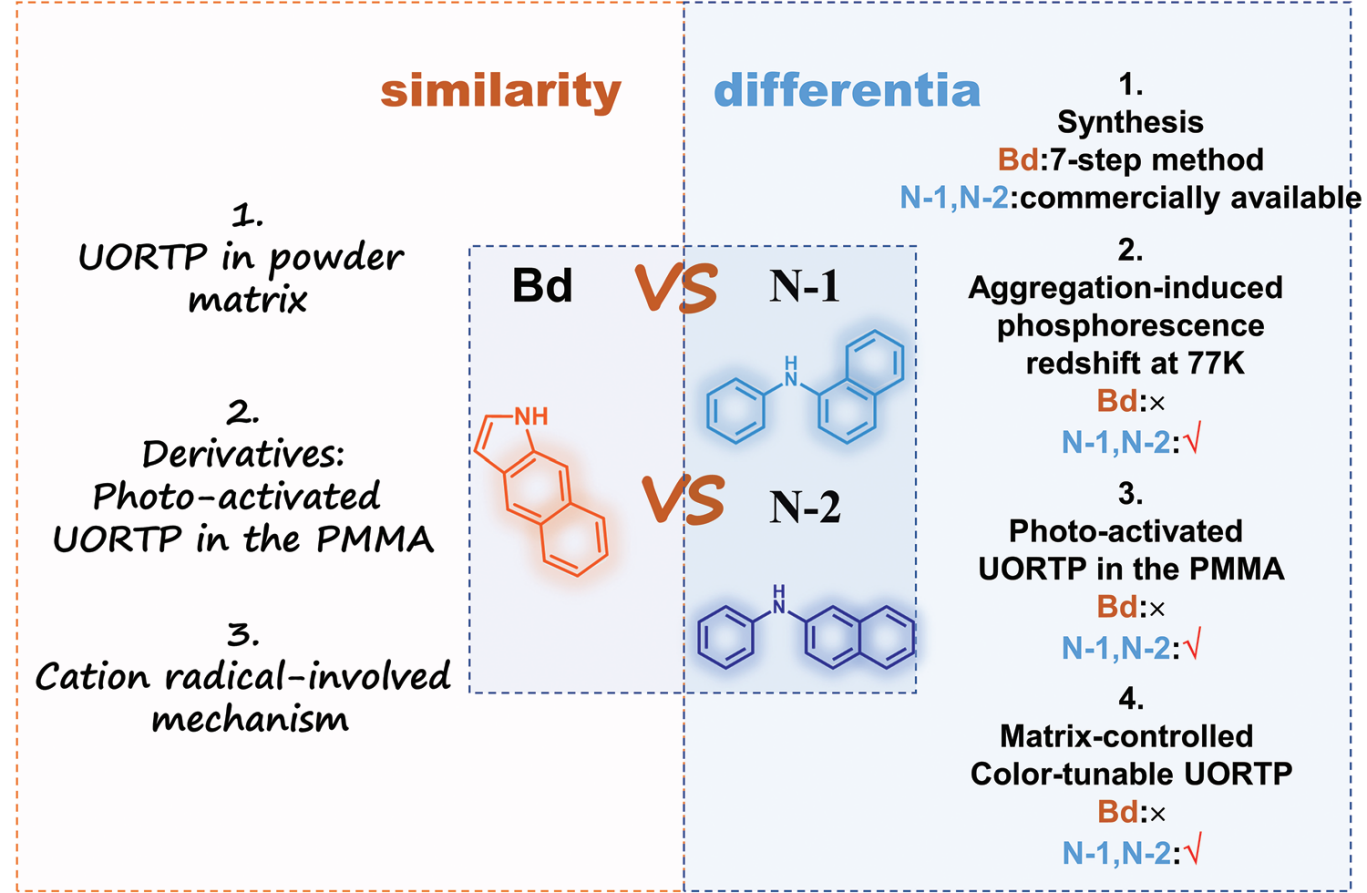
Comparison of ultralong phosphorescence properties of Bd, N‐1, N‐2 and their derivatives in different states and at different conditions.

To clearly compare the ultralong phosphorescence of Bd and our new units (N‐1 and N‐2), we made a table to list ultralong phosphorescence properties of Bd, N‐1 and N‐2 in different states and at different conditions (**Figure** **7**). It is clear that N‐1, N‐2, and their derivatives perform much better than Bd and its derivatives in UORTP. In particular, N‐1 and N‐2 are commercially available while the synthesis of Bd is rather tedious. The derivatives of N‐1 and N‐2 show matrix‐controlled color‐tunable UORTP, which has never been observed in the Bd‐based systems. Therefore, N‐1 and N‐2 are a novel class of organic phosphorescence units, which should be recognized as a big advancement in the field of organic phosphorescence.

### 2.7 Application Demonstration

**Figure 8:**
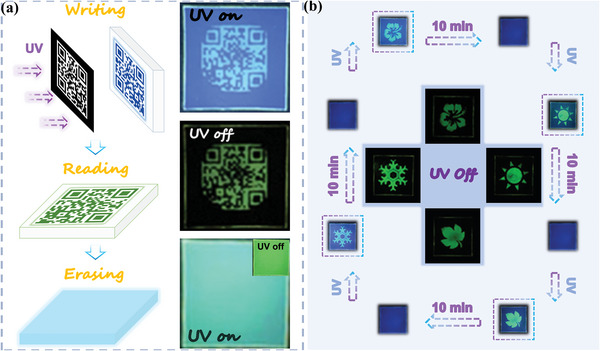
Demonstration of applications based on the photo‐activated PNNA‐2@PMMA films (1 wt%). a) Information coding: Procedure (left) and photographs (right) of the PNNA‐2@PMMA film with photolithographic QR code pattern. b) High‐resolution photolithographic patterning of the PNNA‐2@PMMA film with fast writing and time‐dependent self‐erasing properties (*λ*_ex_ = 365 nm).

UV printing is currently a green and rapidly developing printing technology. Based on the special photophysical properties of the PNNA‐2@PMMA doped system, it can realize high‐resolution UV printing, and also the printed patterns can be repeatedly written and erased. The UV printing process is roughly divided into three steps including writing, reading, and erasing. As shown in the schematic diagram on the left of **Figure** **8**a, in the first step, the QR code was printed in black and white on a piece of A4 paper as a mask, and a portable UV lamp was used to illuminate the film through the mask for 5 min so the uncovered part could be fully activated. The second step was reading. As the film was irradiated with an UV lamp, the QR code was gradually activated on the film. The last step was erasing, and the continued irradiation could fully activate the ultralong phosphorescence of the doped film. Practical application is shown in the pattern on the right of Figure 8a; and Movie S2 Supporting Information). A QR code was activated by UV and printed on the film. The green phosphorescent QR code pattern could be clearly read after the UV lamp was turned off, and this information could even be recognized by cell phones, indicating the high‐resolution property of UV printing of this doped film system.

The reusability of printing materials is crucial to the development of UV printing technology. As shown in Figure 8b, the doped PMMA film has good reproducible writing and erasing ability. The doped film still retained faint traces of the QR code when it was reactivated after 1 week natural recovery in the dark. It can be seen that this PMMA doping system has a good long‐term storage ability for UV printing patterns. In order to improve the reusability of the doped film, the influence of the previous pattern on the next pattern must be completely eliminated. So, we heated the doped film for 10 min to completely erase the UV traces of the previous pattern on the film. We used this recovery method to repeatedly print four different patterns of “spring,” “summer,” “autumn,” and “winter” on the same doped film. Our work has greatly improved the availability of special printing materials, and application of the light‐sensitive properties of the doped PMMA system in UV printing technology is broadened.

## 3 Conclusion

In summary, we report a novel class of organic units (N‐1 and N‐2) and their derivatives (PNNA‐1 and PNNA‐2) with excellent UORTP property. Amazingly, color of the ultralong phosphorescence can be tuned in different states or by varying the doping matrixes. At 77 K, all the four molecules show green afterglow in the monomer state but yellow afterglow in the aggregated state because strong intermolecular interactions exist in the self‐aggregate and induce the redshift of the afterglow. In particular, PNNA‐1 and PNNA‐2 demonstrate distinctive photo‐activated green UORTP in the PMMA film owing to the generation of their cation radicals. Surprisingly, the PNNA‐1@PNCz and PNNA‐2@PNCz doping systems give out yellow UORTP, showing matrix‐controlled color‐tunable UORTP. In PNCz, the cation radicals of PNNA‐1 and PNNA‐2 can stay stably and form strong intermolecular interactions with PNCz because of the high similarity of their molecular structures, leading to the redshift of ultralong phosphorescence. Additionally, PNNA‐1 and PNNA‐2 show green UORTP in the DBT and BBP probably because they have a low molecular structure similarity with DBT and BBP. Compared with previously reported Bd (H‐benzo[f]indole) and its derivatives, N‐1, N‐2, and their derivatives behave much better in UORTP. This study provides another example to support that cation radical might be a universal mechanism in organic phosphorescence. We believe that this work will expand the scope of organic phosphorescence.

## 4 Experimental Section

### Preparation of PMMA Films

A mixture powder of PMMA (300 mg) and N‐1 (3 mg) was dissolved in dichloromethane (2 mL) at room temperature. All solutions were colorless and transparent, and the guests were molecularly dispersed into solvent. Then the solution was poured into the prepared mold and dried for 30 min at ambient condition. As the solvent was completely removed, a transparent, large‐area and photoprintable film can be obtained from the mold. Similarly, the N‐2, PNNA‐1, PNNA‐2, and PNCz films can also be obtained in this method.

### Procedure of Photoprinting

First, a pattern was printed on a piece of A4 paper in black and white by printer. Then the pattern was placed above the doped film. When the UV flashlight (365 nm, 16 W) illuminated above the paper, the film under the white part of the paper can be irradiated by UV light. Thus, the pattern can be printed on the doped film. After removing UV light, the printed pattern on the film will show green ultralong afterglow, which leads to the high resolution.

### Data Availability 

All data that support the findings of this study are available in the online version of this paper in the accompanying Supplementary Information (including experimental methods/procedures, synthetic routes, molecular characterization of N‐1/N‐2/PNNA‐1/PNNA‐2/PNCz, photophysical properties in solution and in the solid state, PL spectra of the doped PMMA films, PL spectra of the doped powders, single crystal data, TD‐DFT results).

## Conflict of Interest

The authors declare no conflict of interest.

## Author Contributions

X.Z., C.Q., Z.M.M., X.H.F., Z.W.L., and H.W.J. performed the experiments and prepared the Supporting Information. Z.Y.M. conceived and directed the project. Z.Y.M. and Z.M.M. wrote the manuscript. M.X.C. and H.J. helped modified the manuscript.

## Supporting information

Supporting Information

Supplemental Movie 1

Supplemental Movie 2

Supporting Information

Supporting Information

## Data Availability

The data that support the findings of this study are available in the supplementary material of this article.
