# Supplementary material for: A Class of Organic Units Featuring Matrix‐Controlled Color‐Tunable Ultralong Organic Room Temperature Phosphorescence
Source: Adv Sci (Weinh). 2022 Dec 25;10(3):2206482. doi: 10.1002/advs.202206482 (PMC9875667; doi:10.1002/advs.202206482)
Supplement: Supplementary file 1 — Supporting Information [file ADVS-10-2206482-s004.pdf]

ADVANCED SCIENCE
Open Access

Supporting Information

for *Adv. Sci.*, DOI 10.1002/advs.202206482

A Class of Organic Units Featuring Matrix-Controlled Color-Tunable Ultralong Organic Room Temperature Phosphorescence

*Xue Zhang, Chen Qian, Zhimin Ma, Xiaohua Fu, Zewei Li, Huiwen Jin, Mingxing Chen, Hong Jiang and Zhiyong Ma**

Supporting Information

A Class of Organic Units Featuring Matrix-controlled Color-tunable Ultralong Organic Room Temperature Phosphorescence

Authors: Xue Zhang, Chen Qian, Zhimin Ma, Xiaohua Fu, Zewei Li, Huiwen Jin, Mingxing Chen, Hong Jiang and Zhiyong Ma*

Affiliations:

X. Zhang, C. Qian, X. Fu, H. Jin, Prof. Z.Y. Ma
Beijing Advanced Innovation Center for Soft Matter Science and Engineering
State Key Laboratory of Organic-Inorganic Composites
College of Chemical Engineering
Beijing University of Chemical Technology
Beijing 100029, China
E-mail: mazhy@mail.buct.edu.cn

Dr. Z.M. Ma, Z. Li, Dr. M. Chen, Prof. H. Jiang
Beijing National Laboratory for Molecular Sciences
Key Laboratory of Polymer Chemistry and Physics of the Ministry of Education
College of Chemistry and Molecular Engineering
Peking University
Beijing 100871, China

Xue Zhang and Chen Qian contributed equally to this work.

1. Materials and General Methods

All the solvents and reactants were purchased from commercialized companies and used as received without further purification except for specifying otherwise.

^{1}H NMR was recorded on the 400 MHz (Bruker ARX400) and ^{13}C NMR spectra were recorded on the Bruker 101 MHz spectrometer at room temperature with $CDCl_3$ and DMSO-d_6 as the solvent and tetramethylsilane (TMS) as the internal standard. ESI high resolution mass-spectra (HRMS) were acquired on a Waters Xevo G2 Qt of mass spectrometer. High Performance Liquid Chromatography (HPLC) was acquired on Shimadzu LC-20AT. The elution procedure was optimized as 65/35 (v/v) of acetonitrile/water for 30 min at 1.00 mL/min The type of chromatographic column was ZORBAX SB-C_{18} (Agilent, 150 mm × 4.6 mm, 5 μm). Transient and delayed photoluminescence spectra were performed on the Hitachi F-4600 or Edinburgh Instruments FLS980 fluorescence spectrophotometer (Kinetic scanning and photo-activated process slit: 20/15 nm). Luminescence lifetime were acquired on the Edinburgh Instruments FLS980 fluorescence spectrophotometer or Deltaflex Fluorescence Lifetime Instrument (λ_{ex} = 365 nm). Single crystal X-ray diffraction data were collected with a NONIUS KappaCCD diffractometer with graphite monochromator and Mo Kα radiation [λ (MoKα) = 0.71073 Å]. Structures were solved by direct methods with SHELXS-97 and refined against F2 with SHELXS-97.

TD-DFT calculations were conducted on Gaussian 09 program with a method similar to previous literature.[1] Ground state (S_0) geometries of N-1, N-2, PNNA-1 and PNNA-2 monomer were directly optimized in vacuum condition. On the basis of this, exciton energies in singlet (S_n) and triplet states (T_n) were estimated through a combination of TDDFT and B3LYP at the 6-311+G (p, d) level. Kohn-Sham frontier orbital analysis was subsequently performed based on the results of theoretical calculation to elucidate the mechanisms of possible singlet-triplet intersystem crossings, in which the channels from S_1 to T_n are believed to share part of the same transition orbital compositions. Herein, energy levels of the possible T_n states are considered to lie within the range of ES_1±0.3 eV.[2] Spin-orbital couplings (SOC) matrix elements were conducted through the Beijing Density Functional (BDF) program based on optimized or single crystal structures at the B3LYP/6- 311G* level.

2. **Syntheses and characterizations**

Lab-Cz was synthesized from aniline and o-bromoiodobenzene through two-step reaction according to the procedures in our previous work.[3] N-1 and N-2 were purchased from commercial company. To avoid the influence of trace impurities, all raw materials were purified by column chromatography and recrystallization, and their structures and purity were testified by ^{1}H NMR, ^{13}C NMR and HRMS. The detailed syntheses of PNNA-1, PNNA-2 and PNCz are shown as follows.

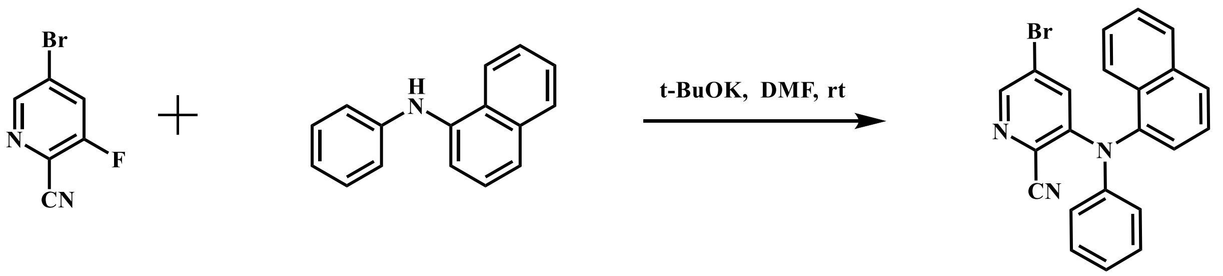

Scheme S1. The synthetic route to PNNA-1.

PNNA-1: A mixture of 5-Bromo-3-fluoropicolinonitrile (0.3015 g, 1.5 mmol), N-phenyl-1-naphthylamine (0.2741 g, 1.25 mmol) and t-BuOK (0.1403 g, 1.25 mmol) were dissolved in DMF (2 mL) in a Schlenk bottle and was stirred under N_2 atmosphere for 24 h at room temperature. After the reaction was completed, the resultant mixture was cooled down to room temperature and the solvent was removed under reduced pressure. The crude product was purified by column chromatography using dichloromethane and petroleum ether (v/v, 1:2) as the eluent to obtain pure product as white powder. Yield: 12%.

^{1}H NMR (400 MHz, DMSO-d_6) δ (ppm): 8.47 (d, J = 1.9 Hz, 1H), 7.99 (dd, J = 22.9, 8.1 Hz, 2H), 7.83 (d, J = 8.3 Hz, 1H), 7.60 – 7.30 (m, 7H), 7.18 – 7.03 (m, 3H).

^{13}C NMR (101 MHz, DMSO-d_6) δ (ppm) : 149.70, 146.58, 145.55, 141.53, 135.24, 133.82, 130.38, 130.24, 129.18, 128.44, 127.57, 126.82, 125.34, 125.07, 124.40, 123.44, 115.88.

HR-ESI-MS Calcd. For $C_{22}H_{14}BrN_3$ $[M+H]^+$: 400.0449. Found: 400.0443.

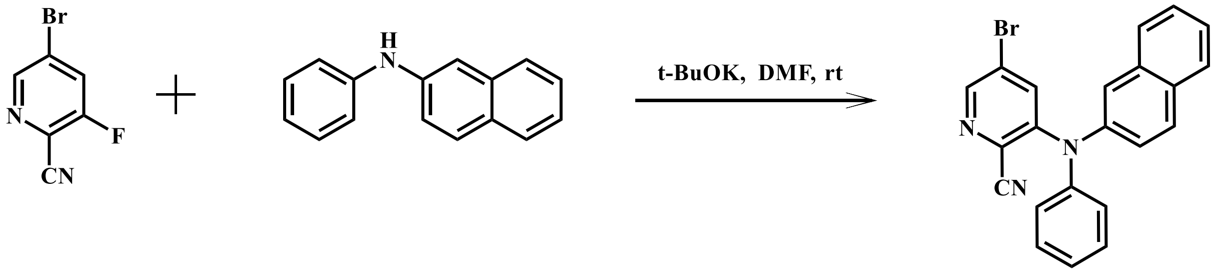

Scheme S2. The synthetic route to PNNA-2.

PNNA-2: A mixture of 5-Bromo-3-fluoropicolinonitrile (0.3015 g, 1.5 mmol), N-phenyl-2-naphthylamine (0.2741 g, 1.25 mmol) and t-BuOK (0.1403 g, 1.25 mmol) were dissolved in DMF (2 mL) in a Schlenk bottle and was stirred under N_2 atmosphere for 24 h at

room temperature. After the reaction was completed, the resultant mixture was cooled down to room temperature and the solvent was removed under reduced pressure. The crude product was purified by column chromatography using dichloromethane and petroleum ether (v/v, 1:2) as the eluent to obtain pure product as yellow powder. Yield: 18%.

^{1}H NMR (400 MHz, DMSO-d_6) δ (ppm) : 8.59 (d, J = 1.9 Hz, 1H), 7.96 – 7.84 (m, 3H), 7.82 – 7.76 (m, 1H), 7.56 (d, J = 2.1 Hz, 1H), 7.51 – 7.35 (m, 4H), 7.29 – 7.11 (m, 4H).

^{13}C NMR (101 MHz, DMSO-d_6) δ (ppm) : 148.79, 146.02, 143.79, 136.88, 134.27, 130.98, 130.47, 130.23, 128.11, 127.82, 127.22, 127.03, 125.64, 125.29, 124.95, 124.15, 121.49, 116.01.

HR-ESI-MS Calcd. For $C_{22}H_{14}BrN_3$ $[M+H]^+$: 400.0449. Found: 400.0447.

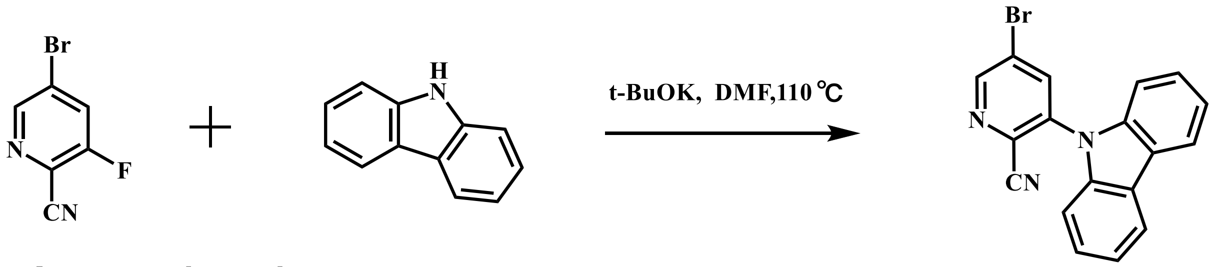

Scheme S3. The synthetic route to PNCz.

PNCz: A mixture of Carbazole (0.2508 g, 1.5 mmol), 5-Bromo-3-fluoropicolinonitrile (0.3618 g, 1.8 mmol) and t-BuOK (0.1683 g, 1.5 mmol) were dissolved in DMF (2 mL) in a Schlenk bottle and was stirred under N_2 atmosphere for 24 h at 110 °C. After the reaction was over, the resultant mixture was cooled down to room temperature and the solvent was removed under reduced pressure. The crude product was purified by column chromatography using dichloromethane and petroleum ether (v/v, 1:4) as the eluent to obtain pure product as white powder. Yield: 31%.

^{1}H NMR (400 MHz, $CDCl_3$) δ (ppm) : 8.89 (d, J = 2.0 Hz, 1H), 8.20 – 8.11 (m, 3H), 7.50 – 7.45 (m, 2H), 7.41 – 7.36 (m, 2H), 7.22 (d, J = 8.2 Hz, 2H).

^{13}C NMR (101 MHz, $CDCl_3$) δ (ppm) : 151.36, 140.13, 139.82, 139.25, 131.30, 126.69, 124.49, 121.87, 120.90, 114.64, 109.51.

HR-ESI-MS Calcd. For $C_{18}H_{10}BrN_3$ $[M+H]^+$: 348.013091. Found: 348.013086.

3. **NMR spectra and HR-MS of mentioned molecules.**

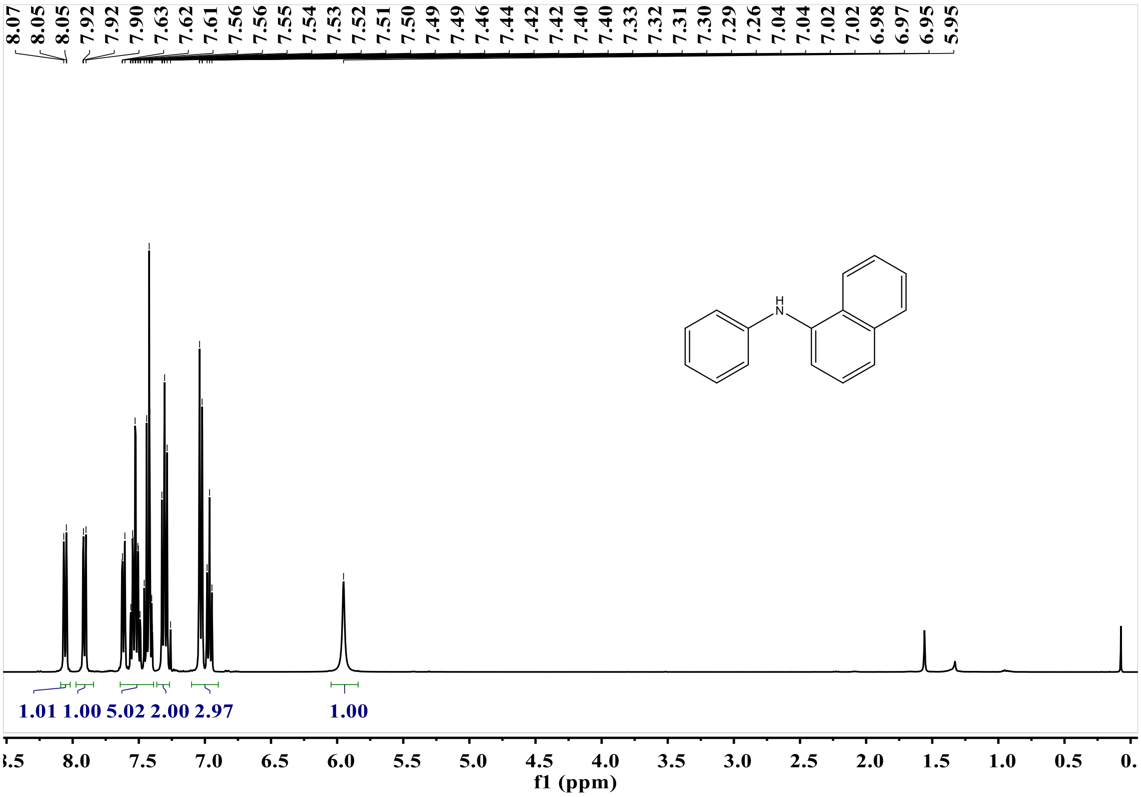

Figure S1. ^{1}H NMR spectrum of N-1 in $CDCl_3$.

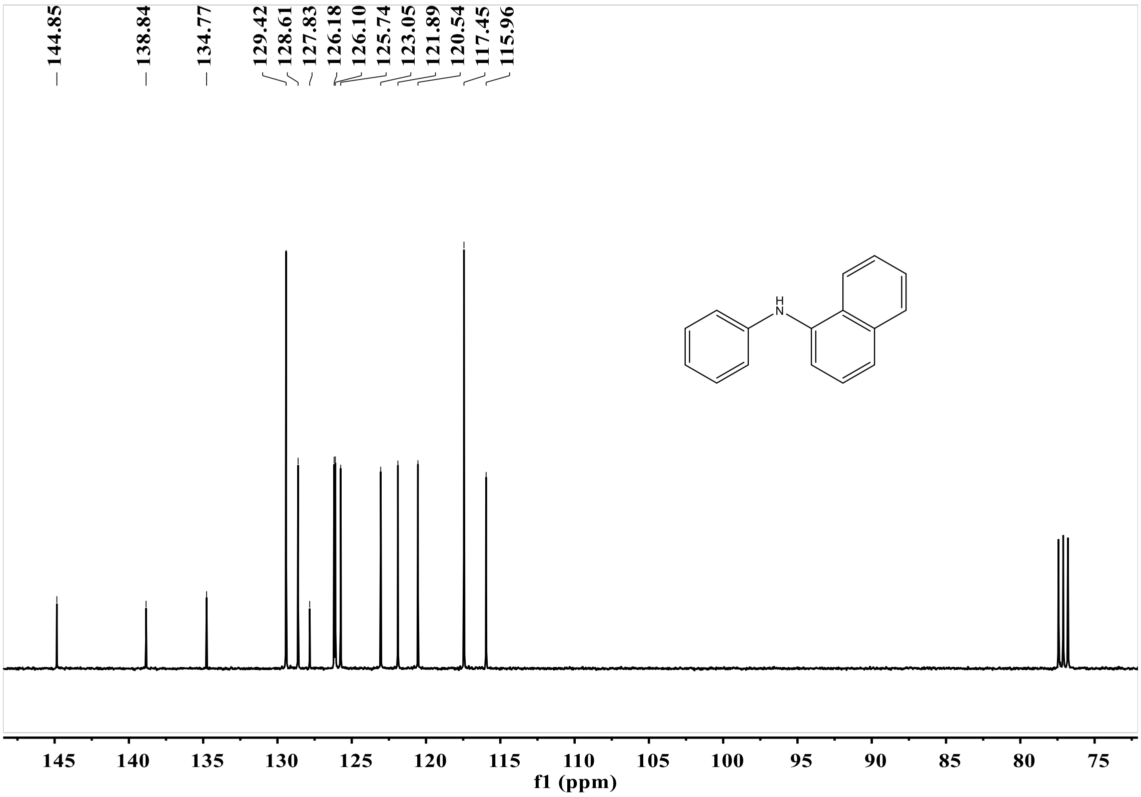

Figure S2. ^{13}C NMR spectrum of N-1 in $CDCl_3$.

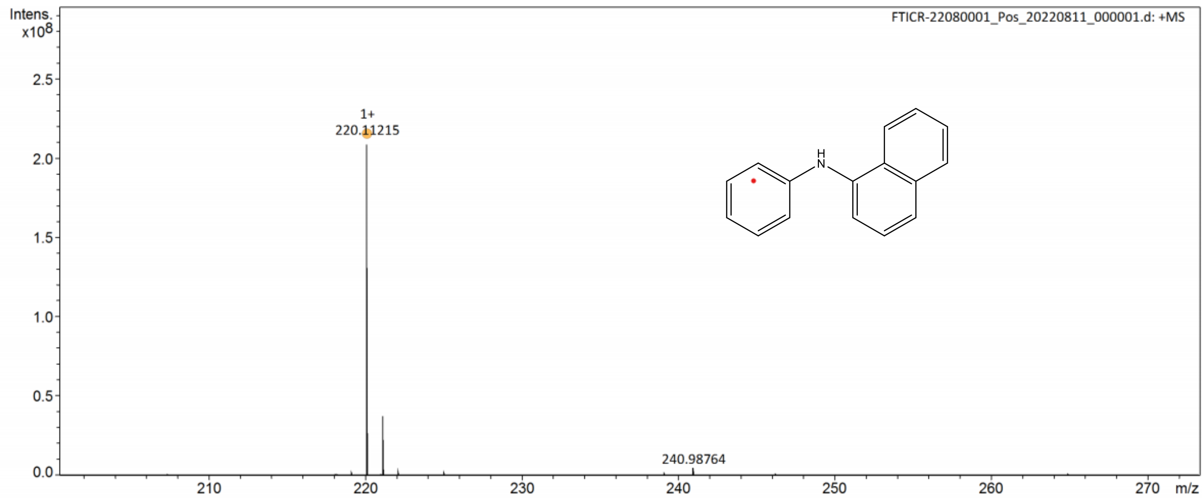

Meas. m/z	#	Ion Formula	Score	m/z	err [ppm]	Mean err [ppm]	mSigma	rdb	e⁻ Conf	N-Rule
220.112150	1	C16H14N	100.00	220.112076	-0.3	-0.3	2.1	11.0	even	ok

Figure S3. HR-MS spectrum of N-1.

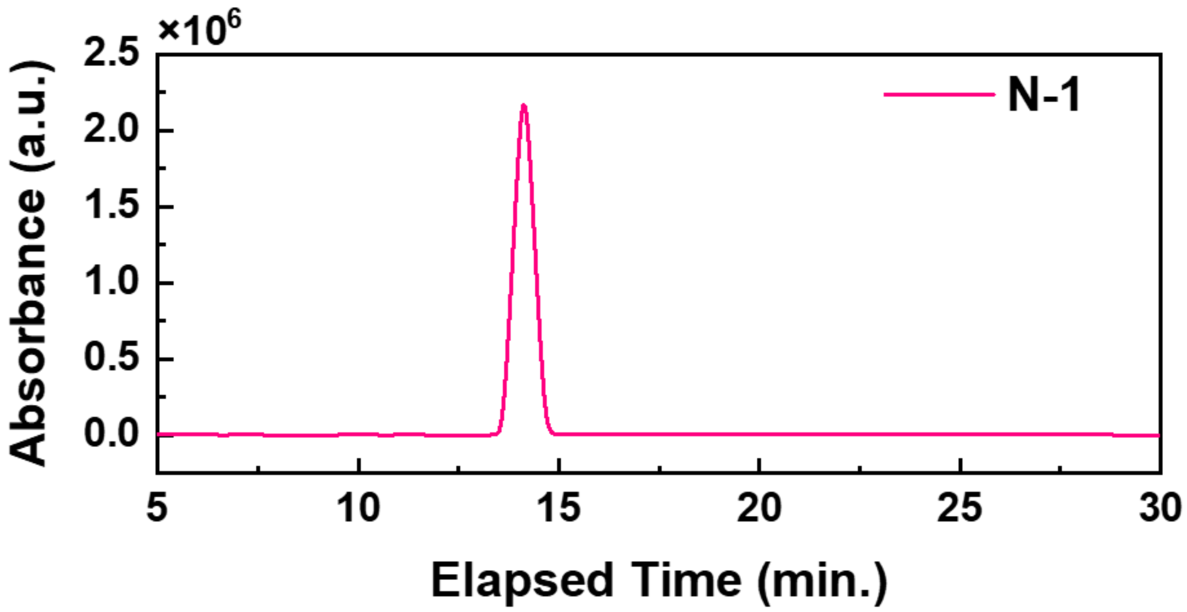

Figure S4. HPLC spectrum of N-1.

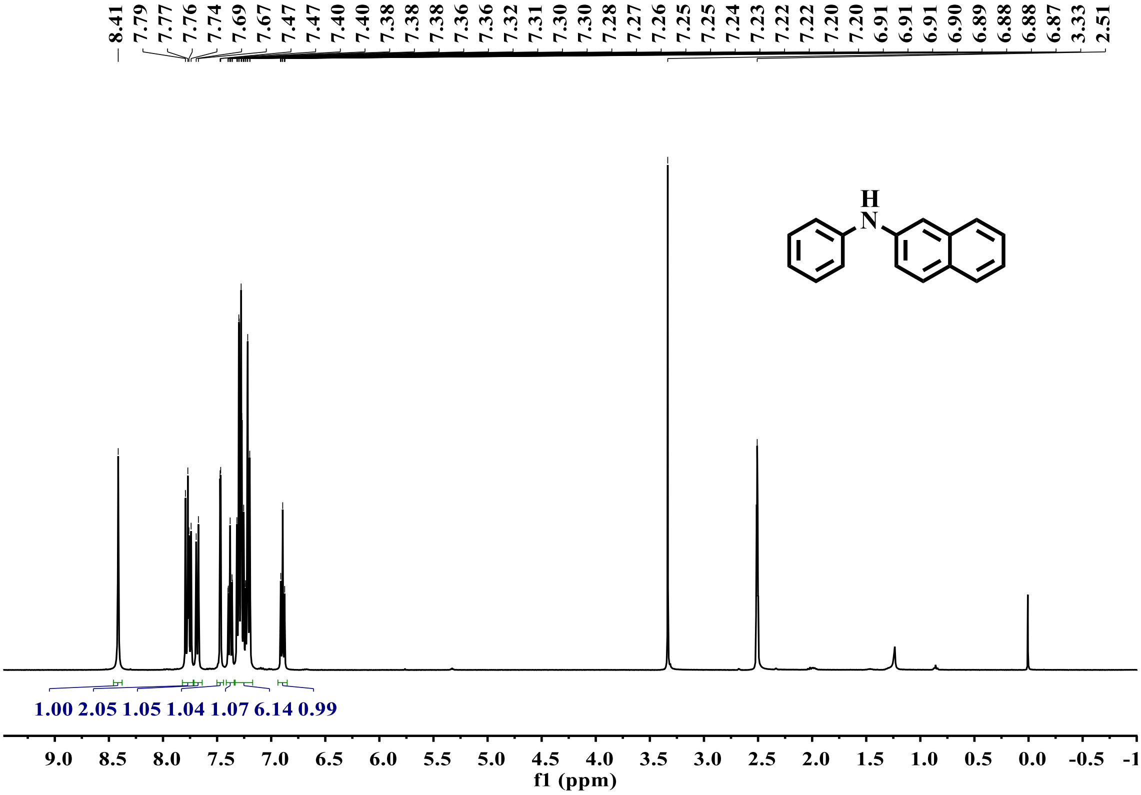

Figure S5. ^{1}H NMR spectrum of N-2 in DMSO-d_6.

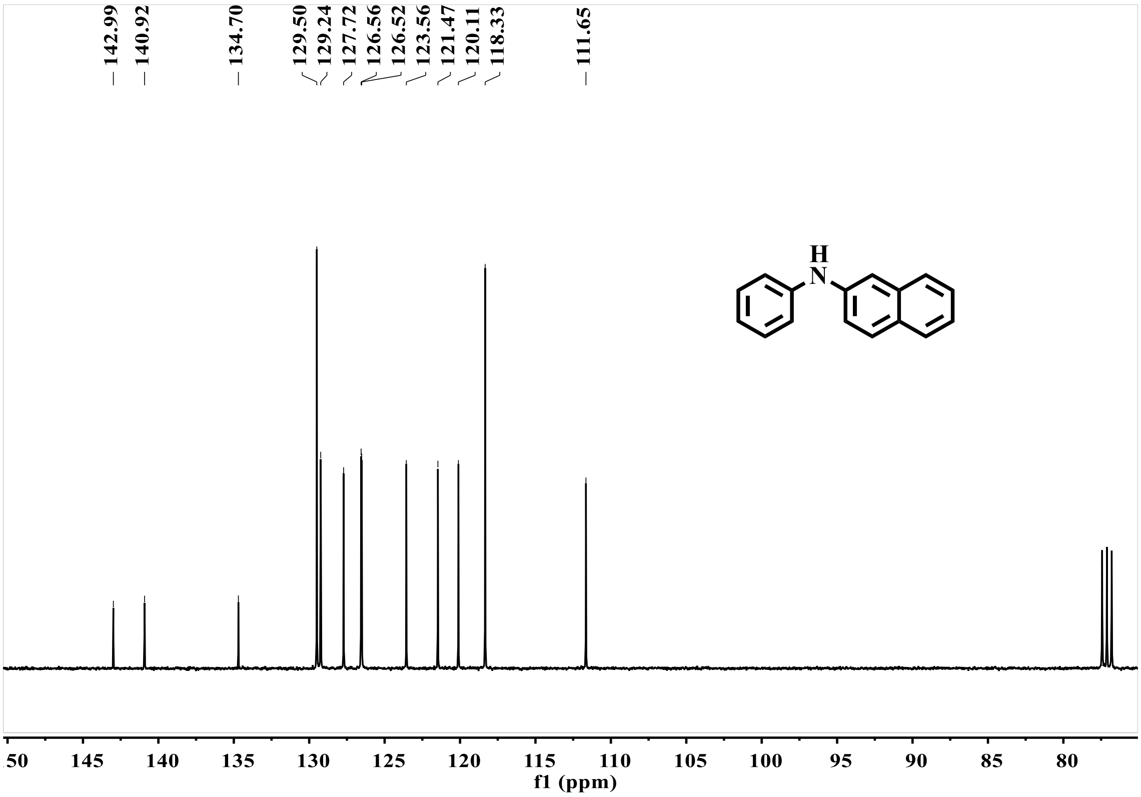

Figure S6. ^{13}C NMR spectrum of N-2 in $CDCl_3$.

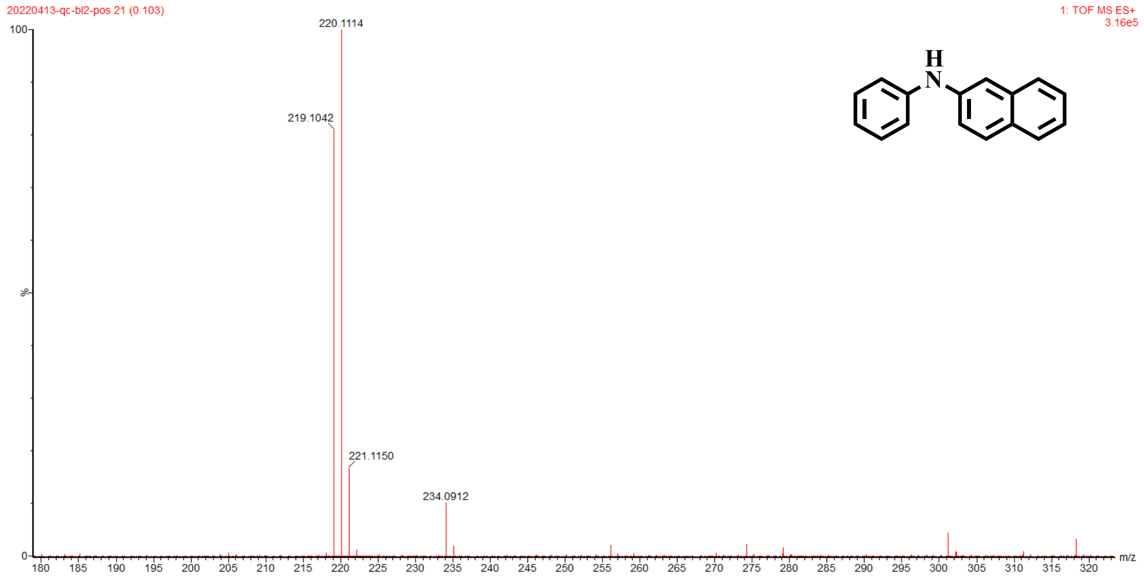

Figure S7. HR-MS spectrum of N-2.

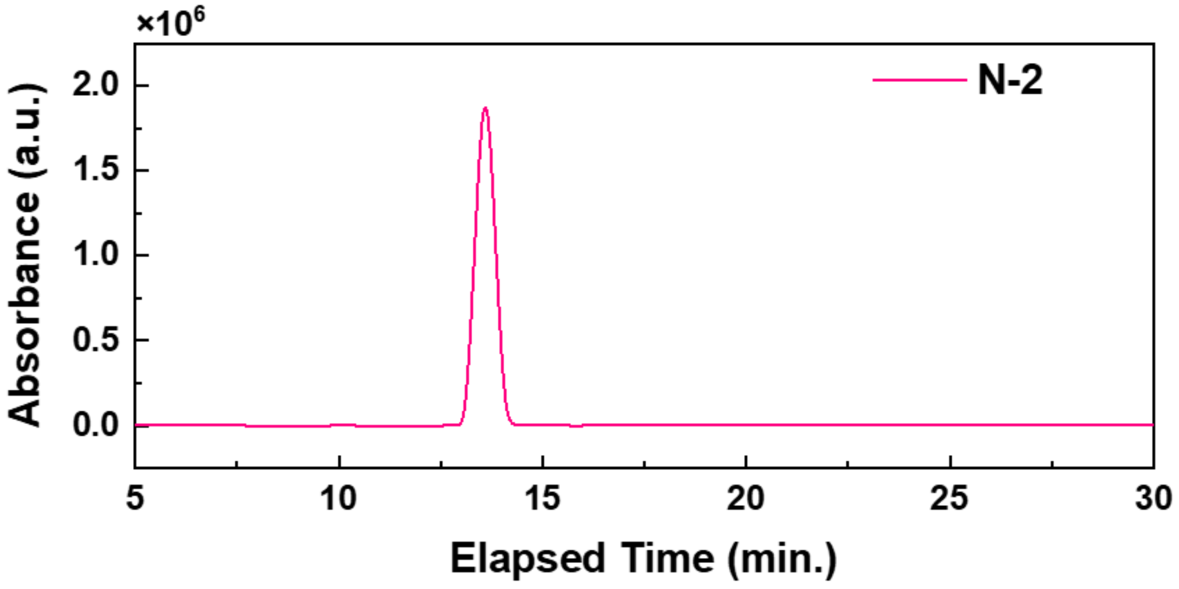

Figure S8. HPLC spectrum of N-2.

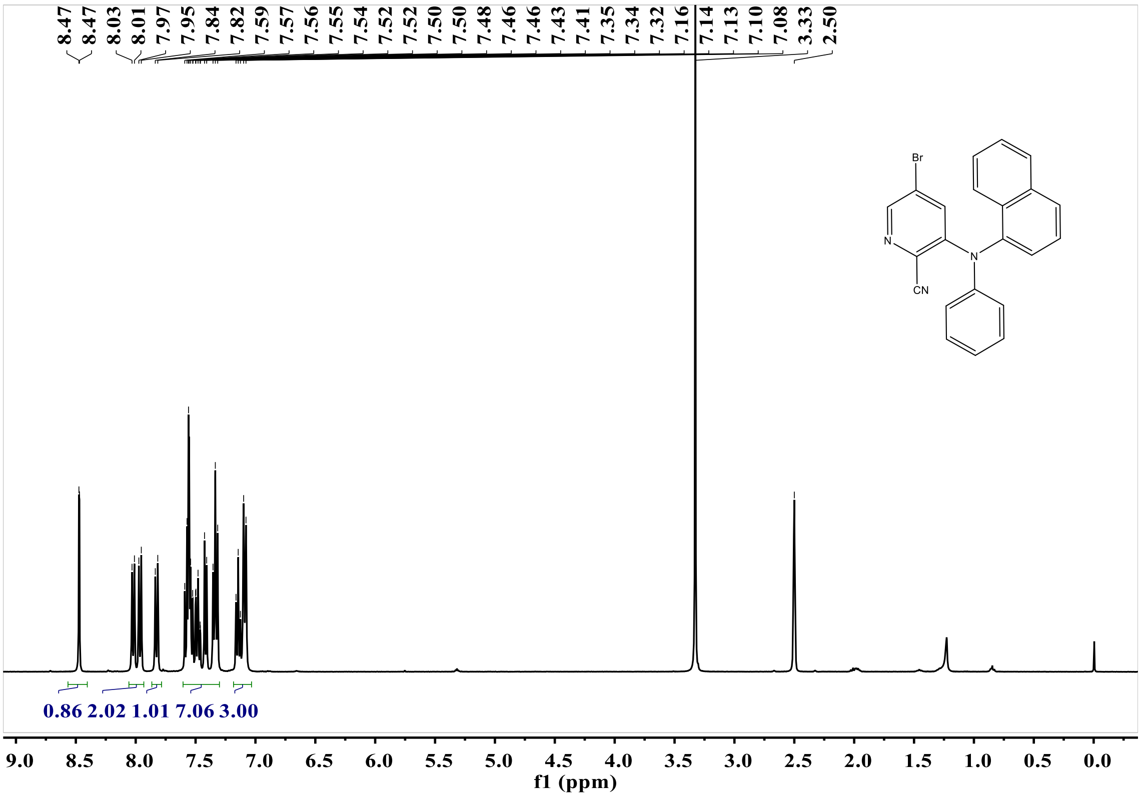

Figure S9. ^{1}H NMR spectrum of PNNA-1 in DMSO-d_6.

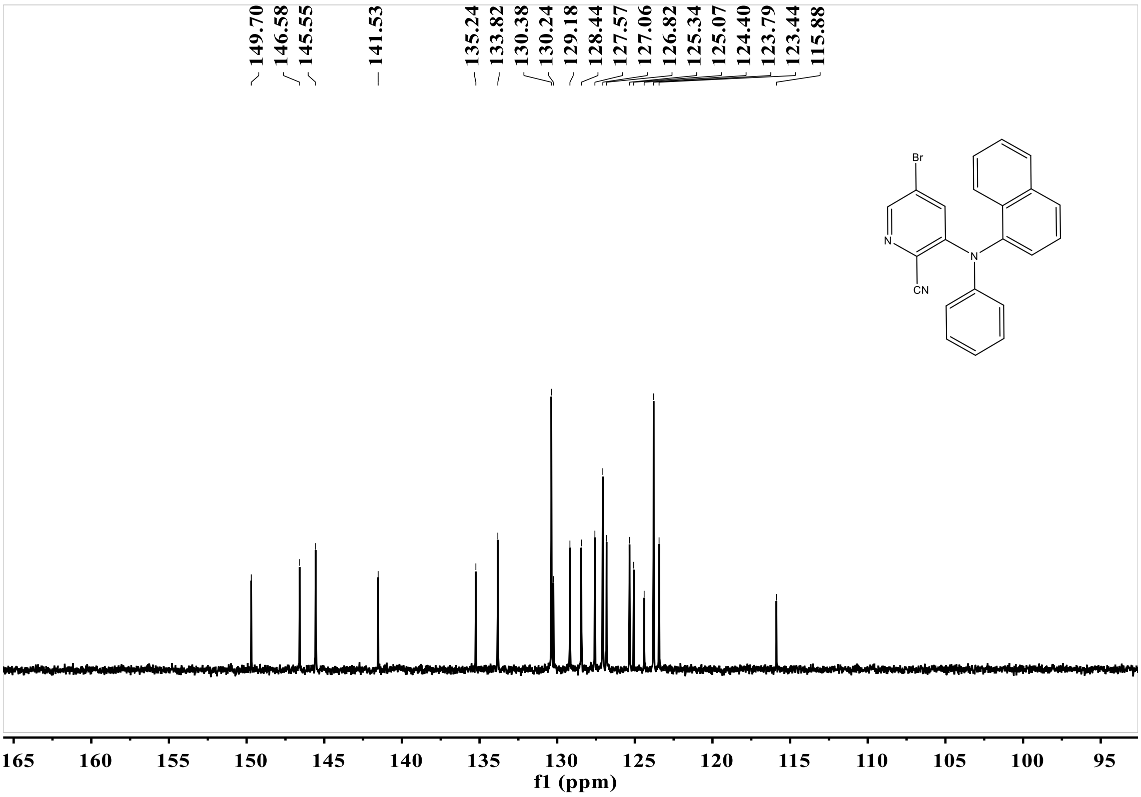

Figure S10. ^{13}C NMR spectrum of PNNA-1 in DMSO-d_6.

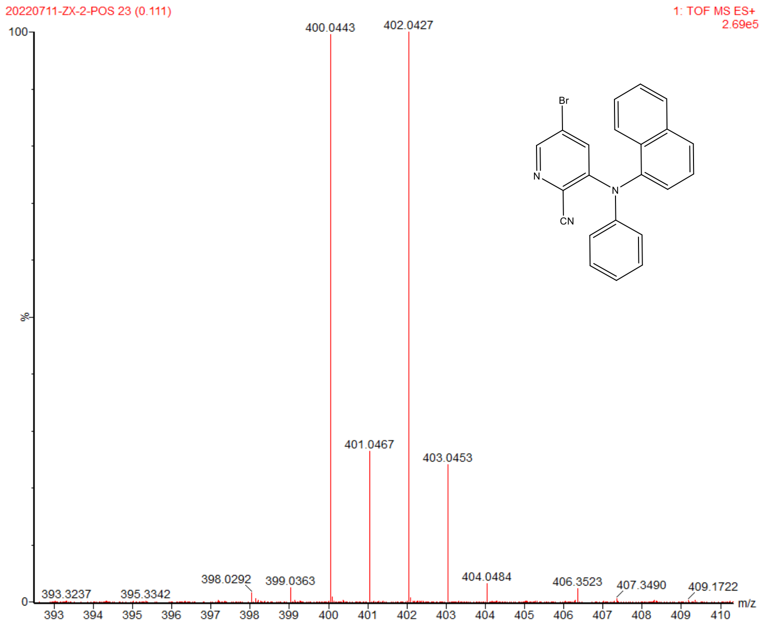

Figure S11. HR-MS spectrum of PNNA-1.

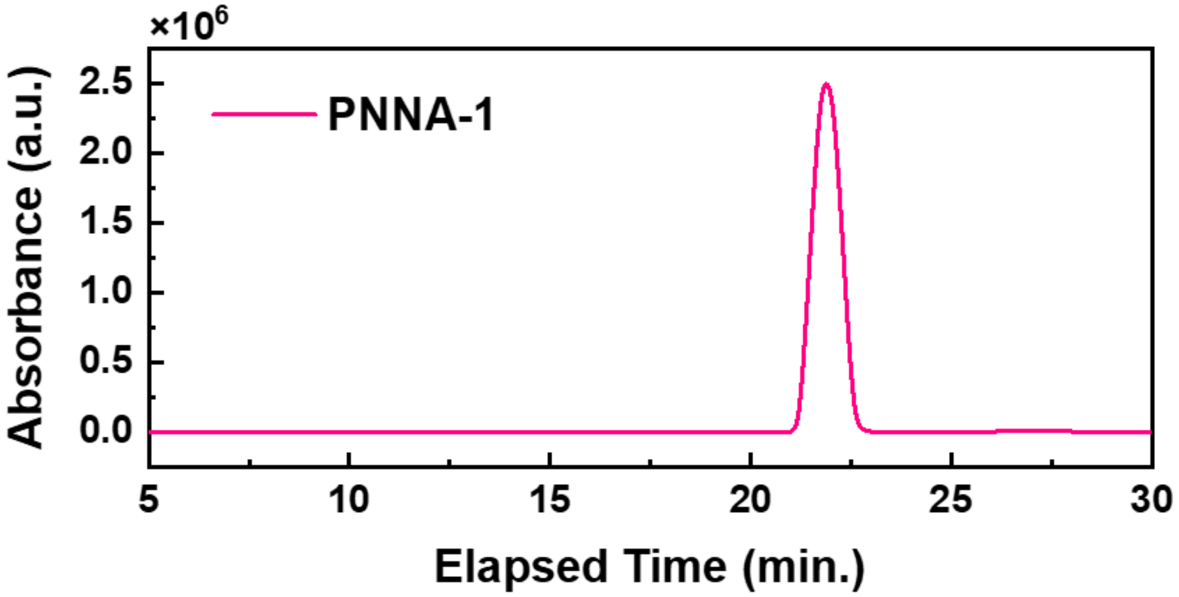

Figure S12. HPLC spectrum of PNNA-1.

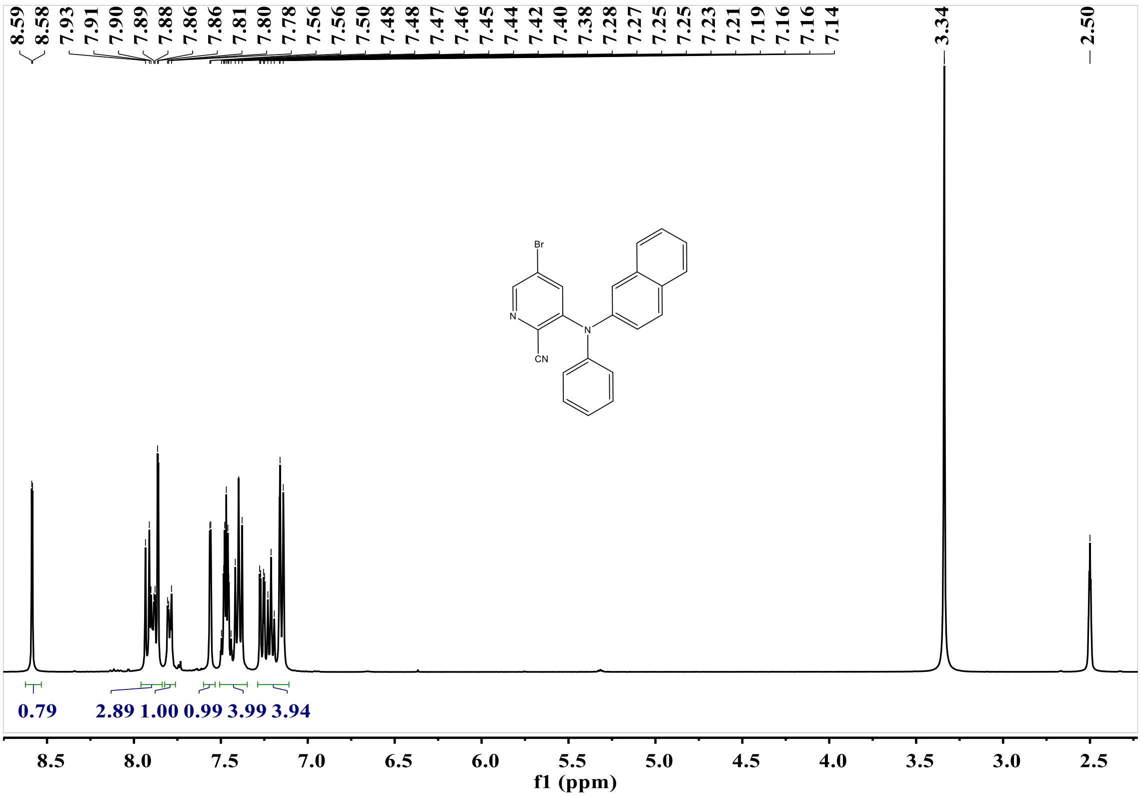

Figure S13. ^{1}H NMR spectrum of PNNA-2 in DMSO-d_6.

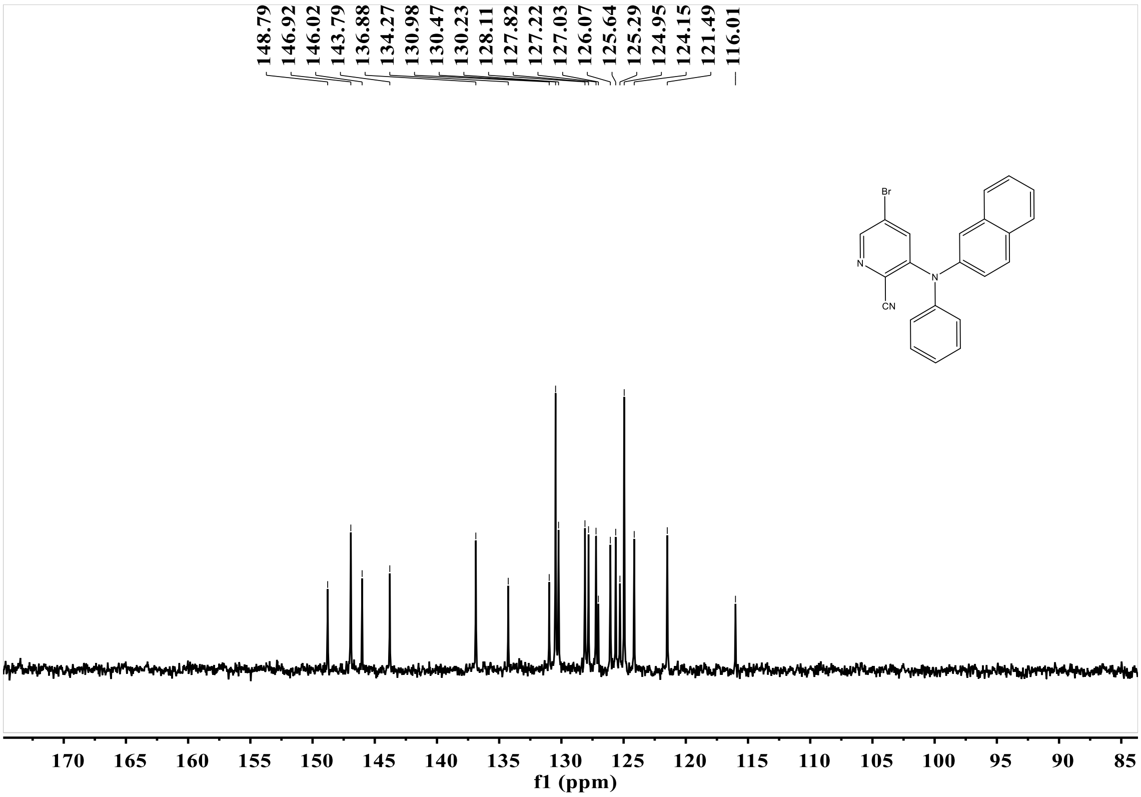

Figure S14. ^{13}C NMR spectrum of PNNA-2 in DMSO-d_6.

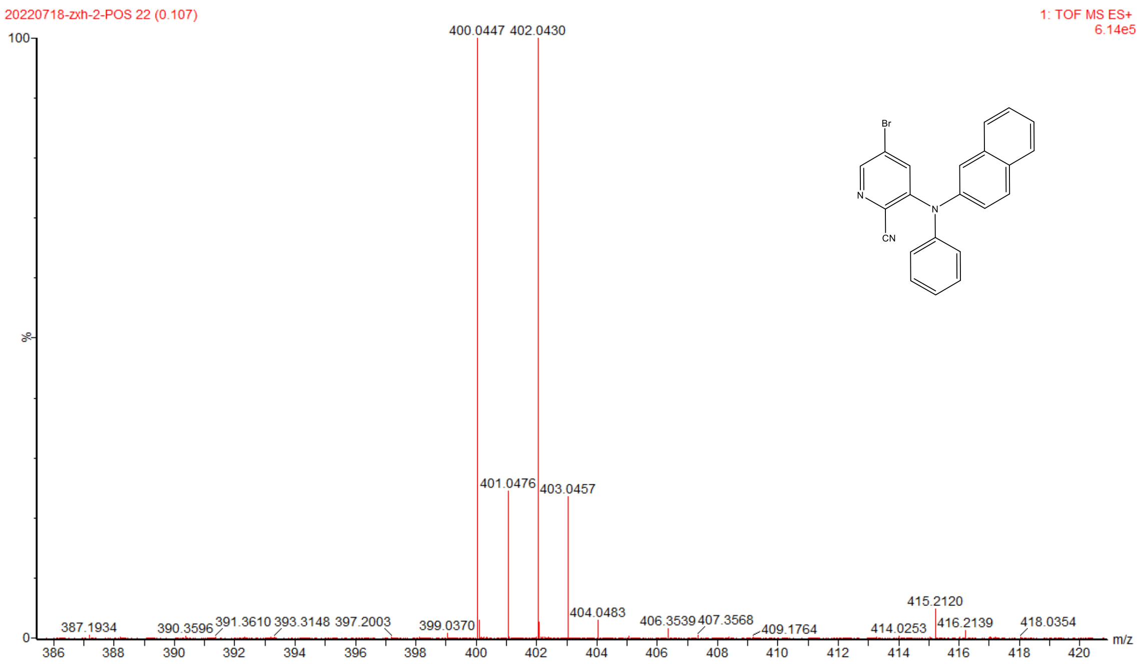

Figure S15. HR-MS spectrum of PNNA-2.

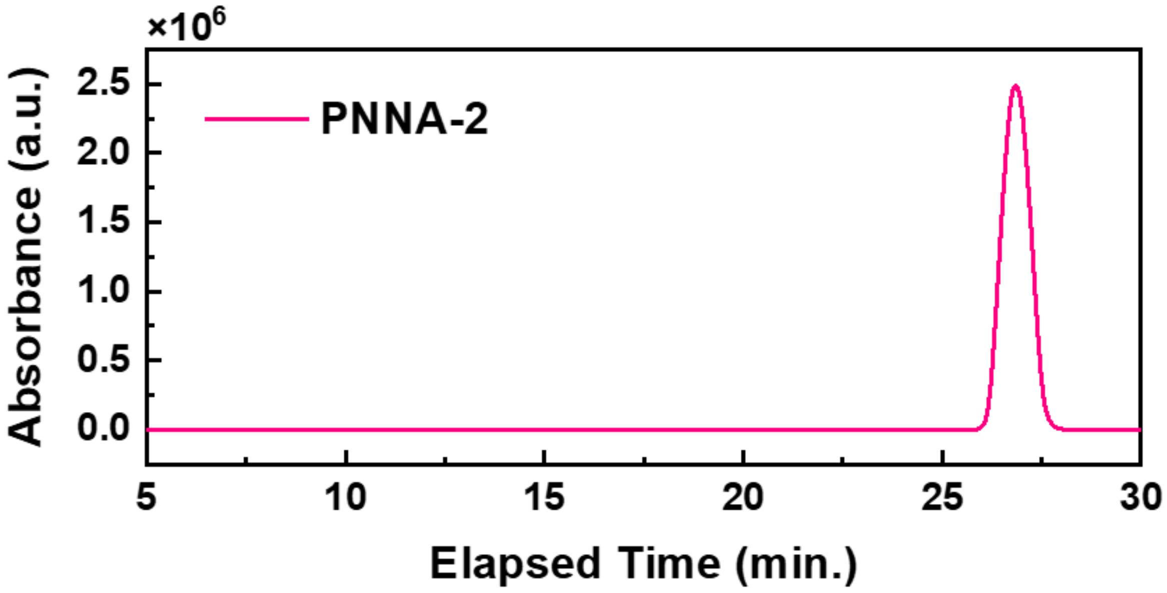

Figure S16. HPLC spectrum of PNNA-2.

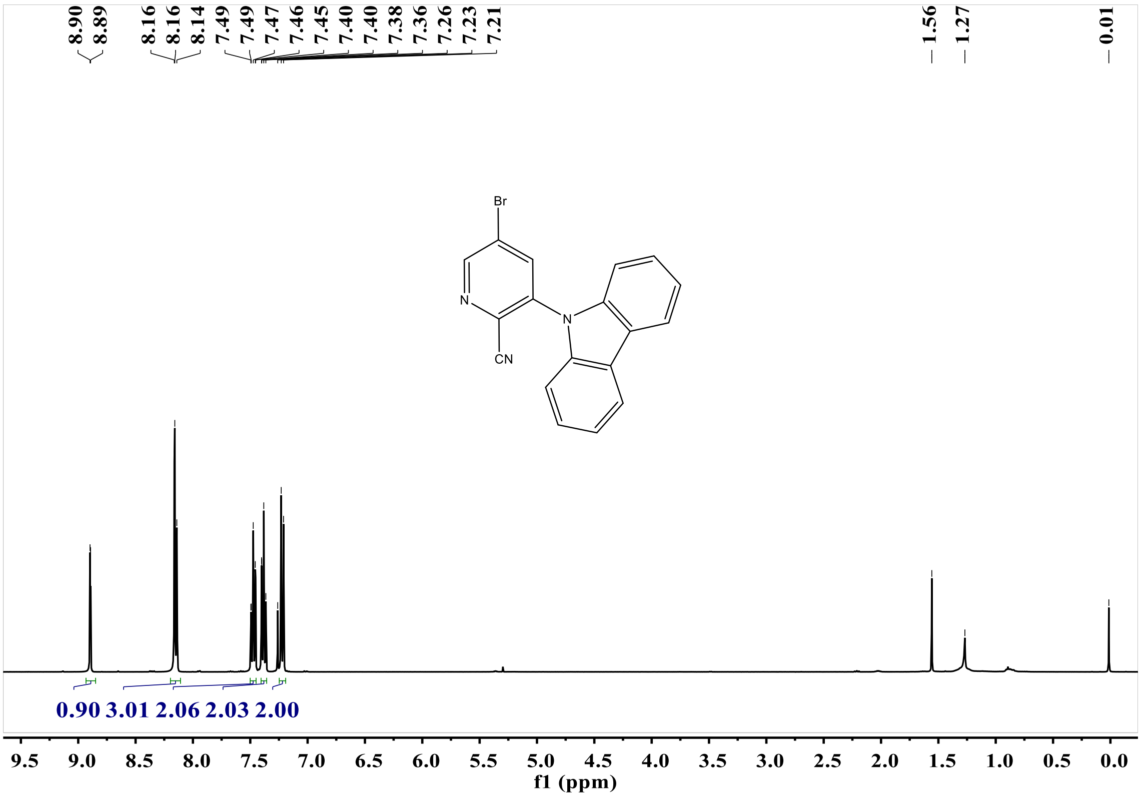

Figure S17. ^{1}H NMR spectrum of PNCz in $CDCl_3$.

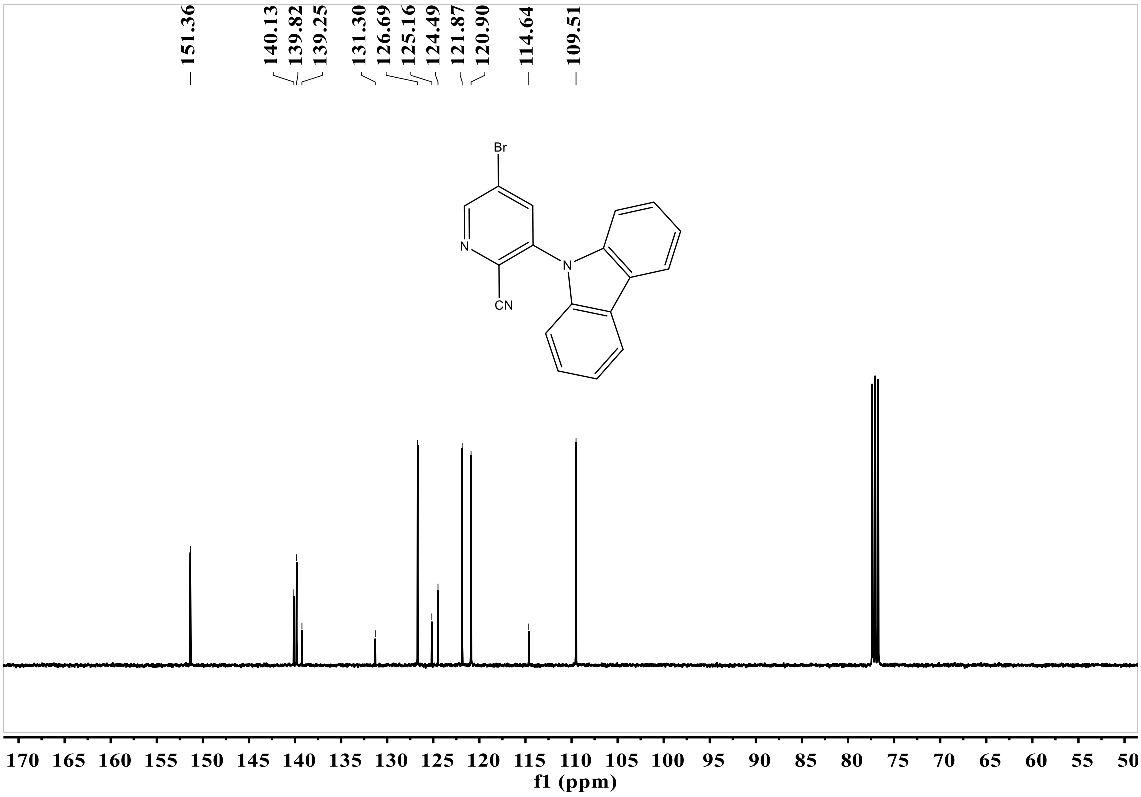

Figure S18. ^{13}C NMR spectrum of PNCz in $CDCl_3$.

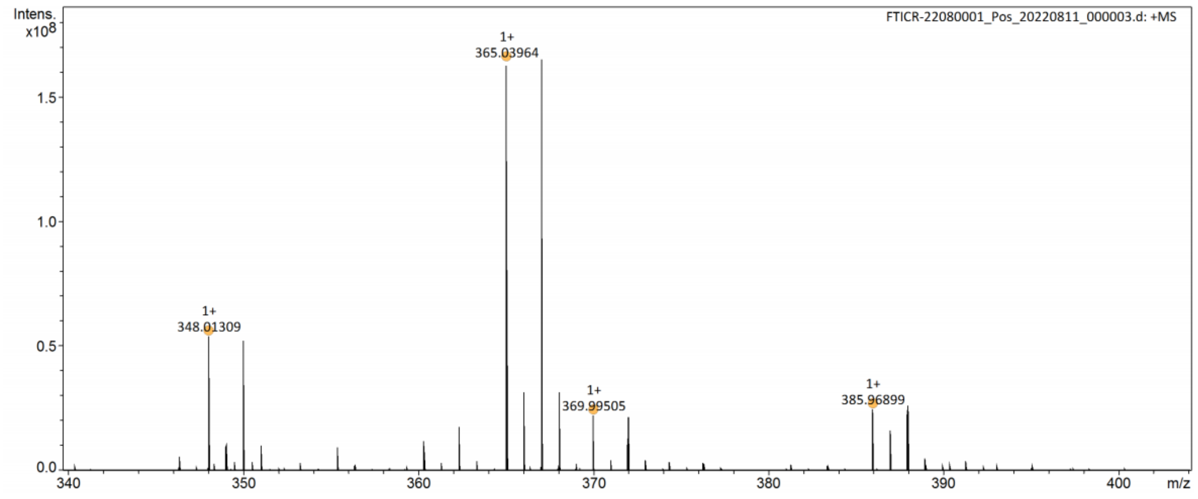

Meas. m/z	#	Ion Formula	Score	m/z	err [ppm]	Mean err [ppm]	mSigma	rdb	e⁻ Conf	N-Rule
348.013091	1	C18H11BrN3	100.00	348.013086	-0.0	-0.0	8.7	17.0	even	ok
365.039645	1	C18H14BrN4	100.00	365.039635	-0.0	0.0	13.1	16.0	even	ok
369.995046	1	C18H10BrN3Na	100.00	369.995031	-0.0	-0.1	8.2	17.0	even	ok
385.968989	1	C18H10BrKN3	100.00	385.968968	-0.1	-0.3	11.4	17.0	even	ok

Figure S19. HR-MS spectrum of PNCz.

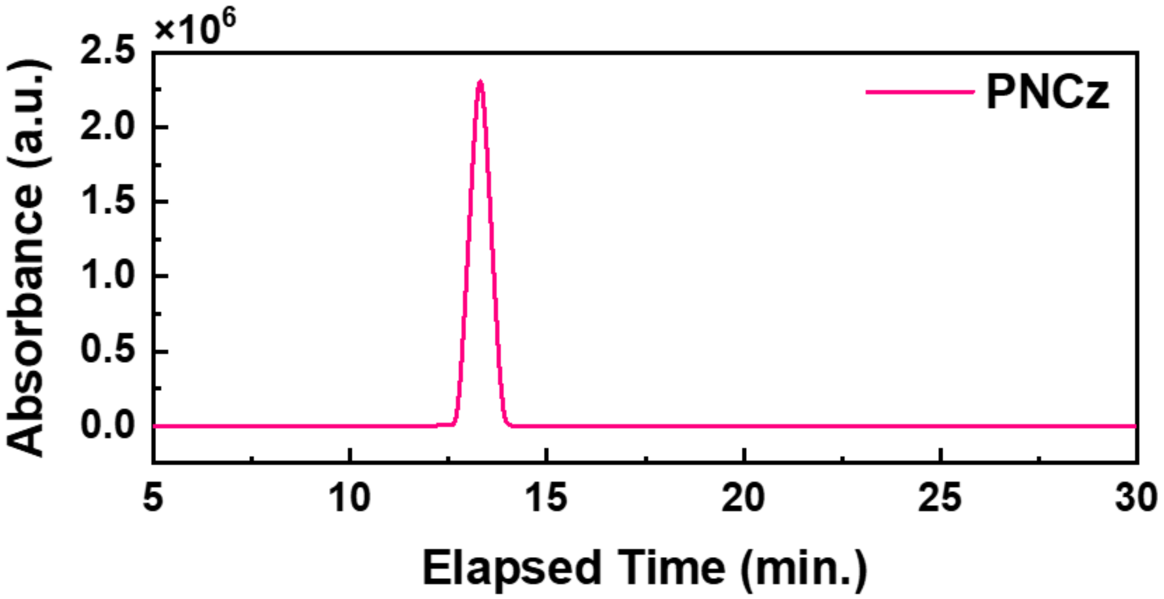

Figure S20. HPLC spectrum of PNCz.

4. **Photophysical properties in solution**

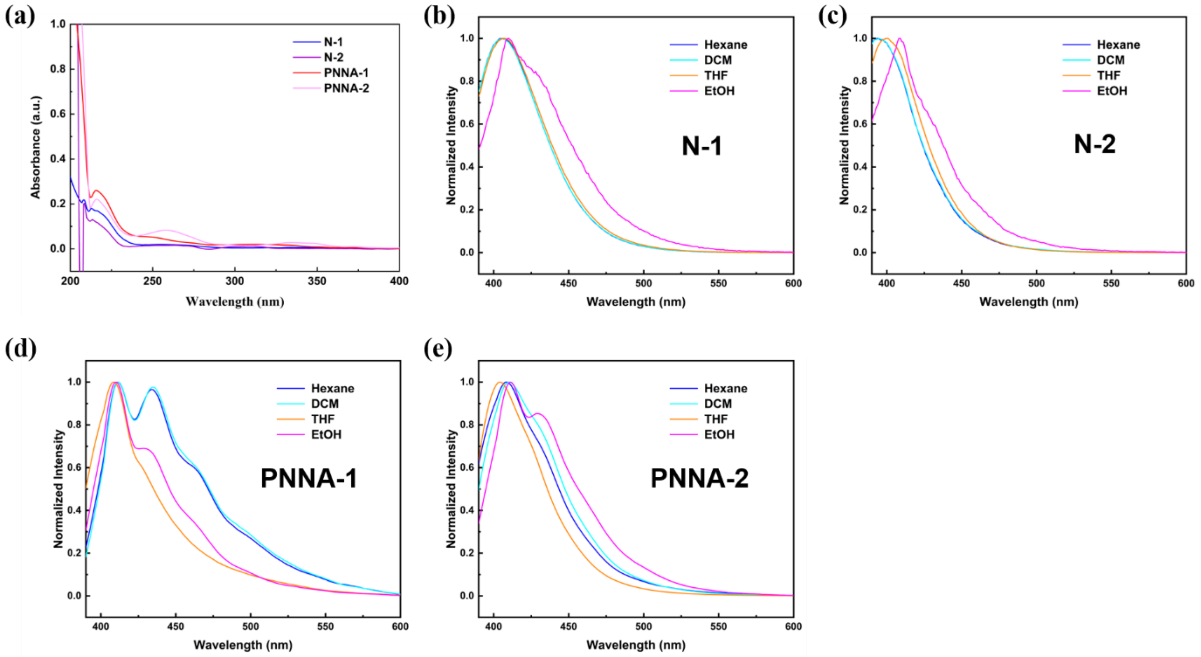

Figure S21. (a) Absorption spectra; and PL spectra of (b) N-1; (c) N-2; (d) PNNA-1; and (e) PNNA-2 in different solutions at ambient condition. (20 μM, λ_{ex} = 365 nm)

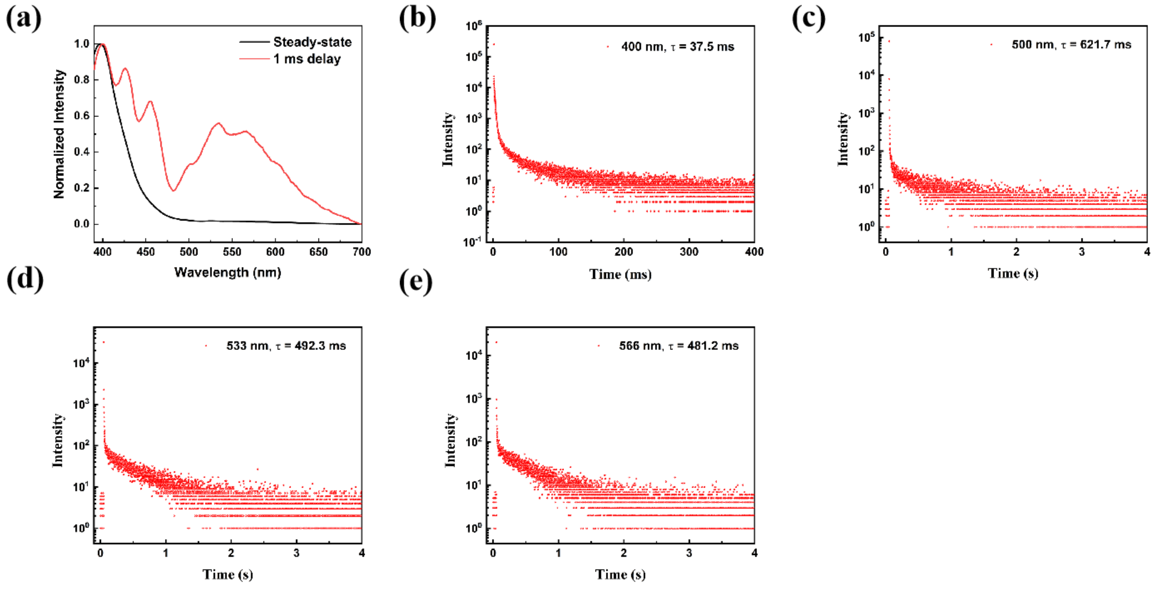

Figure S22. (a) Steady-state and delayed PL spectra; and (b-e) decay spectra of N-1 in dilute toluene solution at 77 K. (20 μM, λ_{ex} = 365 nm)

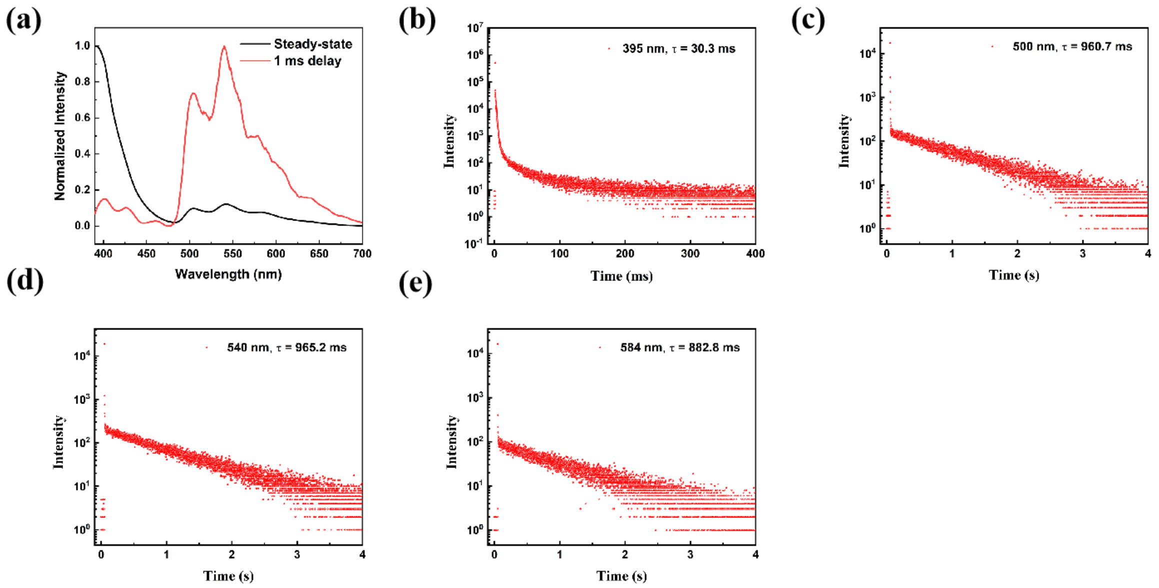

Figure S23. (a) Steady-state and delayed PL spectra; and (b-e) decay spectra of N-2 in dilute toluene solution at 77 K. (20 μM, λ_{ex} = 365 nm)

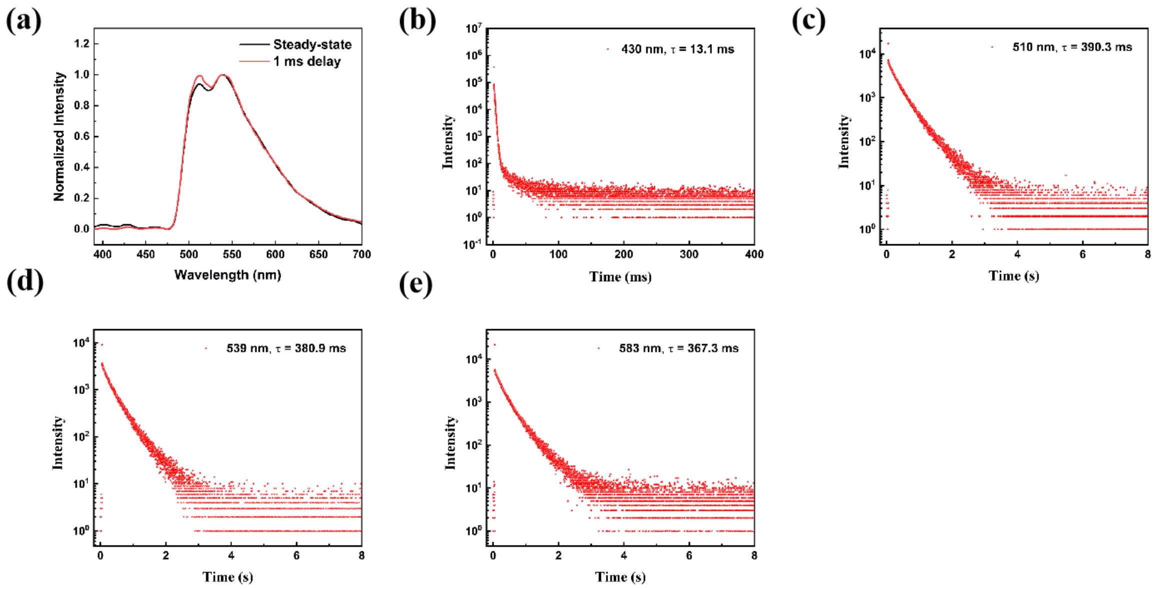

Figure S24. (a) Steady-state and delayed PL spectra; and (b-e) decay spectra of PNNA-1 in dilute toluene solution at 77 K. (20 μM, λ_{ex} = 365 nm)

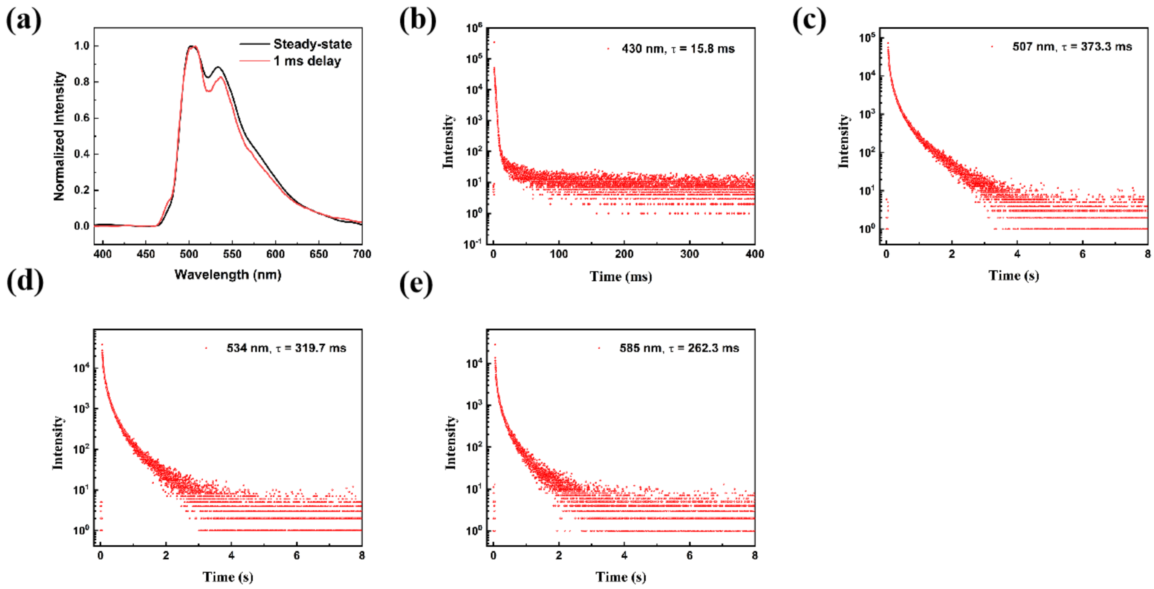

Figure S25. (a) Steady-state and delayed PL spectra; and (b-e) decay spectra of PNNA-2 in dilute toluene solution at 77 K. (20 μM, λ_{ex} = 365 nm)

5. Photophysical properties in solid state

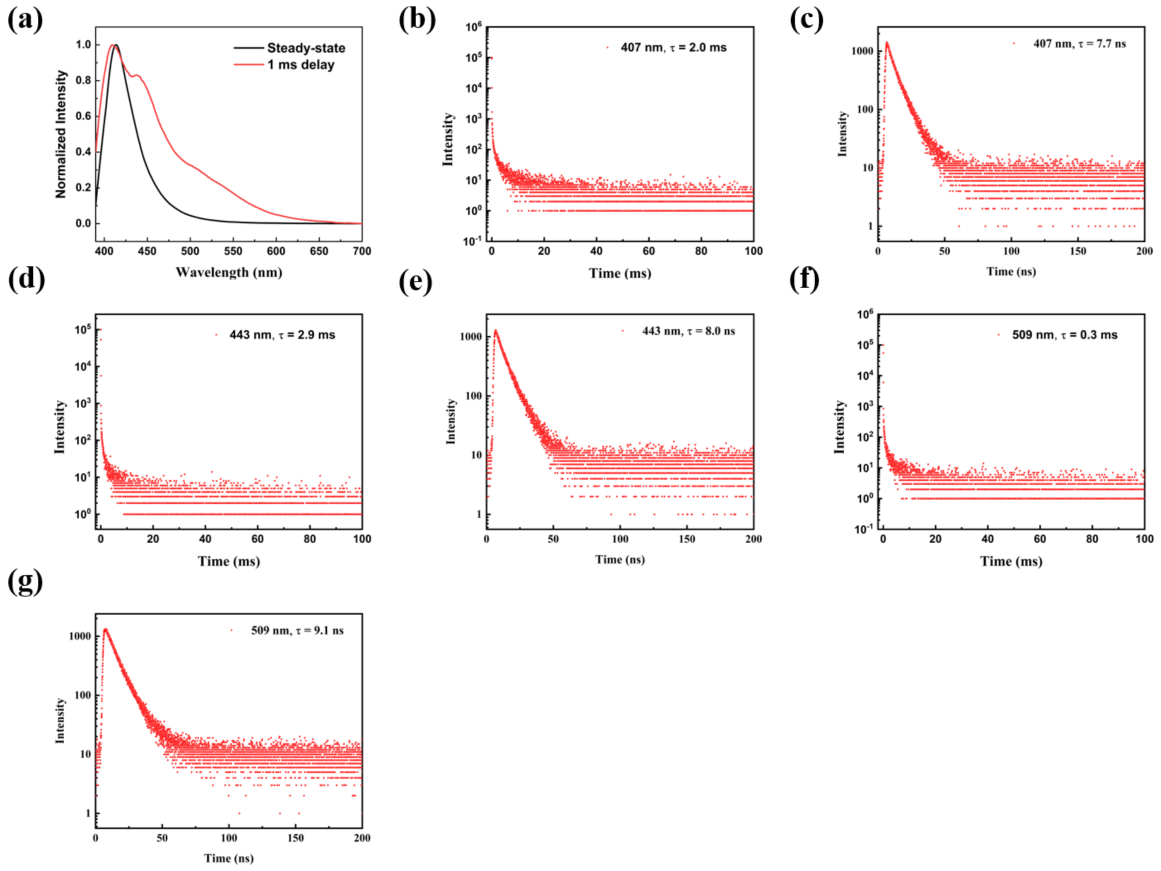

Figure S26. (a) PL spectra of the N-1 powder; and (b-g) decay spectra at ambient condition. (λ_{ex} = 365 nm)

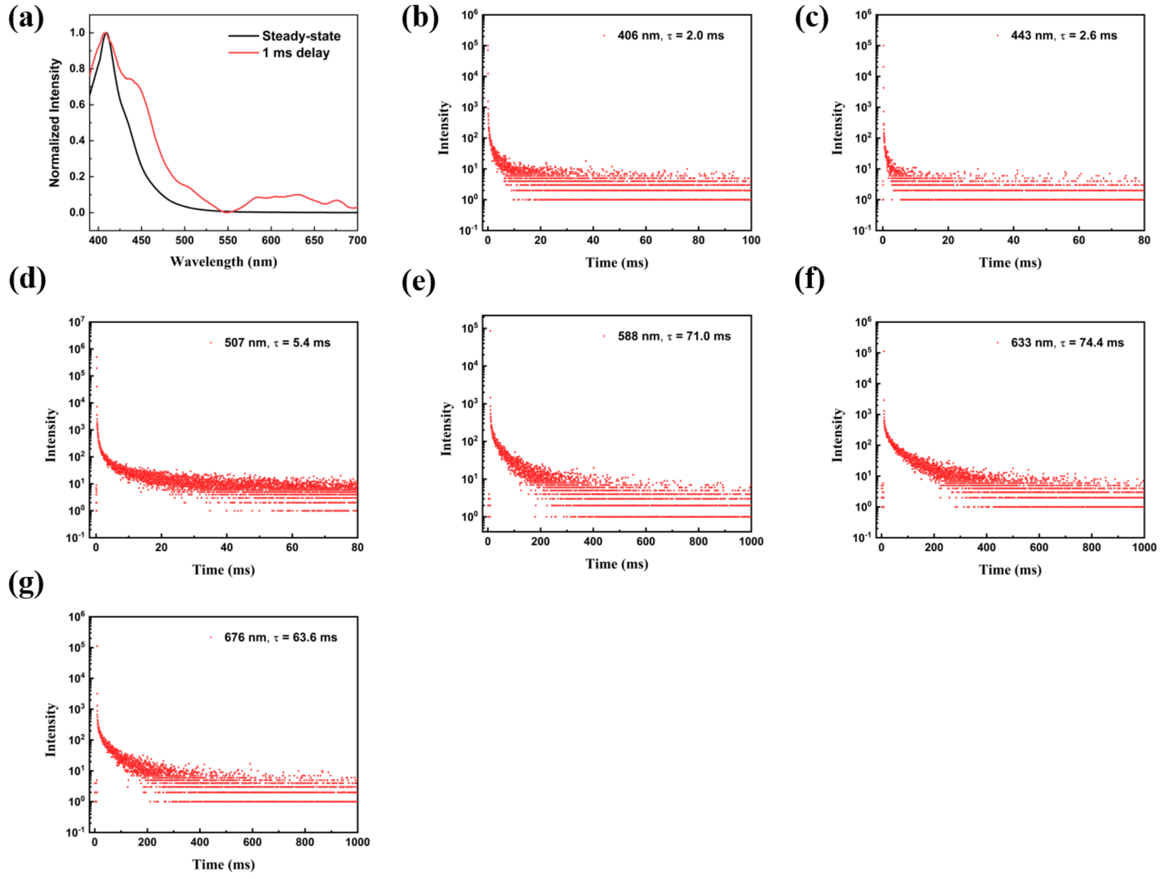

Figure S27. (a) PL spectra of the N-1 powder; and (b-g) decay spectra at 77 K. (λ_{ex} = 365 nm)

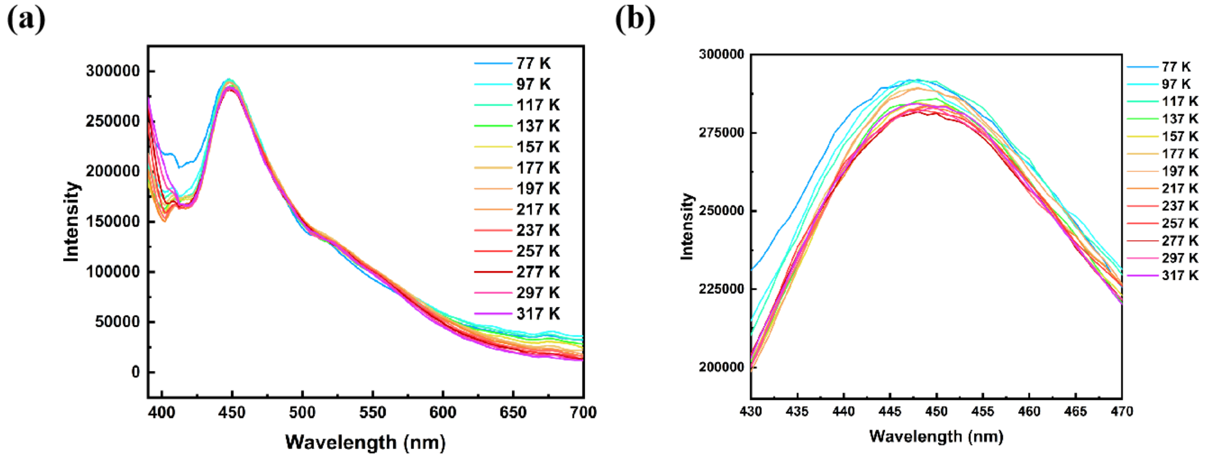

Figure S28. (a) Variable-temperature steady-state PL spectra; and (b) partial enlarged spectra of the N-1 powder. (λ_{ex} = 365 nm)

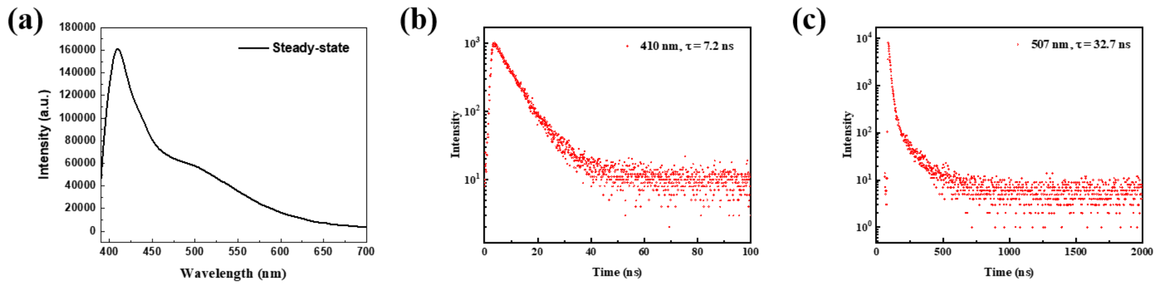

Figure S29. (a) PL spectrum of the N-2 powder; and (b, c) decay spectra at ambient condition. (λ_{ex} = 365 nm)

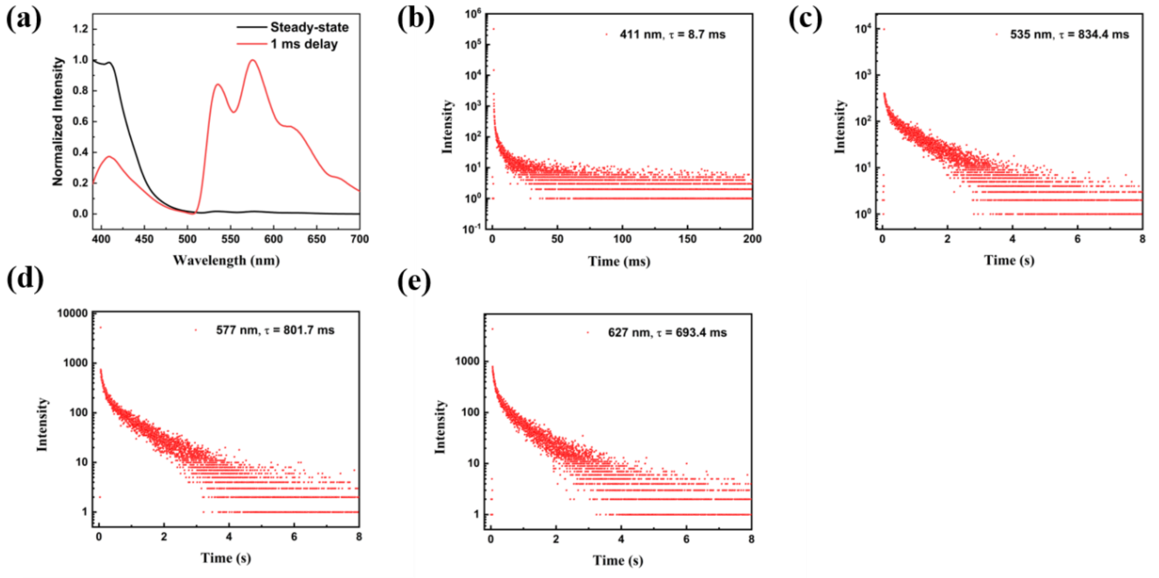

Figure S30. (a) PL spectra of the N-2 powder; and (b-e) decay spectra at 77 K. (λ_{ex} = 365 nm)

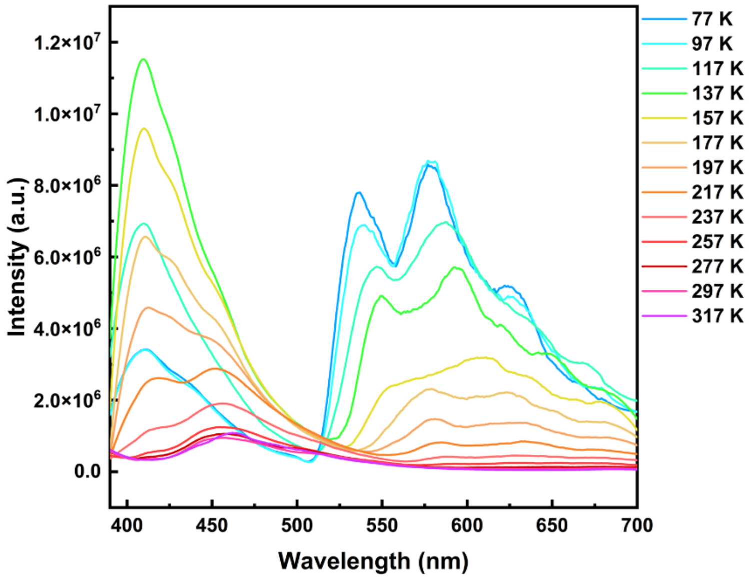

Figure S31. Variable-temperature delayed PL spectra of the N-2 powder. (λ_{ex} = 365 nm)

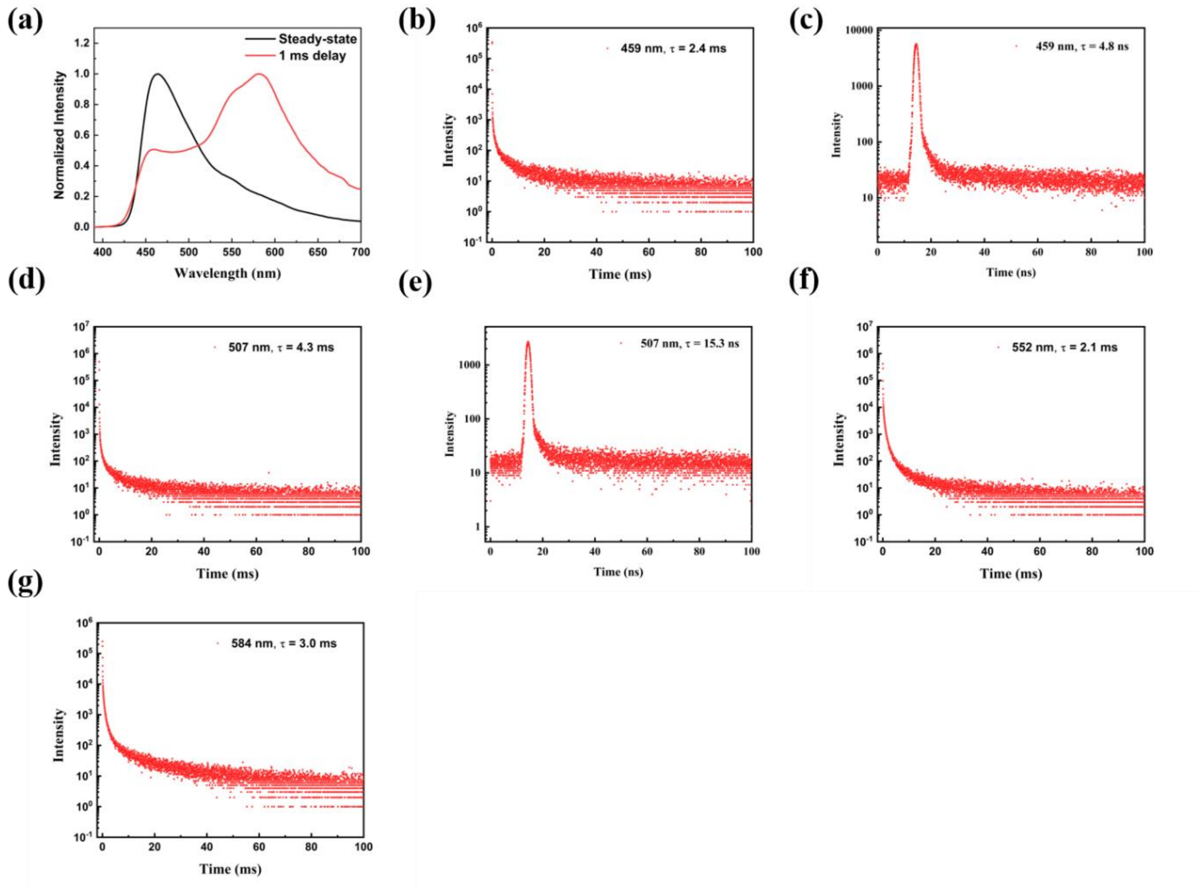

Figure S32. (a) PL spectra of the PNNA-1 powder; and (b-g) decay spectra at ambient condition. (λ_{ex} = 365 nm)

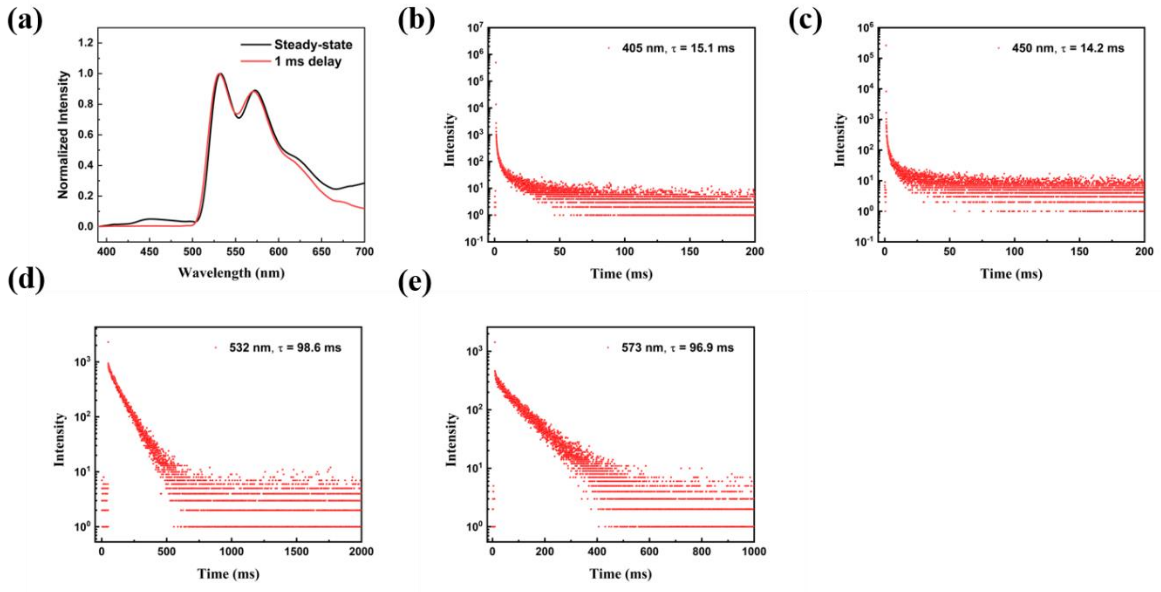

Figure S33. (a) PL spectra of the PNNA-1 powder; and (b-e) decay spectra at 77 K. (λ_{ex} = 365 nm)

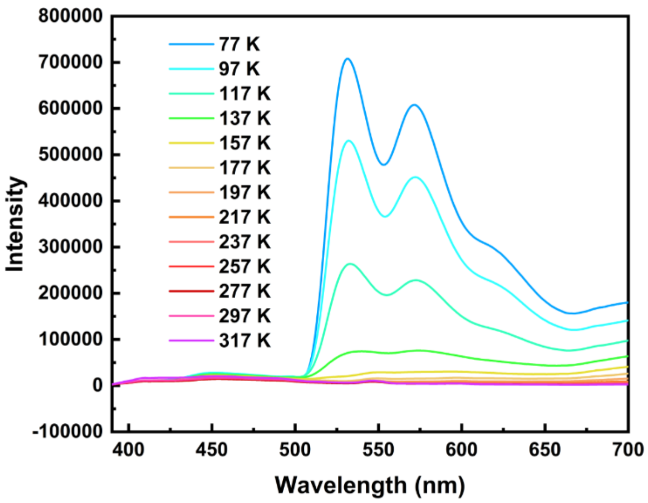

Figure S34. Variable-temperature delayed PL spectra of the PNNA-1 powder. (λ_{ex} = 365 nm)

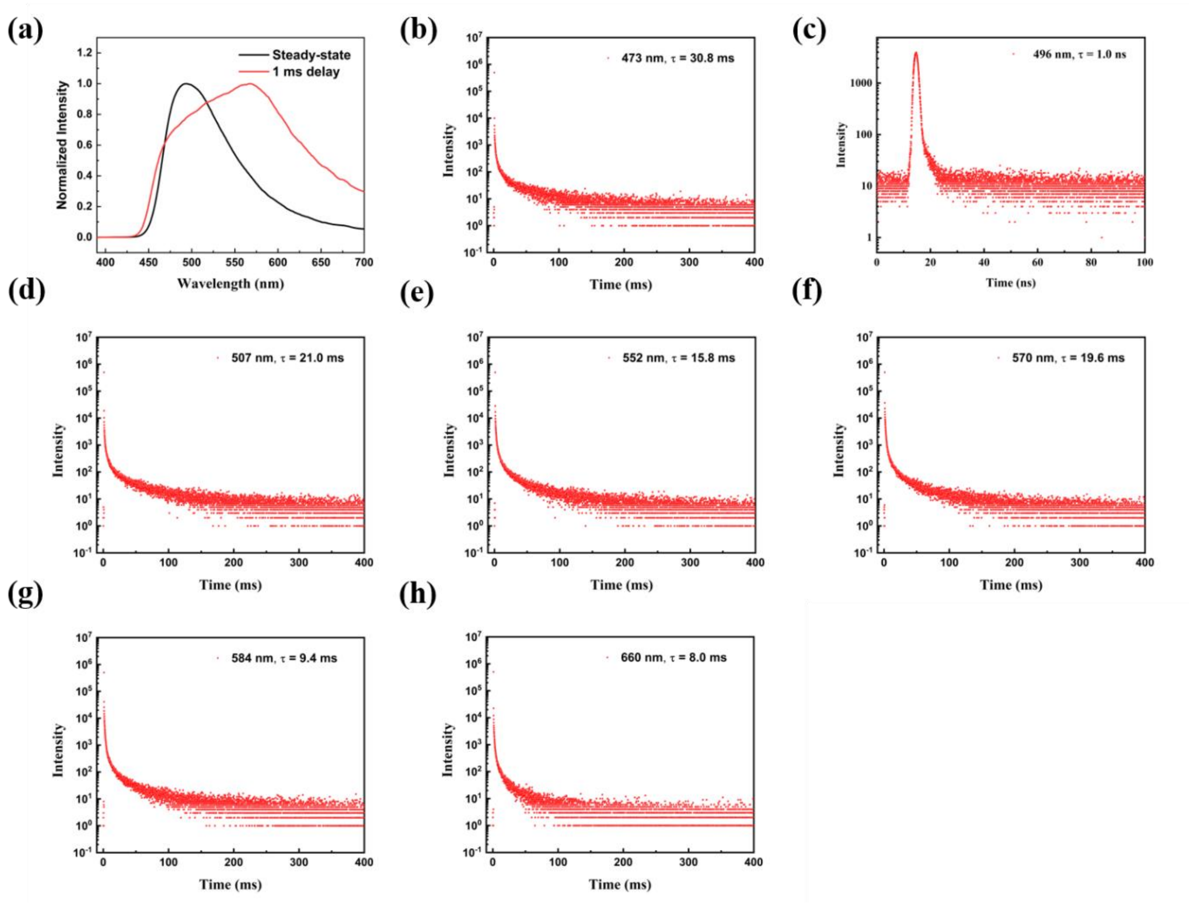

Figure S35. (a) PL spectra of the PNNA-2 powder; and (b-h) decay spectra at ambient condition. (λ_{ex} = 365 nm)

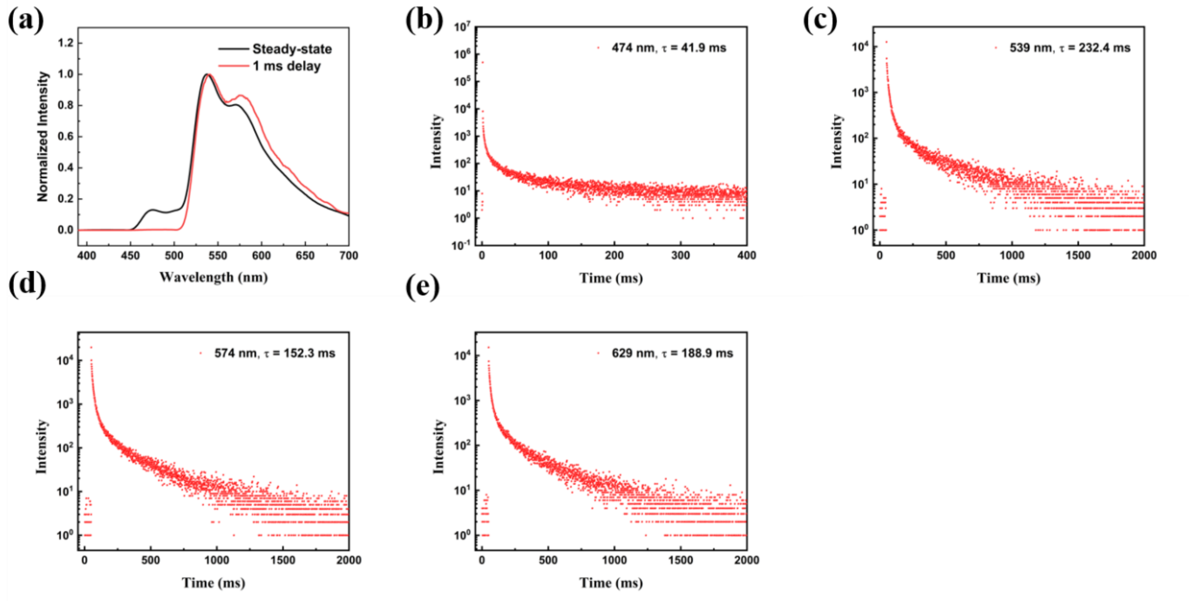

Figure S36. (a) PL spectra of the PNNA-2 powder; and (b-e) decay spectra at 77 K. (λ_{ex} = 365 nm)

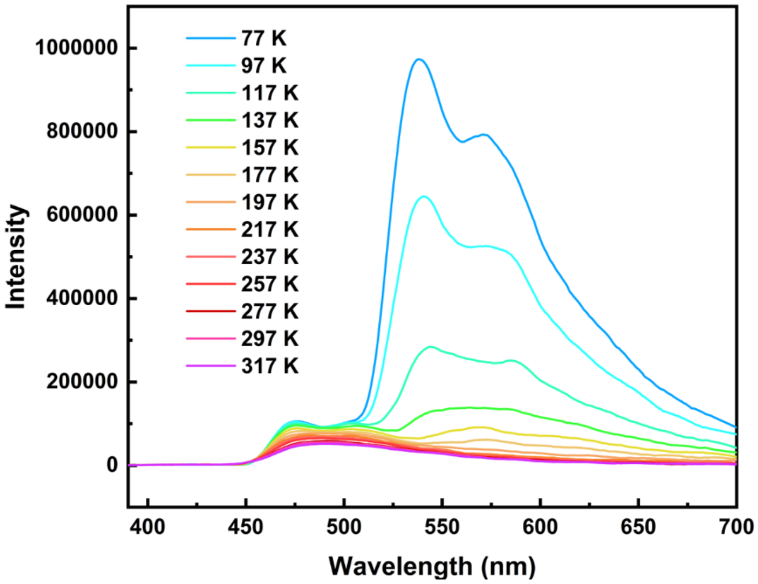

Figure S37. Variable-temperature delayed PL spectra of the PNNA-2 powder. (λ_{ex} = 365 nm)

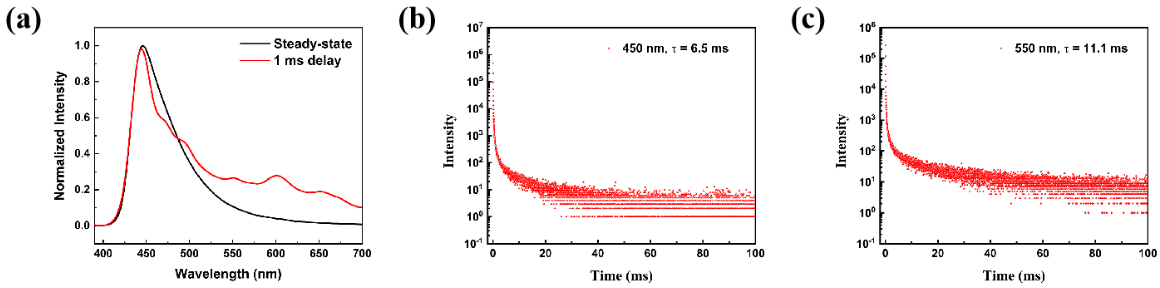

Figure S38. (a) PL spectra of the PNCz powder; and (b, c) decay spectra at ambient condition. (λ_{ex} = 365 nm)

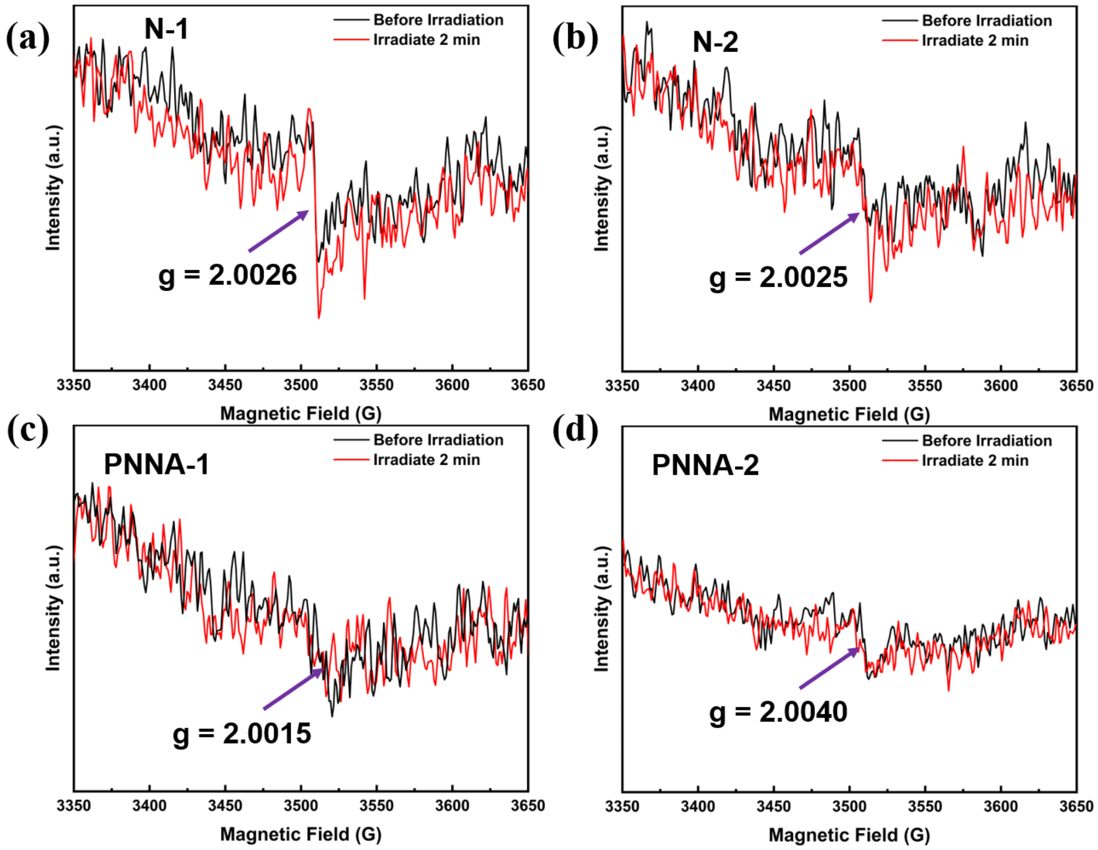

Figure S39. ESR spectra of (a) the N-1 powde; (b) the N-2 powder; (c) the PNNA-1 powder and; (d) the PNNA-2 powder before irradiation and after irradiation at ambient condition. (λ_{ex} = 365 nm)

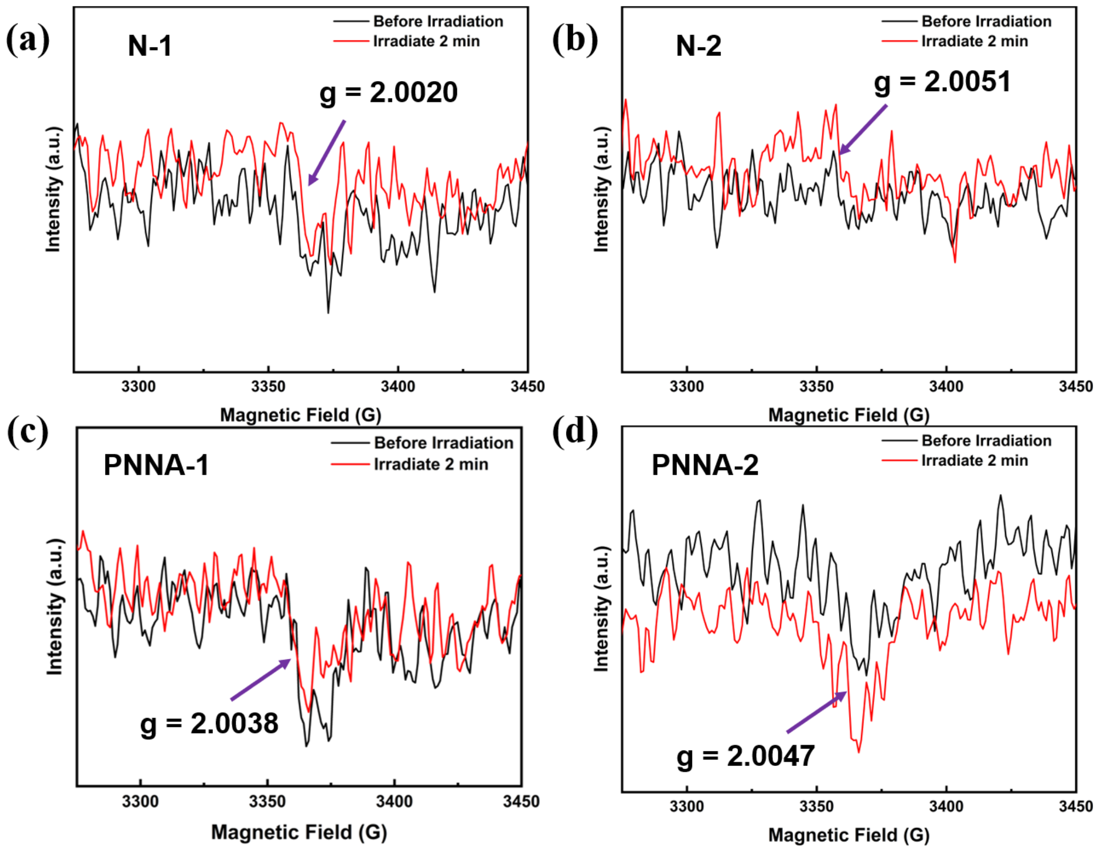

Figure S40. ESR spectra of (a) the N-1 powder; (b) the N-2 powder; (c) the PNNA-1 powder; and (d) the PNNA-2 powder before irradiation and after irradiation at 100 K. (λ_{ex} = 365 nm)

6. **Photophysical properties in PMMA film**.

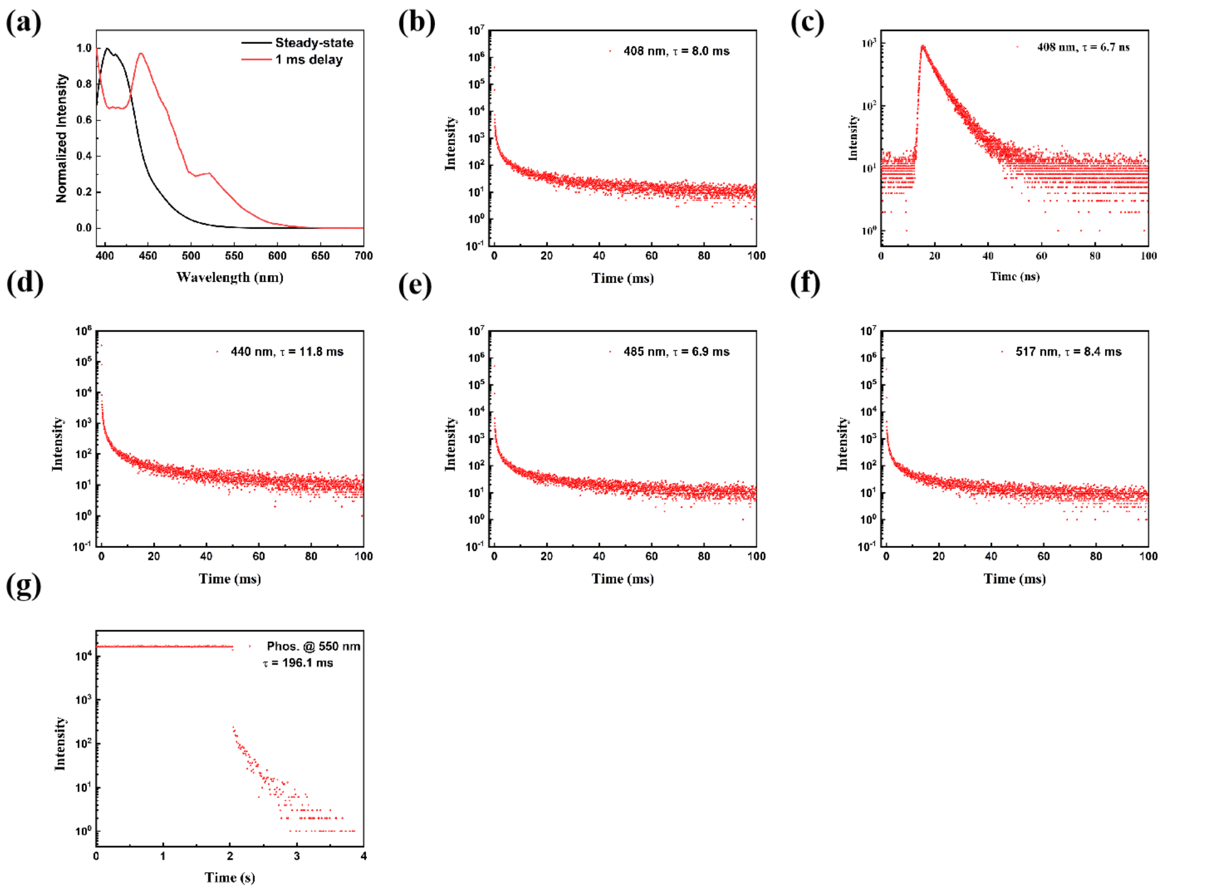

Figure S41. (a) PL spectra of the N-1@PMMA film (1 wt.%); and (b-g) decay spectra at ambient condition. (λ_{ex} = 365 nm)

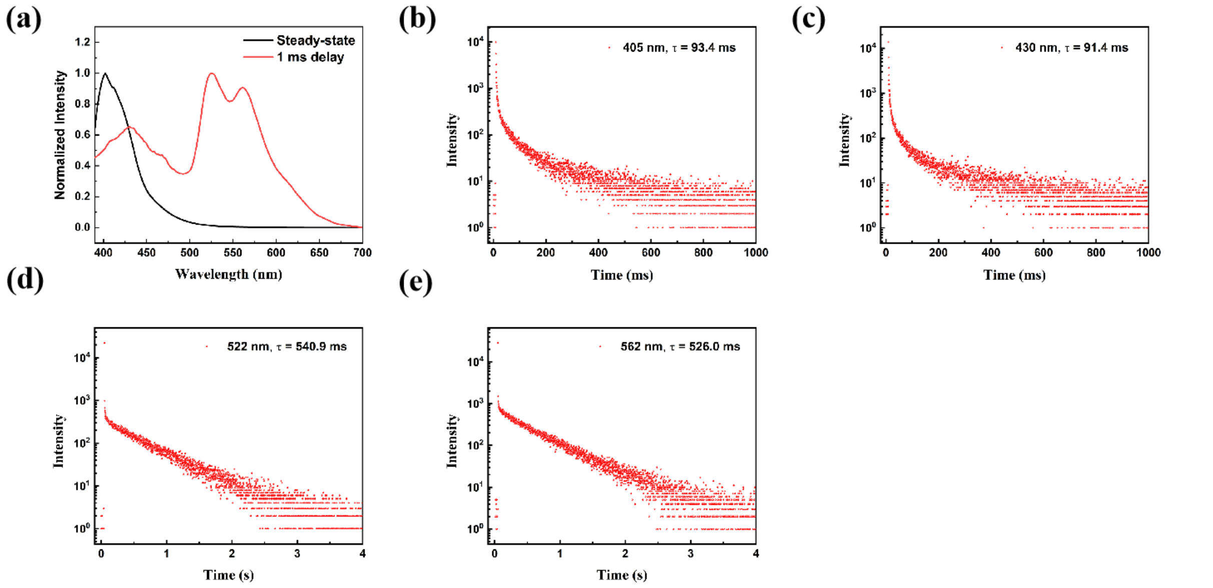

Figure S42. (a) PL spectra of the N-1@PMMA film (1 wt.%); and (b-e) decay spectra at 77 K. (λ_{ex} = 365 nm)

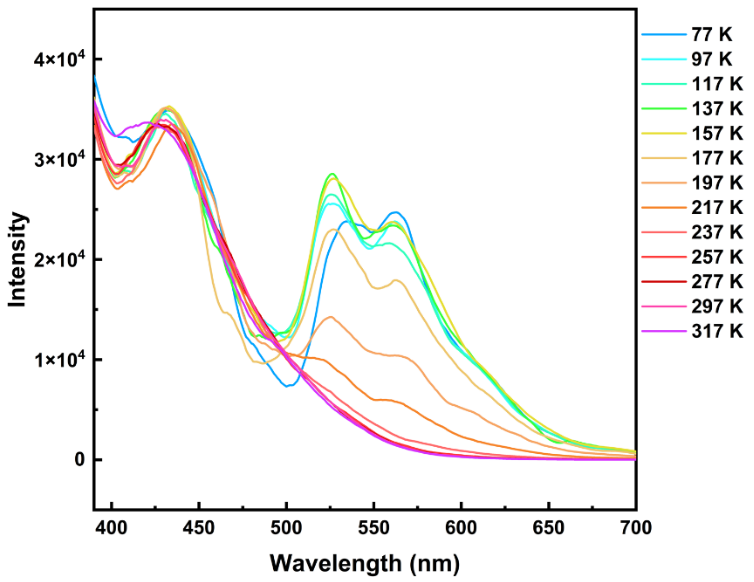

Figure S43. Variable-temperature delayed PL spectra of the N-1@PMMA film (1 wt.%). (λ_{ex} = 365 nm)

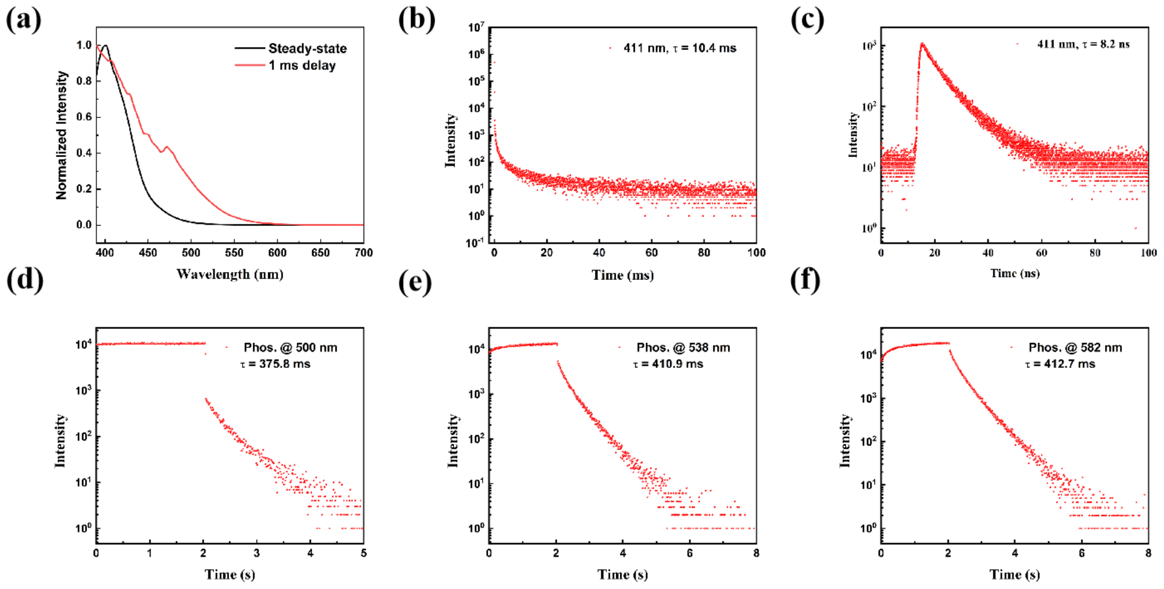

Figure S44. (a) PL spectra of the N-2@PMMA film (1 wt.%); and (b-f) decay spectra at ambient condition. (λ_{ex} = 365 nm)

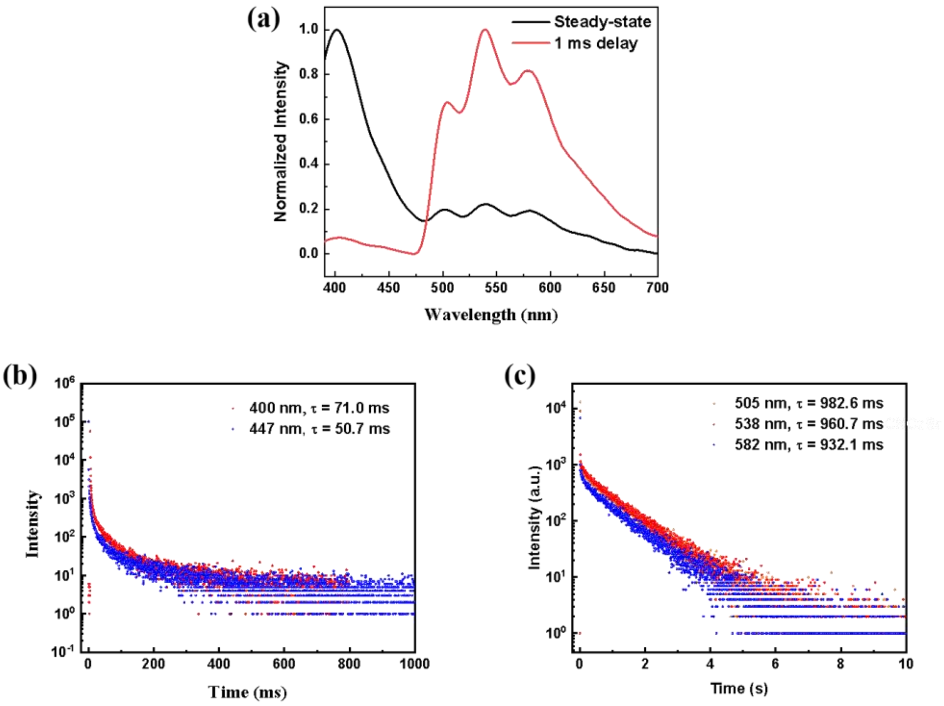

Figure S45. (a) PL spectra of the N-2@PMMA film (1 wt.%); and (b, c) decay spectra at 77 K. (λ_{ex} = 365 nm)

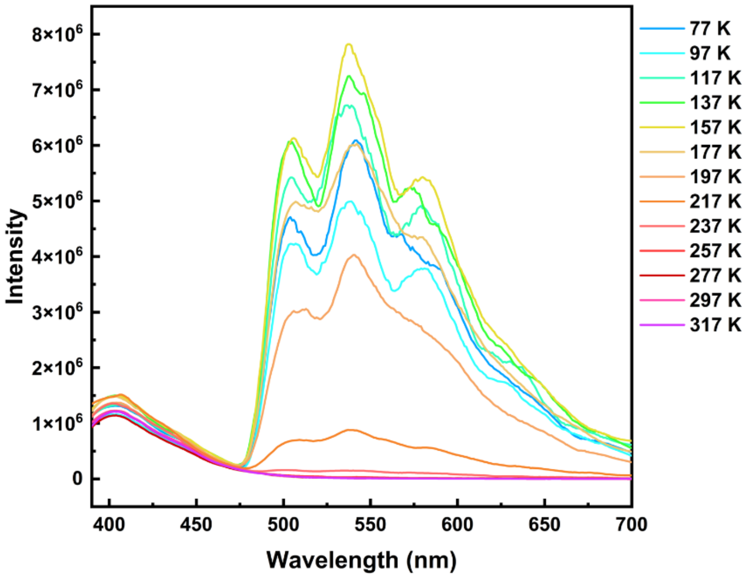

Figure S46. Variable-temperature delayed PL spectra of the N-2@PMMA film (1 wt.%). (λ_{ex} = 365 nm)

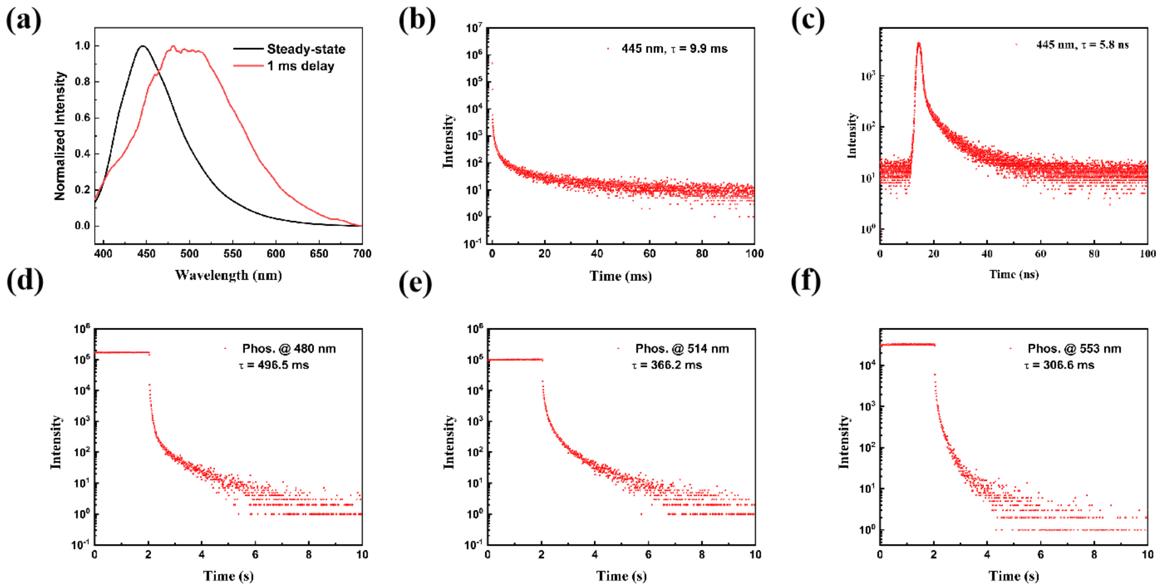

Figure S47. (a) PL spectra of the PNNA-1@PMMA film (1 wt.%); and (b-f) decay spectra at ambient condition. (λ_{ex} = 365 nm)

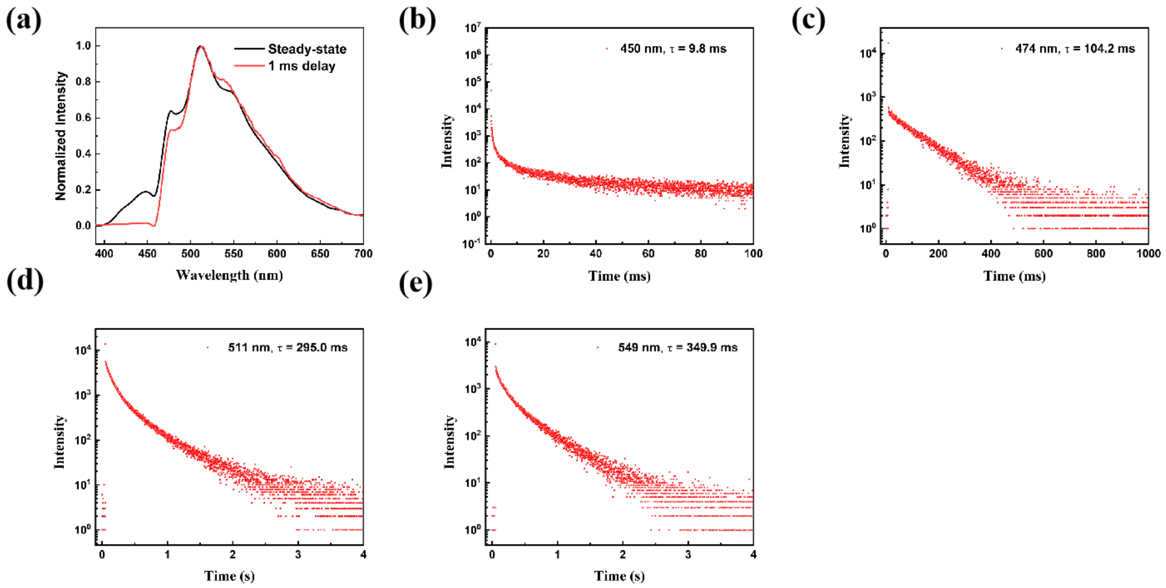

Figure S48. (a) PL spectra of the PNNA-1@PMMA film (1 wt.%); and (b-e) decay spectra at 77 K. (λ_{ex} = 365 nm)

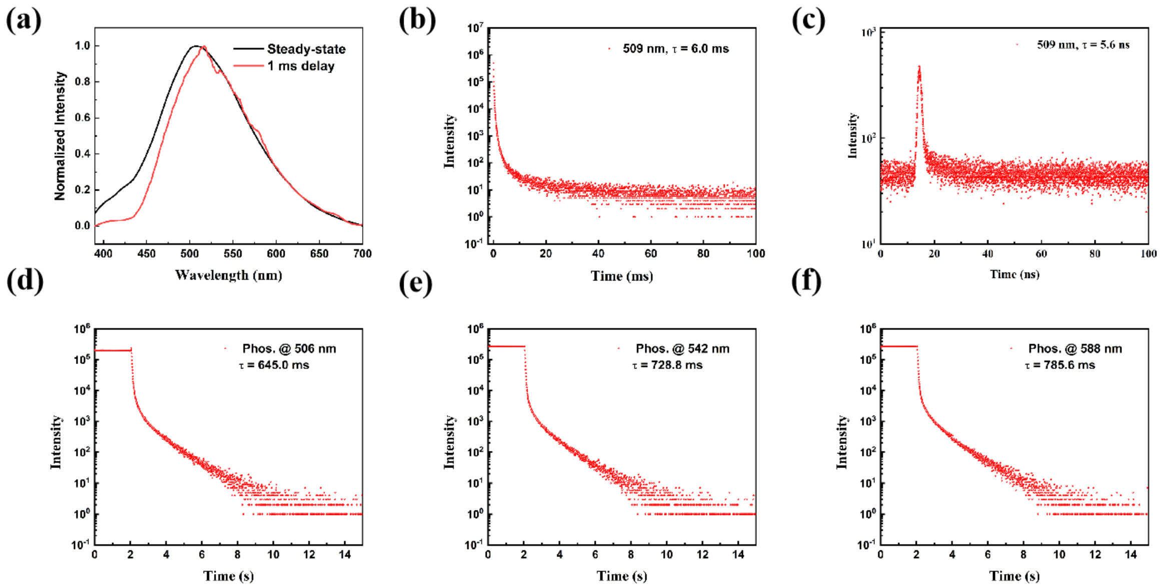

Figure S49. (a) PL spectra of the PNNA-2@PMMA film (1 wt.%); and (b-f) decay spectra at ambient condition. (λ_{ex} = 365 nm)

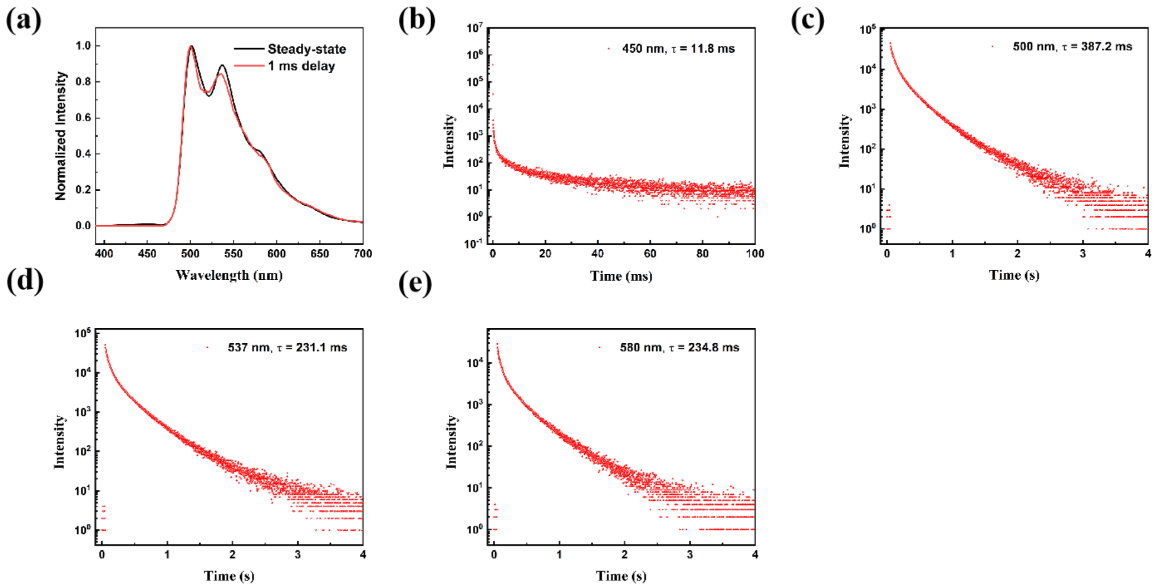

Figure S50. (a) PL spectra of the PNNA-2@PMMA film (1 wt.%); and (b-e) decay spectra at 77 K. (λ_{ex} = 365 nm)

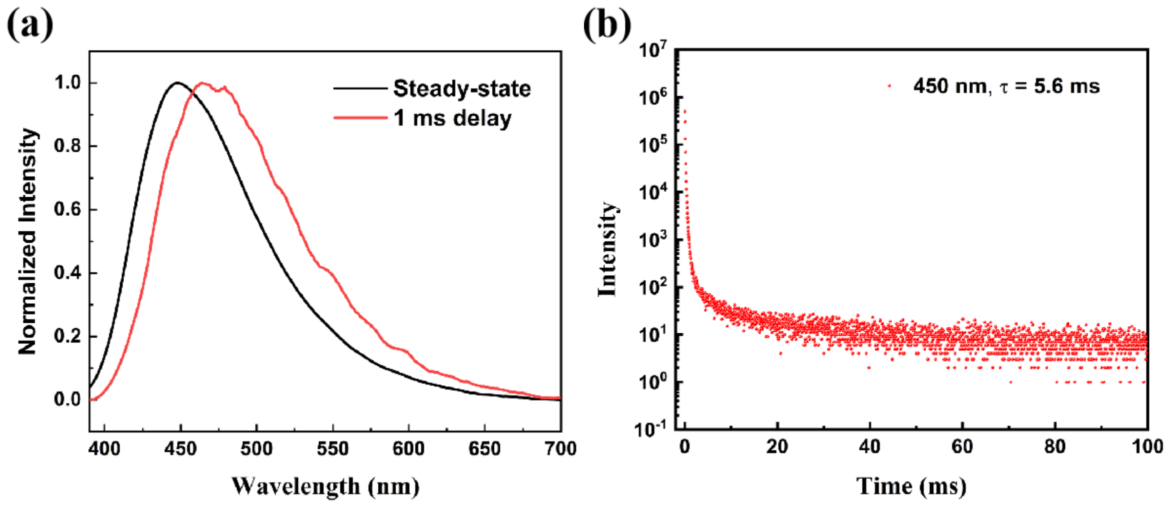

Figure S51. (a) PL spectra of the PNCz@PMMA film (1 wt.%); and (b) decay spectrum at ambient condition. (λ_{ex} = 365 nm)

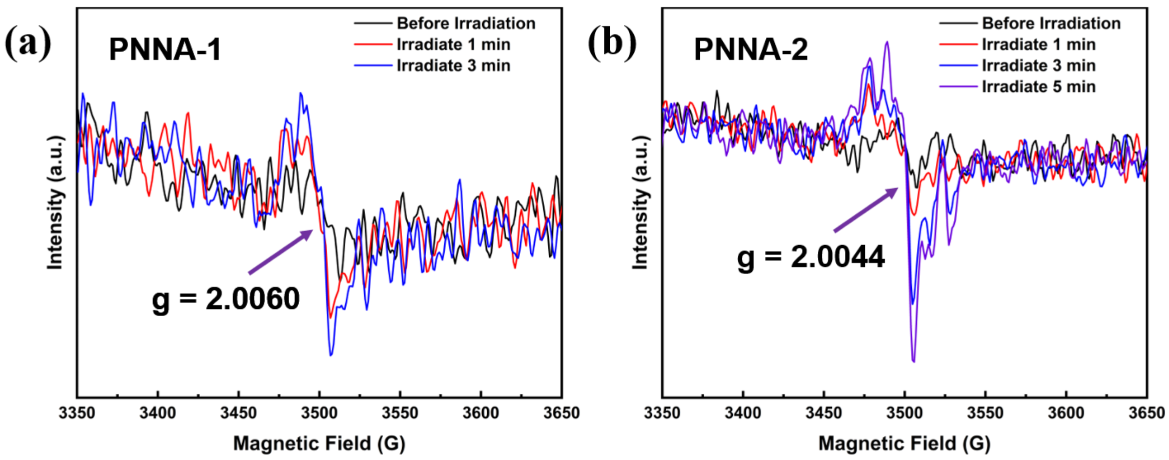

Figure S52. ESR spectra of (a) the PNNA-1@PMMA film (1 wt.%); and (b) the PNNA-2@PMMA film (1 wt.%) before irradiation and after irradiation at ambient condition. (λ_{ex} = 365 nm)

7. **Doping PNNA derivatives into PNCz**.

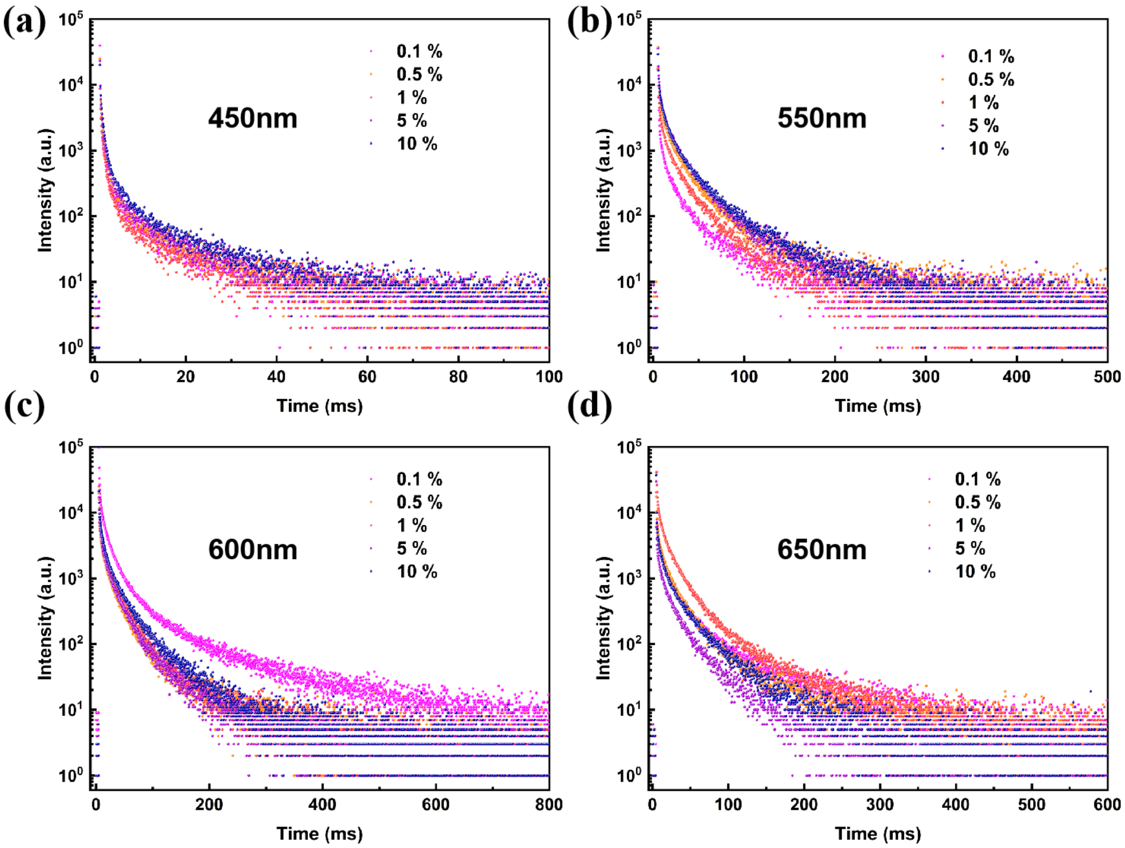

Figure S53. Decay spectra of the PNNA-1@PNCz powder with different dopant ratio (PNNA-1 : PNCz) at (a) 450 nm; (b) 550 nm; (c) 600 nm; and (d) 650 nm at ambient condition. (λ_{ex} = 365 nm)

Table S1. Emission lifetimes of the doped system PNNA-1@PNCz at different wavelength under ambient condition. (λ_{ex} = 365 nm)

PNCz : PNNA-1	1000 : 1	1000 : 5	100 : 1	100 : 5	100 : 10
450 nm	5.7 ms	6.5 ms	6.0 ms	13.1 ms	15.8 ms
550 nm	44.0 ms	50.9 ms	60.6 ms	45.3 ms	47.8 ms
600 nm	49.8 ms	45.2 ms	65.0 ms	45.2 ms	47.2 ms
650 nm	43.6 ms	47.6 ms	73.2 ms	43.4 ms	42.3 ms

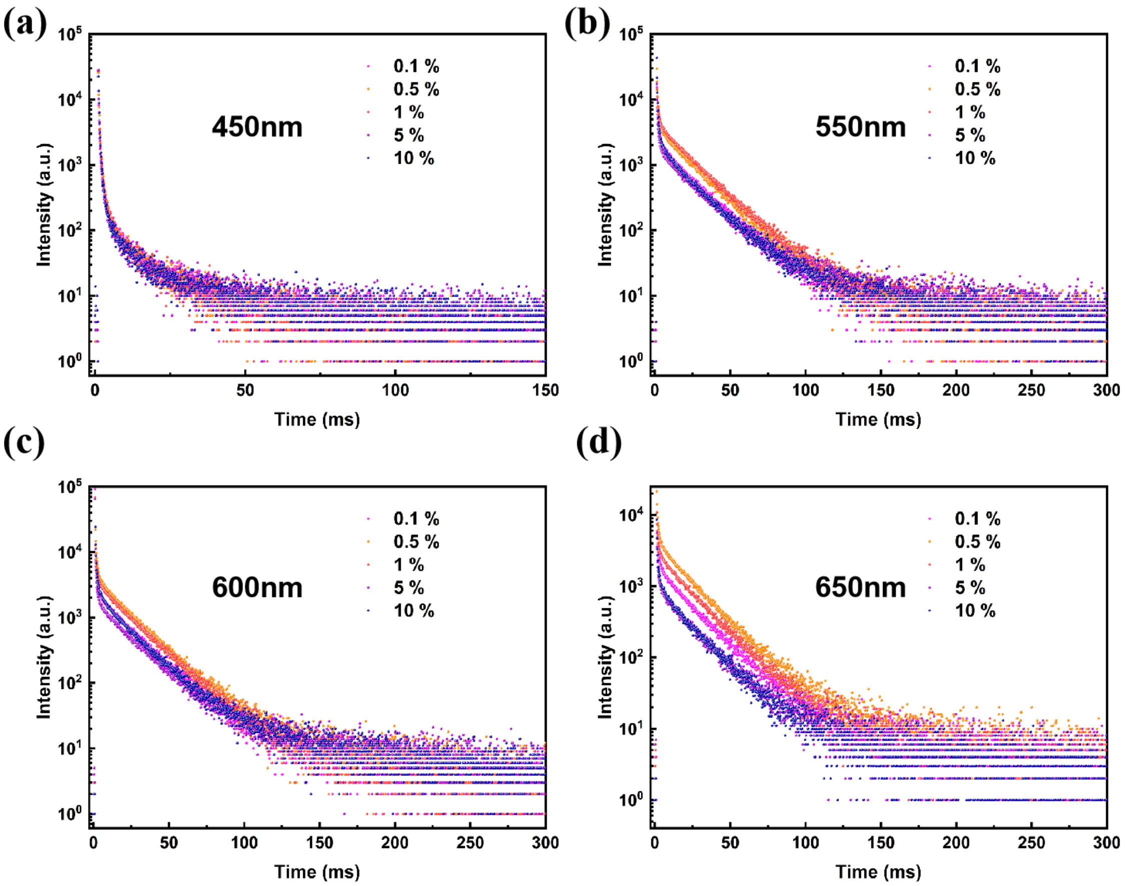

Figure S54. Decay spectra of the PNNA-2@PNCz powder with different dopant ratio (PNNA-2 : PNCz) at (a) 450 nm; (b) 550 nm; (c) 600 nm; and (d) 650 nm at ambient condition. (λ_{ex} = 365 nm)

Table S2. Emission lifetimes of the doped system PNNA-2@PNCz at different wavelength under ambient condition. (λ_{ex}=365 nm)

PNCz : PNNA-2	1000 : 1	1000 : 5	100 : 1	100 : 5	100 : 10
450 nm	7.6 ms	10.4 ms	11.7 ms	12.1 ms	8.5 ms
550 nm	21.3 ms	21.1 ms	21.7 ms	24.8 ms	23.8 ms
600 nm	22.3 ms	21.1 ms	21.3 ms	27.0 ms	24.8 ms
650 nm	21.3 ms	21.8 ms	21.2 ms	24.0 ms	22.0 ms

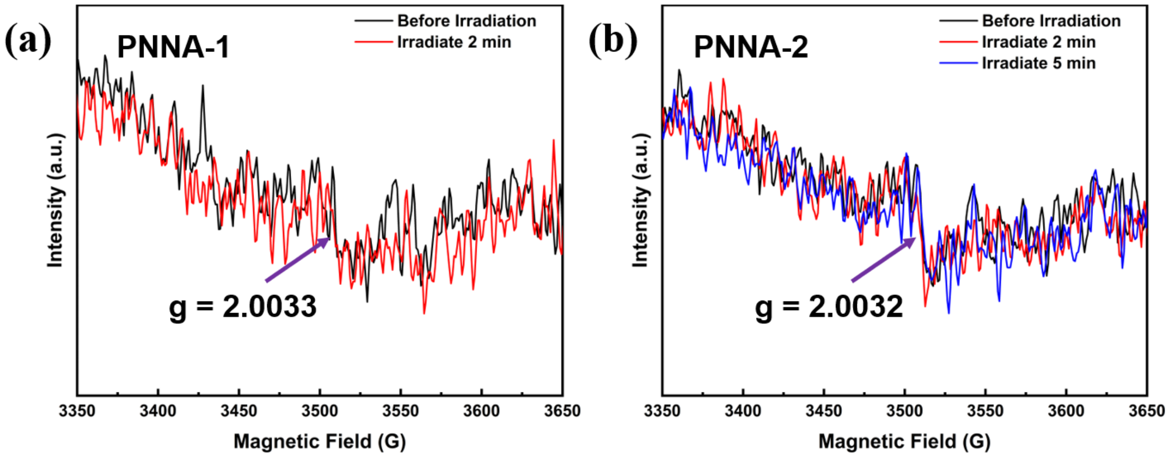

Figure S55. ESR spectra of (a) PNNA-1@PNCz (10 wt.%) and (b) PNNA-2@PNCz (10 wt.%) before irradiation and after irradiation at ambient condition. (λ_{ex} = 365 nm)

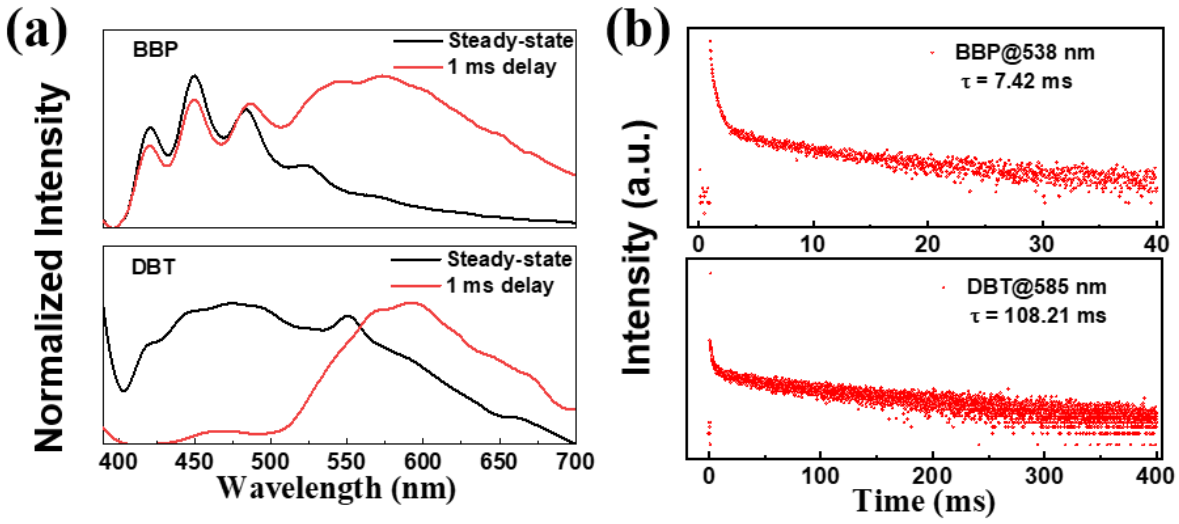

Figure S56. (a) Steady-state and delayed PL spectra, and (b) decay spectra of the pure BBP, and pure DBT powder at ambient condition. (λ_{ex} = 365 nm)

8. **Data table of single crystal**

Table S3. Detailed data of **PNNA-1** and **PNCz** single crystal.

Identification code	PNNA-1	PNCz
CCDC Number	2202532	2202531
Empirical formula	$C_{22}H_{14}BrN_3$	$C_{18}H_{10}BrN_3$
Formula weight	400.27	348.20
Temperature	122.95 K	122.75 K
Wavelength	0.71073 Å	0.71073 Å
Crystal system	monoclinic	monoclinic
Space group	*P 1 21/n 1*	*P 1 21/c 1*
Unit cell dimensions	a = 5.8399(3) Å, α= 90.00°	a = 9.5655(5) Å, α= 90.00°
	b = 20.5015(14) Å, β= 92.357(5)°.	b = 11.8358(7) Å, β= 96.405(5)°.
	c = 14.7074(7) Å, γ = 90.00°.	c = 12.7769(10) Å, γ = 90.00°.
Volume	1759.36(17) $Å^3$	1437.53(16) $Å^3$
Z	4	4
Density (calculated)	1.511 Mg/m^3	1.609 Mg/m^3
Absorption coefficient	2.346 mm^{-1}	2.858 mm^{-1}
F(000)	808	696
Crystal size	0.37 × 0.33 × 0.29 mm^3	0.40 × 0.35 × 0.35 mm^3
Theta range for data collection	3.6930 to 25.1490°	4.2790 to 27.5040°
Index ranges	-7<=h<=6, -17<=k<=25, -17<=l<=18	-11<=h<=9, -14<=k<=14, -15<=l<=15
Reflections collected	3380	2770
Independent reflections	2467 [R(int) = 0.0439]	2071 [R(int) = 0.0478]
Final R indices [I>2sigma(I)]	R1 =0.0476, wR2 =0.0771	R1 =0.0470, wR2 =0.0856
R indices (all data)	R1 =0.0783, wR2 =0.0872	R1 =0.0709, wR2 =0.0958

9. **Unit cell in the single crystal**

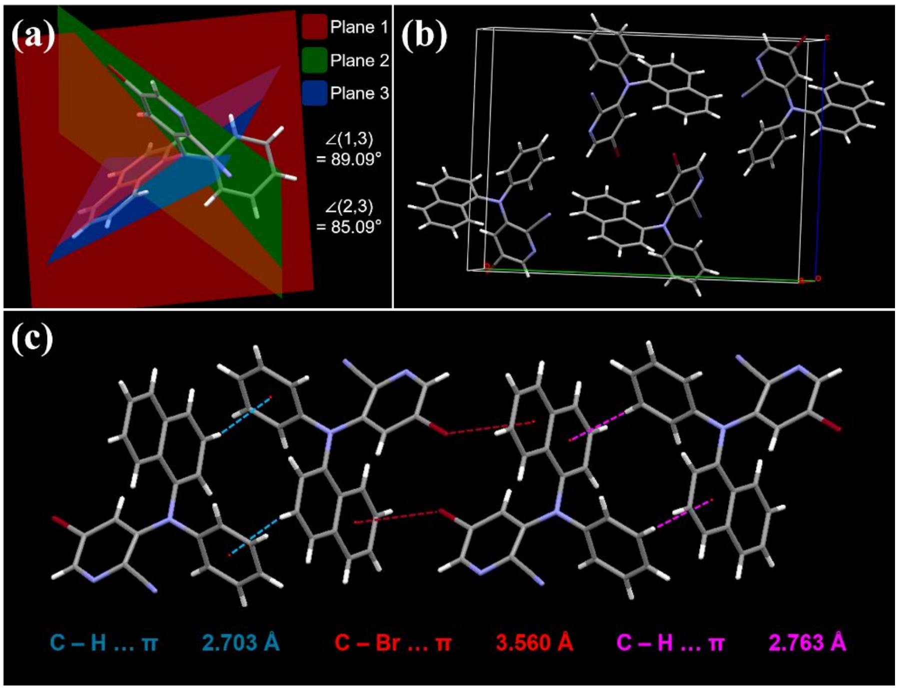

Figure S57. (a) Molecular geometry in single crystals of PNNA-1; (b) molecular packing of PNNA-1 single crystals in the unit cells; (c) the intermolecular interactions between adjacent molecules.

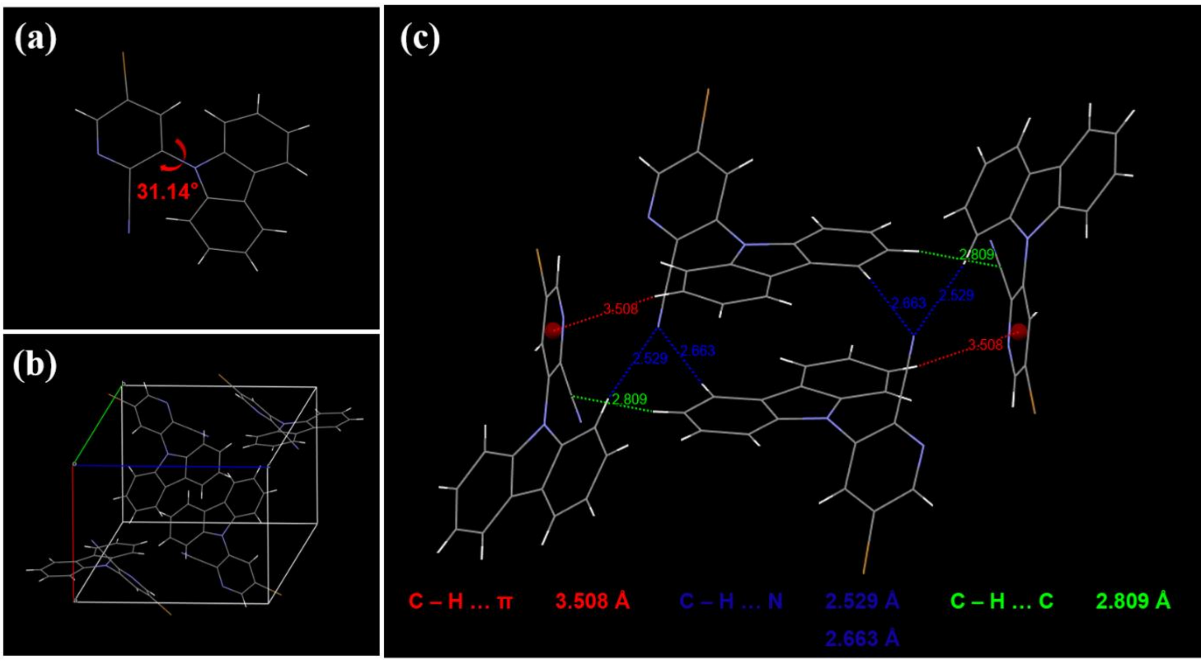

Figure S58. (a) Molecular geometry in single crystals of PNCz; (b) molecular packing of PNCz single crystals in the unit cells; (c) the intermolecular interactions between adjacent molecules.

10. **TD-DFT results**

Table S4. The singlet and triplet excited state transition configurations of the N-1 monomer. The matched excited states that contain the same orbital transition components of S_1 are shown in red

Excited State	Energy (eV)	Transition configuration (%)
T_1	1.7243	H-1→L (6.73), H→L (91.98)
**S_1**	**2.9216**	**H→L (97.83)**
T_2	3.1547	H-4→L (5.37), H-4→L+3 (2.39), H-1→L (49.31), H→L (3.25), H→L+1 (5.06) , H→L+3 (23.45)

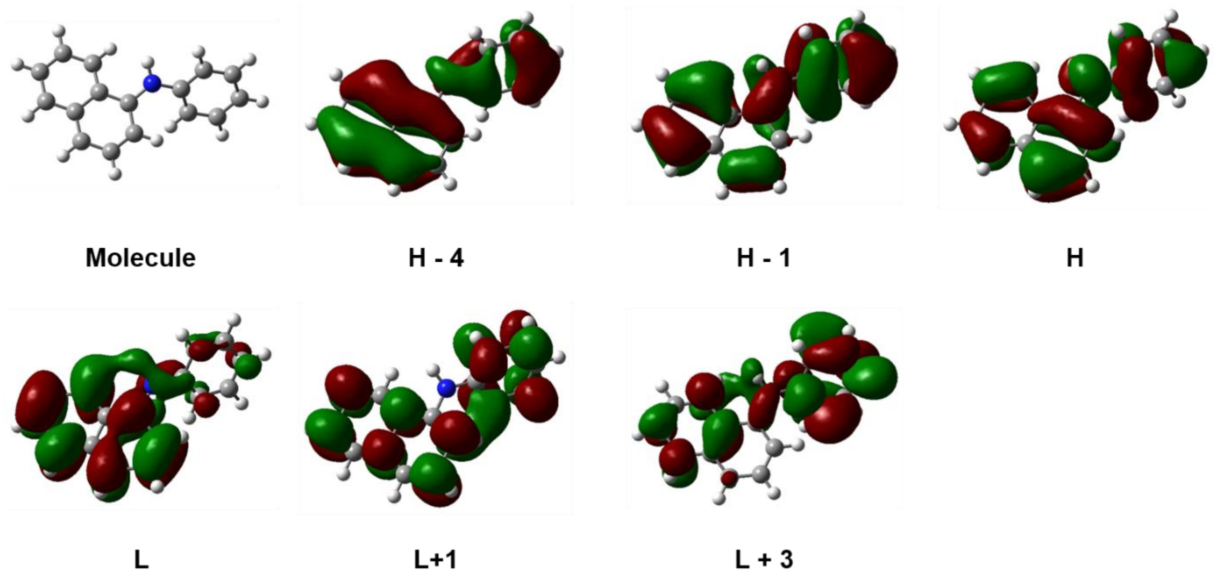

Figure S59. Plots of the N-1 monomer visualized orbitals.

Table S5. The singlet and triplet excited state transition configurations of the N-2 monomer. The matched excited states that contain the same orbital transition components of S_1 are shown in red

Excited State	Energy (eV)	Transition configuration (%)
T_1	1.8356	H-1→L (14.61), H→L (83.84)
**S_1**	**3.0614**	**H-1→L (2.06), H→L (95.49)**
T_2	3.0665	H-3→L (2.11), H-1→L (67.63), H→L (11.32), H→L+1 (6.18), H→L+1 (3.02)
T_3	3.3452	H-3→L+2 (3.23), H-1→L (4.70), H-1→L+1 (4.35), H-1→L+4 (2.61), H→L (2.68), H→L+1 (69.17)

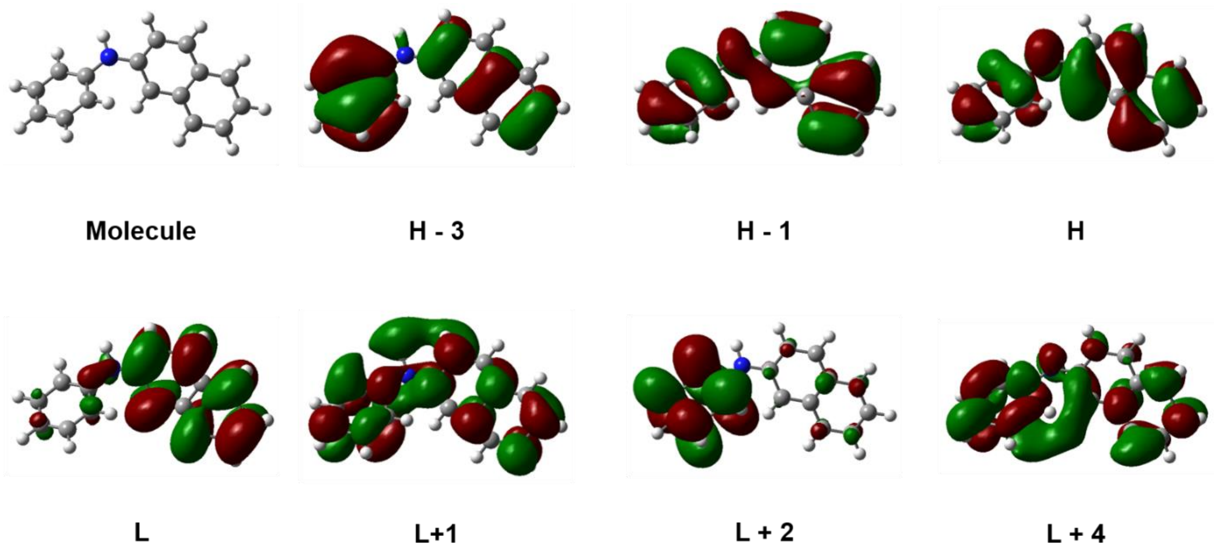

Figure S60. Plots of the N-2 monomer visualized orbitals.

Table S6. The singlet and triplet excited state transition configurations of the PNNA-1 monomer. The matched excited states that contain the same orbital transition components of S_1 are shown in red.

Excited State	Energy (eV)	Transition configuration (%)
T_1	2.5385	H-1→L+1 (14.23), H-1→L+2 (4.31), H→L (6.03), H→L+1 (62.26), H→L+2 (5.20)
T_2	2.7690	H-3→L (3.08), H-1→L (11.05), H→L (75.11), H→L+1 (2.47), H→L+2 (4.44)
T_3	2.9932	H-3→L (4.03), H-1→L+1 (6.59), H-1→L+2 (7.21), H→L (4.86), H→L+1 (11.06), H→L+2 (55.69)
**S_1**	**3.1508**	**H→L (98.47)**
T_4	3.4426	H-4→L (3.35), H-4→L+3 (2.89), H-4→L+5 (2.69), H-3→L (12.17), H-3→L+2 (3.47), H-1→L (11.56), H-1→L+1 (10.79), H-1→L+2 (2.22), H→L (2.07), H→L+1 (2.33), H→L+2 (8.33), H→L+4 (3.71), H→L+5 (2.50), H→L+6 (13.40)

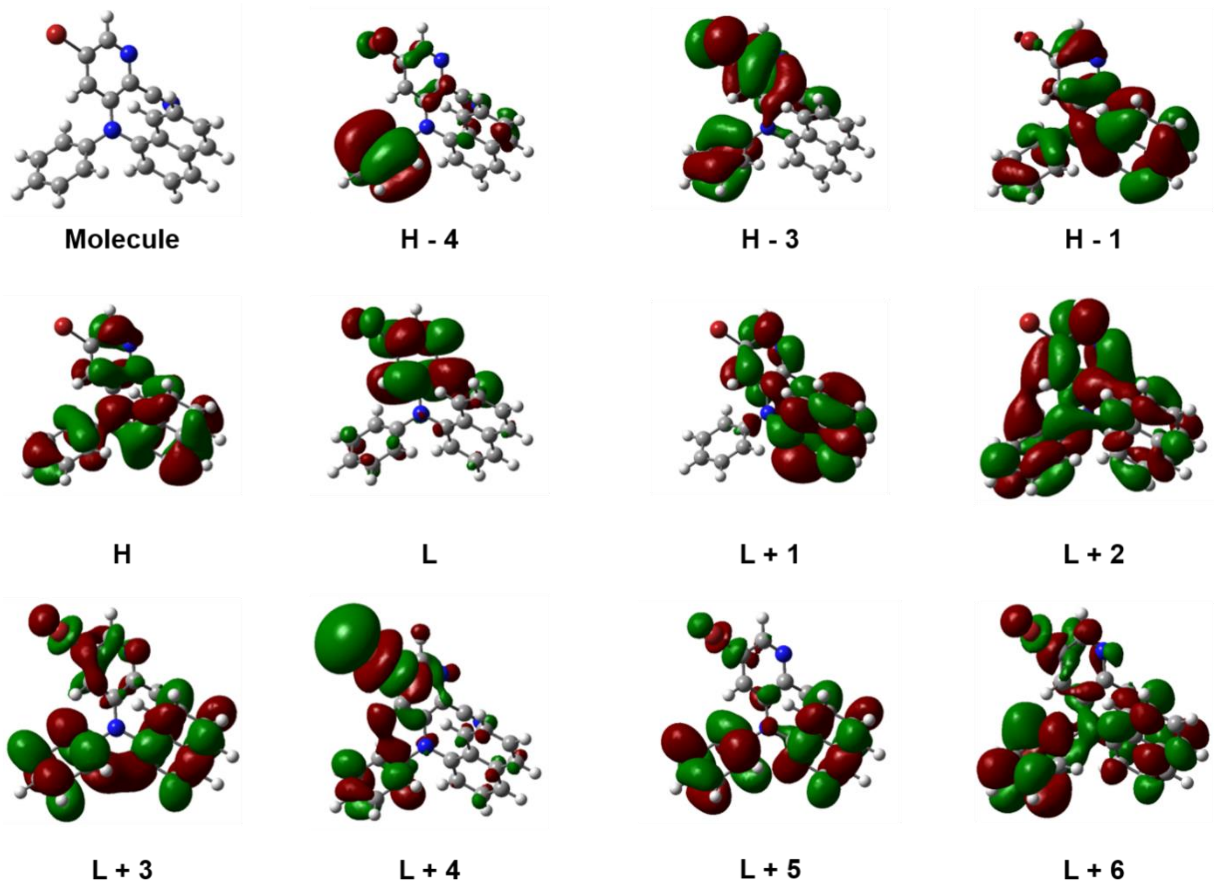

Figure S61. Plots of the PNNA-1 monomer visualized orbitals.

Table S7. The singlet and triplet excited state transition configurations of the PNNA-2 monomer. The matched excited states that contain the same orbital transition components of S_1 are shown in red.

Excited State	Energy (eV)	Transition configuration (%)
T_1	1.7363	H→L (97.61)
**S_1**	**1.7832**	**H→L (98.94)**
T_2	2.3902	H-1→L+2 (3.26), H→L+1 (23.05), H→L+2 (47.08), H→L+3 (12.93), H→L+5 (3.62), H→L+7 (2.35)
T_3	2.6911	H-4→L (3.16), H→L+1 (67.98), H→L+2 (15.85), H→L+3 (4.96)
**S_2**	**2.7277**	**H→L+1 (97.46)**
T_4	2.8073	H-12→L (2.69), H-4→L (49.50), H-3→L (7.49), H-2→L (3.05), H-1→L (26.19), H→L+1 (5.31)
S_3	2.9696	H-1→L (98.08)
T_5	2.9842	H-4→L (20.38), H-3→L (2.37), H-1→L (68.97)

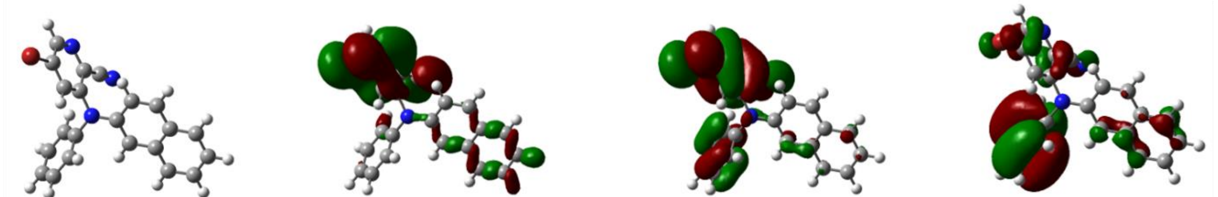

Figure S62. Plots of the PNNA-2 monomer visualized orbitals.

Table S8. Spin-orbit coupling (SOC) values between S_n and T_n of the N-1, N-2, PNNA-1 and PNNA-2 dimer.

$\langle S_n \vert Hso \vert T_n \rangle$	N-1 (cm^{-1})	N-2 (cm^{-1})	PNNA-1 (cm^{-1})	PNNA-2 (cm^{-1})
S_0/T_1	0.45870	1.05351	40.95159	92.31138
S_0/T_2	1.46156	1.12296	262.59604	340.10825
S_0/T_3	1.41268	0.76785	176.68936	185.16957
S_0/T_4	-	-	214.94513	111.06126
S_0/T_5	-	-	-	231.28307
S_1/T_1	0.23399	0.66027	64.46871	30.46261
S_1/T_2	0.18629	0.45834	233.80934	224.24207
S_1/T_3	0.95539	0.87320	181.47263	168.48393
S_1/T_4	-	-	141.30225	56.67844
S_1/T_5	-	-	-	709.53632

Table S9. The excited state and oscillator strength of the N-1, N-2, PNNA-1 and PNNA-2

monomer radical.

Excited State	Energy (eV)	Wavelength (nm)	oscillator strength (f)
**N-1**			
**ES1**	**1.6034**	**773.24**	**0.1878**
**N-2**			
ES1	0.6207	1997.56	0.0133
ES2	1.7392	712.87	0.0064
**ES3**	**1.8049**	**686.94**	**0.2928**
**PNNA-1**			
**ES1**	**1.3524**	**916.80**	**0.1049**
ES2	1.6151	767.64	0.0043
ES3	1.7127	723.89	0.0271
ES4	1.9118	648.54	0.0133
ES5	2.2281	556.45	0.0118
ES6	2.4415	507.81	0.0221
**PNNA-2**			
ES1	0.5669	2186.90	0.0194
ES2	1.4652	846.20	0.0182
**ES3**	**1.6312**	**760.07**	**0.1471**
ES4	1.7368	713.85	0.0452
ES5	2.0454	606.17	0.0117
ES6	2.3803	520.88	0.0325
ES7	2.4542	505.20	0.0278

Table S10. Total energy of the N-1, N-2, PNNA-1, PNNA-2 monomer and their radical cations.

	N-1	N-2	PNNA-1	PNNA-2
Monomer (E_M,HF)	-672.464331661	-672.466494071	-3585.39850965	-3585.40257706
Radical ($E_{M^{\cdot+}}$,HF)	-672.216543832	-672.217556934	-3585.13949549	-3585.14593208
ΔE ($E_{M^{\cdot+}} - E_M$, kJ/mol)	650.57	653.58	680.04	673.82

Table S11. Total energy of the PNCz monomer and its radical ions.

	PNCz
Monomer (E_M,HF)	-3430.53833573
Radical ($E_{M^{\cdot-}}$,HF)	-3430.58315529
ΔE ($E_{M^{\cdot-}} - E_M$, kJ/mol)	117.67

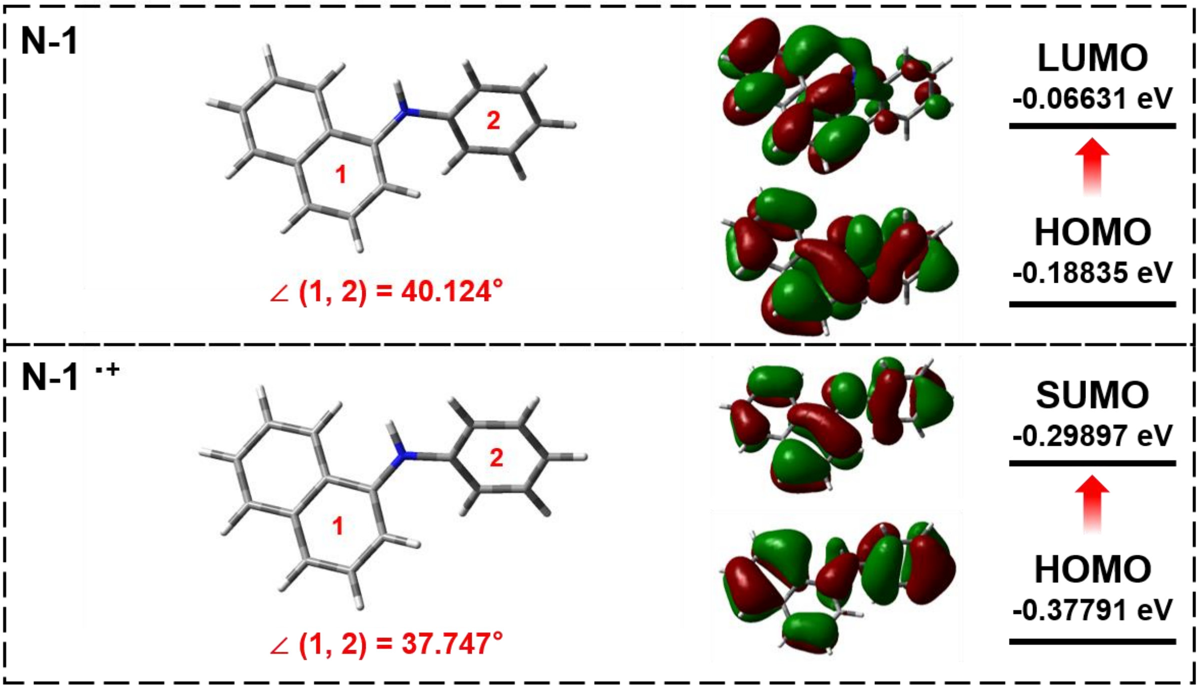

Figure S63. Plots of the N-1 monomer and radical visualized orbitals.

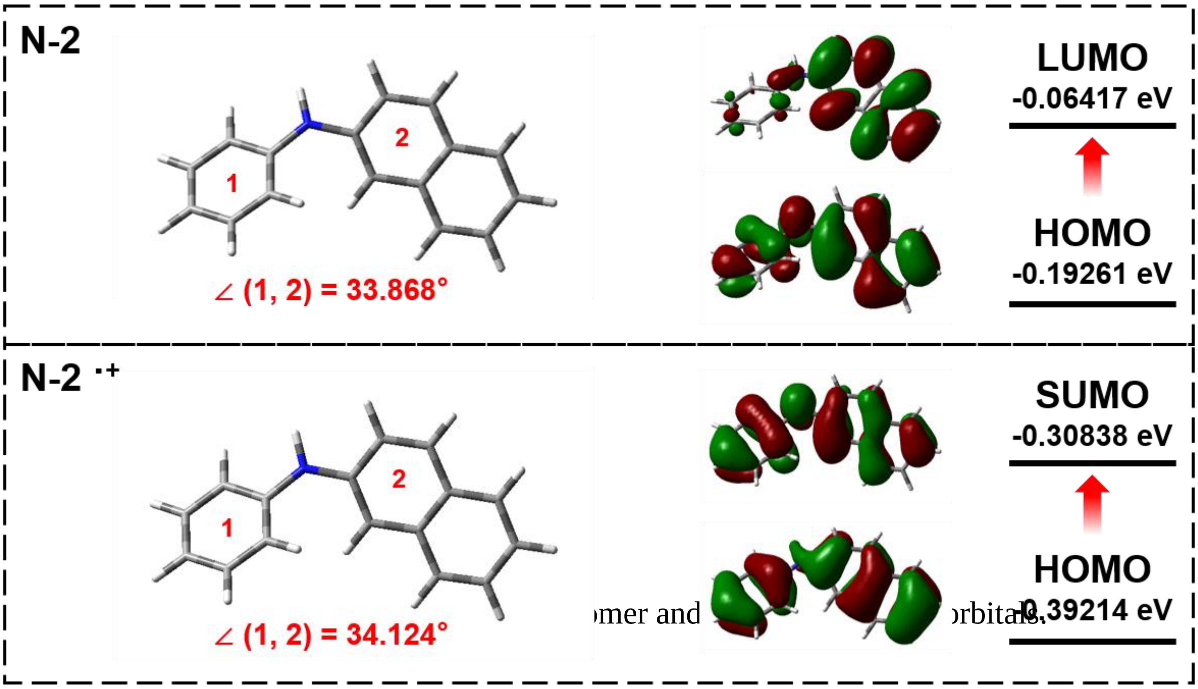

Figure S64. Plots of the N-2 monomer and radical visualized orbitals.

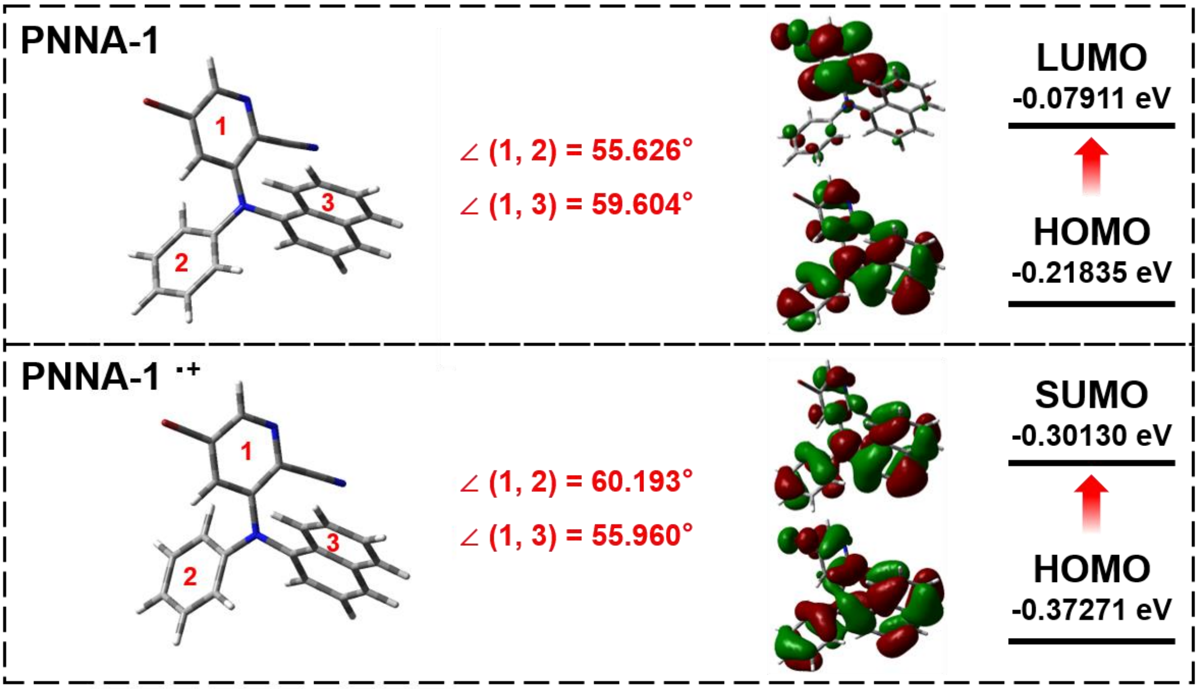

Figure S65. Plots of the PNNA-1 monomer and radical visualized orbitals.

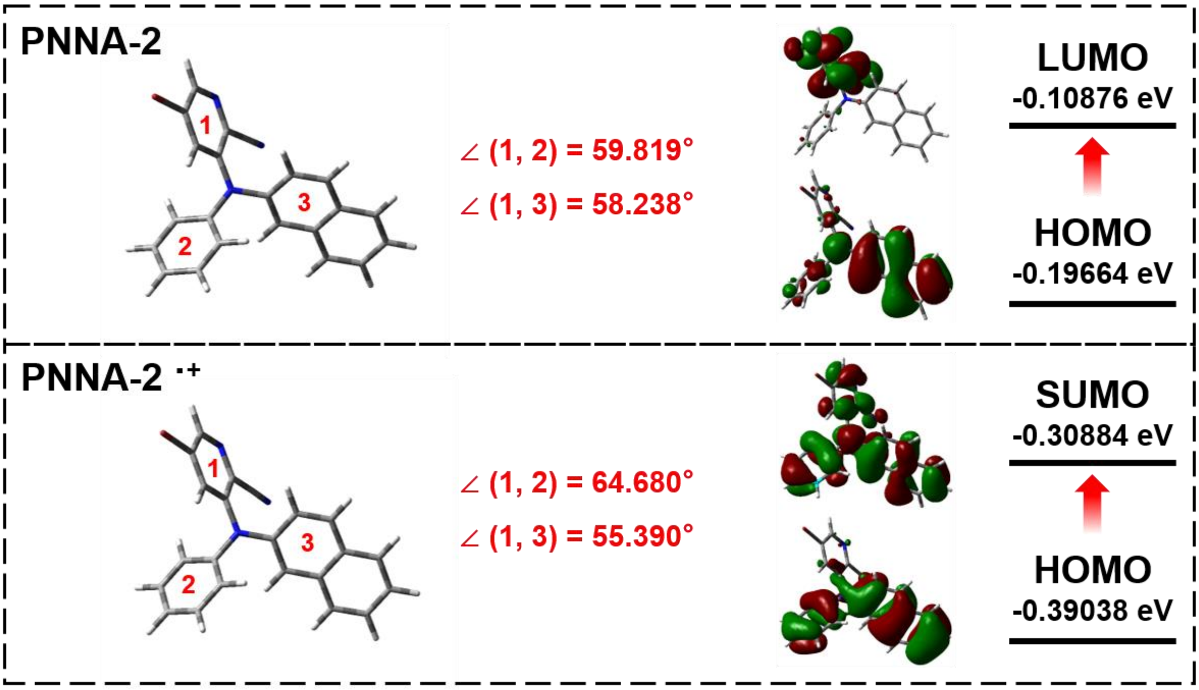

Figure S66. Plots of the PNNA-2 monomer and radical visualized orbitals.

References:

[1] J. Li, J. Zhou, Z. Mao, Z. Xie, Z. Yang, B. Xu, C. Liu, X. Chen, D. Ren, H. Pan, G. Shi, Y. Zhang, Z. Chi, *Angew. Chem. Int. Ed.* **2018**, *57*, 6449-6453.

[2] Z. An, C. Zheng, Y. Tao, R. Chen, H. Shi, T. Chen, Z. Wang, H. Li, R. Deng, X. Liu, W. Huang, *Nat. Mater*. **2015**, *14*, 685-690.

[3] C. Qian, Z. Ma, B. Yang, X. Li, J. Sun, Z. Li, H. Jiang, M. Chen, X. Jia, Z. Ma, *J. Mater. Chem. C* **2021**, *9*, 14294-14302.
