# Supplementary material for: A Class of Organic Units Featuring Matrix‐Controlled Color‐Tunable Ultralong Organic Room Temperature Phosphorescence
Source: Adv Sci (Weinh). 2022 Dec 25;10(3):2206482. doi: 10.1002/advs.202206482 (PMC9875667; doi:10.1002/advs.202206482)

ADVANCED SCIENCE
Open Access

Supporting Information

for *Adv. Sci.*, DOI 10.1002/advs.202206482

A Class of Organic Units Featuring Matrix-Controlled Color-Tunable Ultralong Organic Room Temperature Phosphorescence

*Xue Zhang, Chen Qian, Zhimin Ma, Xiaohua Fu, Zewei Li, Huiwen Jin, Mingxing Chen, Hong Jiang and Zhiyong Ma**

checkCIF/PLATON report

You have not supplied any structure factors. As a result the full set of tests cannot be run.

THIS REPORT IS FOR GUIDANCE ONLY. IF USED AS PART OF A REVIEW PROCEDURE FOR PUBLICATION, IT SHOULD NOT REPLACE THE EXPERTISE OF AN EXPERIENCED CRYSTALLOGRAPHIC REFEREE.

No syntax errors found. CIF dictionary Interpreting this report

Datablock: exp_8089

Bond precision: C-C = 0.0051 A Wavelength=0.71073

Cell:	a=5.8399(3)	b=20.5015(14)	c=14.7074(7)
	alpha=90	beta=92.357(5)	gamma=90
Temperature:	123 K		

	Calculated	Reported
Volume	1759.38(17)	1759.36(17)
Space group	P 21/n	P 1 21/n 1
Hall group	-P 2yn	-P 2yn
Moiety formula	C22 H14 Br N3	C22 H14 Br N3
Sum formula	C22 H14 Br N3	C22 H14 Br N3
Mr	400.26	400.27
Dx,g cm-3	1.511	1.511
Z	4	4
Mu (mm-1)	2.346	2.346
F000	808.0	808.0
F000’	807.10	
h,k,lmax	7,25,18	7,25,18
Nref	3451	3380
Tmin,Tmax	0.437,0.506	0.906,1.000
Tmin’	0.404	

Correction method= # Reported T Limits: Tmin=0.906 Tmax=1.000
AbsCorr = MULTI-SCAN

Data completeness= 0.979 Theta(max)= 26.000

R(reflections)= 0.0476(2467) wR2(reflections)= 0.0872(3380)

S = 1.028 Npar= 266

The following ALERTS were generated. Each ALERT has the format
test-name_ALERT_alert-type_alert-level.
Click on the hyperlinks for more details of the test.

Alert level C

```
PLAT018_ALERT_1_C _diffrn_measured_fraction_theta_max .NE. *_full          ! Check
PLAT213_ALERT_2_C Atom C9              has ADP max/min Ratio .....       3.7 prolat
PLAT220_ALERT_2_C NonSolvent   Resd 1  C   Ueq(max)/Ueq(min) Range       4.1 Ratio
PLAT241_ALERT_2_C High   'MainMol' Ueq as Compared to Neighbors of        C9 Check
PLAT790_ALERT_4_C Centre of Gravity not Within Unit Cell: Resd.  #         1 Note
              C22 H14 Br N3
```

Alert level G

```
PLAT003_ALERT_2_G Number of Uiso or Uij Restrained non-H Atoms ...        12 Report
PLAT005_ALERT_5_G No Embedded Refinement Details Found  in the CIF     Please Do !
PLAT093_ALERT_1_G No s.u.'s on H-positions, Refinement Reported as      mixed Check
PLAT301_ALERT_3_G Main Residue  Disorder .............(Resd  1  )        23% Note
PLAT710_ALERT_4_G Delete 1-2-3 or 2-3-4 Linear Torsion Angle ... #        60 Do !
              N1  -C6  -C5  -C4   -114.00  6.00   1_555   1_555   1_555   1_555
PLAT710_ALERT_4_G Delete 1-2-3 or 2-3-4 Linear Torsion Angle ... #        61 Do !
              N1  -C6  -C5  -N14    63.00  6.00   1_555   1_555   1_555   1_555
PLAT779_ALERT_4_G Suspect or Irrelevant (Bond) Angle(s) in CIF ...      11.30 Deg.
              C17  -N2   -C23     1_555   1_555   1_555 ......     #    9 Check
PLAT860_ALERT_3_G Number of Least-Squares Restraints ............         72 Note
```

0 **ALERT level A** = Most likely a serious problem - resolve or explain
0 **ALERT level B** = A potentially serious problem, consider carefully
5 **ALERT level C** = Check. Ensure it is not caused by an omission or oversight
8 **ALERT level G** = General information/check it is not something unexpected

2 ALERT type 1 CIF construction/syntax error, inconsistent or missing data
4 ALERT type 2 Indicator that the structure model may be wrong or deficient
2 ALERT type 3 Indicator that the structure quality may be low
4 ALERT type 4 Improvement, methodology, query or suggestion
1 ALERT type 5 Informative message, check

It is advisable to attempt to resolve as many as possible of the alerts in all categories. Often the minor alerts point to easily fixed oversights, errors and omissions in your CIF or refinement strategy, so attention to these fine details can be worthwhile. In order to resolve some of the more serious problems it may be necessary to carry out additional measurements or structure refinements. However, the purpose of your study may justify the reported deviations and the more serious of these should normally be commented upon in the discussion or experimental section of a paper or in the "special_details" fields of the CIF. checkCIF was carefully designed to identify outliers and unusual parameters, but every test has its limitations and alerts that are not important in a particular case may appear. Conversely, the absence of alerts does not guarantee there are no aspects of the results needing attention. It is up to the individual to critically assess their own results and, if necessary, seek expert advice.

Publication of your CIF in IUCr journals

A basic structural check has been run on your CIF. These basic checks will be run on all CIFs submitted for publication in IUCr journals (*Acta Crystallographica*, *Journal of Applied Crystallography*, *Journal of Synchrotron Radiation*); however, if you intend to submit to *Acta Crystallographica Section C* or *E* or *IUCrData*, you should make sure that full publication checks are run on the final version of your CIF prior to submission.

Publication of your CIF in other journals

Please refer to the *Notes for Authors* of the relevant journal for any special instructions relating to CIF submission.

PLATON version of 18/05/2022; check.def file version of 17/05/2022

Datablock exp_8089 - ellipsoid plot

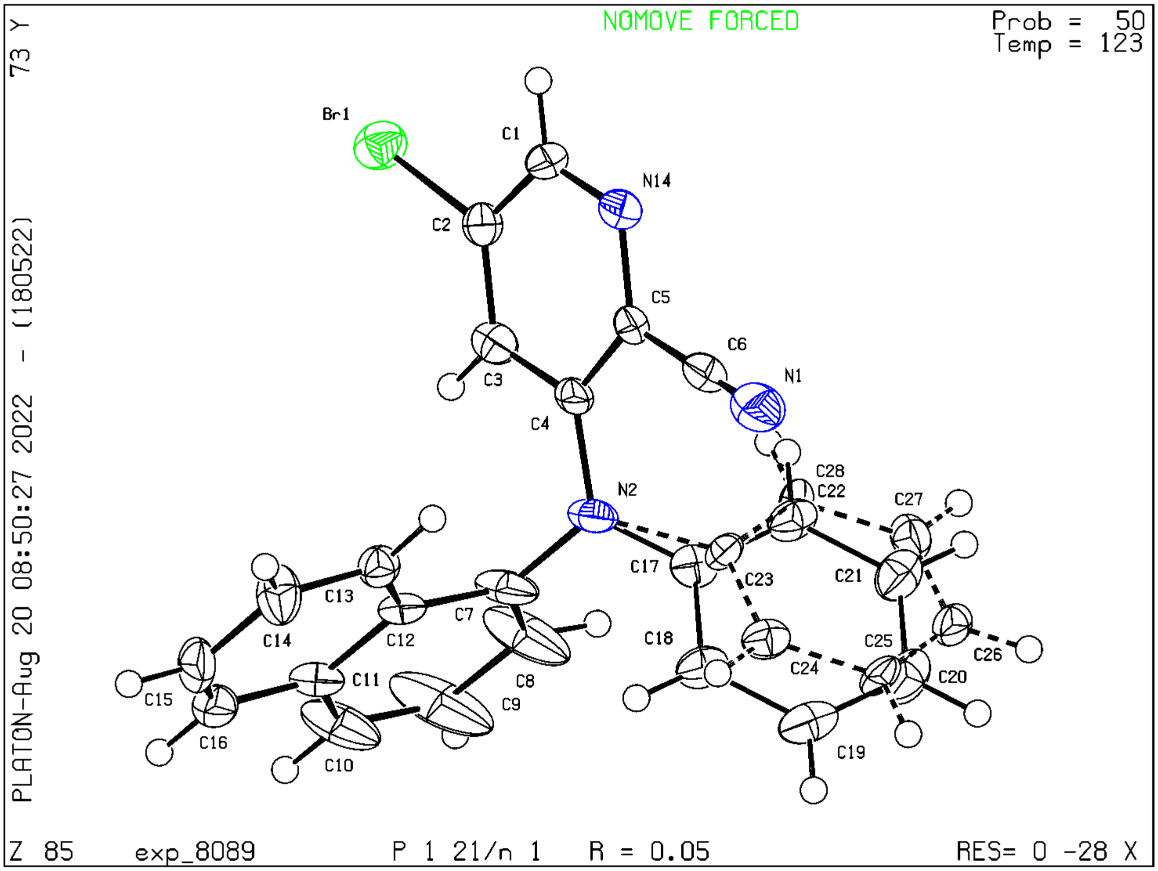

Supplement: Supplementary file 5 — Supporting Information [file ADVS-10-2206482-s003.pdf]
